# Six Decades of Forest Inventory Data Highlight Decline of Sugar Maple (*Acer saccharum*) Sapling Abundance in Eastern Canada

**DOI:** 10.1002/ece3.71386

**Published:** 2025-05-15

**Authors:** Martin‐Michel Gauthier

**Affiliations:** ^1^ Natural Resources Canada Canadian Forest Service Ottawa Ontario Canada

**Keywords:** *Acer saccharum*, American beech, Canada, decline, *Fagus grandifolia*

## Abstract

Six decades of temporal changes in the abundance of sugar maple ( *Acer saccharum* Marsh.) were investigated using a network of multi‐agency ground plots (MAGPlots) located across Ontario, Québec, and New Brunswick, Canada. Based on a composite dataset of nearly 400 plots mainly composed of sugar maple trees (≥ 50% basal area, m^2^ ha^−1^), results showed that the relative abundance (% total sapling basal area) of sugar maple saplings declined significantly over time. On average, the relative abundance of sugar maple saplings decreased significantly between 1970 and 2022. Out of a wide range of potential explanatory variables, including stand conditions, harvest intensity (0%–92% basal area removal), regional ecozones, and climate variables, the relative abundance of American beech ( *Fagus grandifolia* Ehrh.) saplings was the only variable that had a negative effect on the relative abundance of sugar maple saplings. The plot‐specific distribution of change between the final and initial measurements over time revealed that many plots showing a decline in relative sugar maple sapling abundance also experienced an increase in relative American beech sapling abundance. The lack of differences between harvested and unharvested plots suggests that beech sapling control in the understory and soil liming treatments may be required to help promote sugar maple regeneration and development.

## Graphical abstract

Approximate (±10 km) geographic location of the 390 plots among the Atlantic Highlands, Mixedwood Plains, and Boreal Shield ecozones of eastern Canada. Six decades of forest inventory data showed a significant decline in the relative abundance of sugar maple ( *Acer saccharum* ) saplings between 1970 and 2022.

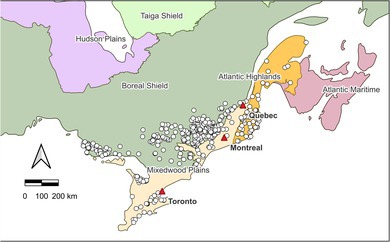

## 1 Introduction

Sugar maple ( *Acer saccharum* Marsh.) is an emblematic and culturally significant tree species found in temperate deciduous forests of eastern Canada and the United States (Stern et al. 2023; Leduc et al. 2024). It provides many socio‐economic benefits, including maple syrup, wood products, carbon sequestration, watershed protection, tourism, and recreational opportunities (Leak et al. 2014). Forests dominated by sugar maple also have structural complexity attributes, such as downed coarse woody debris and large cavity trees, that provide wildlife habitat and other ecosystem services (Coombs et al. 2010; Gauthier et al. 2019). In the absence of forest management, sugar maple and American beech ( *Fagus grandifolia* Ehrh.) tend to dominate temperate deciduous forests because of their shade tolerance and longevity, coupled with gap disturbance dynamics like windthrow and tree mortality due to senescence (Canham 1988, 1989). Stand‐replacing disturbances are rare (Fraver et al. 2009). Forest management attempts to emulate natural disturbances and capture mortality with partial harvests carried out at regular intervals (Seymour et al. 2002; Leak et al. 2014). Harvesting is aimed at improving growth and recruitment of sugar maple regeneration (Nyland 1996). Recent findings indicate high intensity partial harvests (41%–80% basal area (BA) removal) increase sapling recruitment and mortality compared to unharvested stands (Bose et al. 2020). Sugar maple sapling growth, however, appears to be lower than the growth of American beech saplings even at harvest intensities reaching 80% (Leduc et al. 2024).

In recent decades, several biotic and abiotic factors negatively impacted sugar maple growth and abundance in managed and unmanaged forests. They include atmospheric acid deposition that depleted soil base cations (Sullivan et al. 2013; Cleavitt et al. 2018; Stern et al. 2023), preferential browsing by deer ( *Odocoileus virginianus* Zimm.) (Long et al. 2007; Bose et al. 2018), changing climate (Bose et al. 2017a; Boakye et al. 2023), increases in the frequency and severity of thaw–freeze and drought events (Moreau et al. 2020), and insect and pathogen damage (Cleavitt et al. 2011, 2014). American beech abundance increased during the same period, particularly in the sapling layer (e.g., Duchesne and Ouimet 2009; Gravel et al. 2011; Gauthier et al. 2015). Compared to sugar maple, American beech is more shade tolerant (Hane 2003), has greater asexual reproduction capacity through root suckering and stump sprouting (Beaudet and Messier 2008), is more efficient in assimilating carbon under elevated atmospheric CO_2_ (Reid and Strain, 1994), and is more tolerant of soil acidity (Duchesne et al. 2005).

Given these challenges, identifying long‐term impacts of forest management on natural regeneration dynamics is important to maintain the socio‐economic and ecological benefits of sugar maple in temperate deciduous forests. Knowledge gaps still exist regarding changes in sugar maple abundance over large temporal and spatial scales, especially under operational settings with a wide range of partial harvest intensities. Only a handful of published studies tracked the spatial and temporal change of sugar maple over five decades or more using permanent plots in the northeastern USA (e.g., Pontius et al. 2016; Ducey et al. 2024). This knowledge gap has not been studied extensively in Canada. It is especially important considering the use of natural regeneration for stand renewal (Bose et al. 2020). Integrating influences of site and environmental factors in the context of species abundance over six decades could help identify important drivers of change in these forests.

**FIGURE 1:**
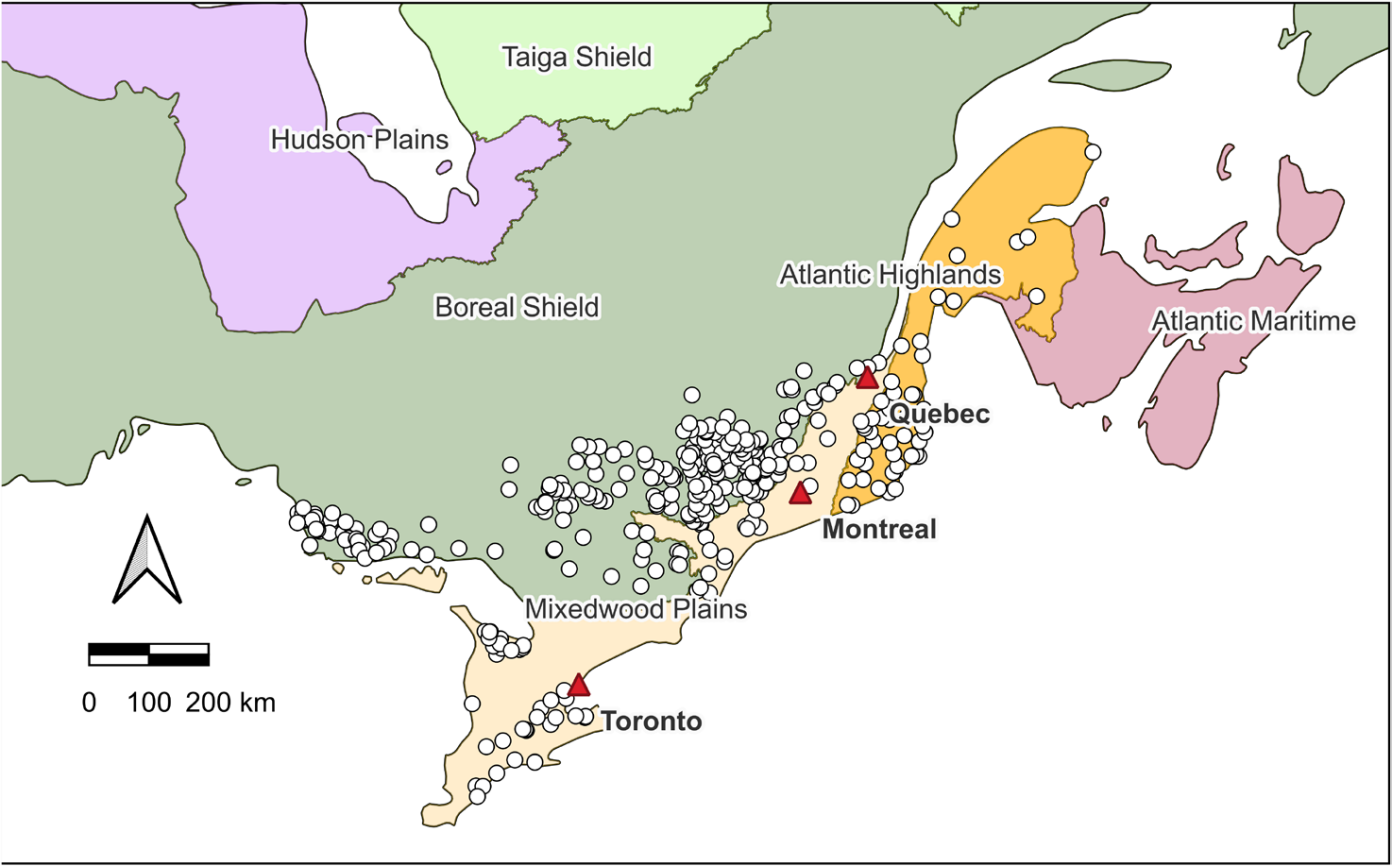
Approximate (± 10 km) geographic location of the 390 multi‐agency ground plots (MAGPlots) among the Atlantic Highlands, Mixedwood Plains, and Boreal Shield ecozones of eastern Canada. MAGPlots are shown as circles and main cities are shown as triangles.

The study's main goal was to describe long‐term spatial and temporal changes in sugar maple sapling abundance and identify the most important influential drivers using a composite dataset of ground plots located across Ontario, Québec, and New Brunswick, Canada. The composite dataset includes nearly 400 plots covering a wide geographical range of sugar maple forests (Figure 1), located in three distinct ecozones, including unharvested and harvested forests under a wide range of partial harvests. The first objective was to test if sugar maple sapling abundance declined over time, and the second objective was to determine the main drivers of sugar maple sapling abundance using explanatory variables that have biological meaning and were found to be influential in recent literature (e.g., Bose et al. 2017a, 2017b; Ducey et al. 2024; Stern et al. 2023; Leduc et al. 2024; Zarfos et al. 2024).

## 2 Methods

### 2.1 Study Plots and Ecozones

The Multi‐Agency Ground Plot (MAGPlot) database was used for this study. The MAGPlot database is a Canadian forest ground‐plot data repository. Different agencies, including the Federal National Forest Inventory (NFI) and 12 Canadian jurisdictions, provided forest ground plot datasets in their original format. For this study, MAGPlots were provided by the Governments of Ontario, Québec, New Brunswick, and the NFI (National Forest Inventory 2024). The composite database included 390 plots dominated by sugar maple (≥ 50% of total BA), measured 2–6 times between 1970 and 2022 (Table 1). Approximate plot locations (± 10 km) were made available. Plots were located within latitude 42.3 to 48.6°N, longitude −64.3 to −84.7°W, and elevation of 20 to 662 m. Plots were distributed among three different ecozones: Atlantic Highlands, Mixedwood Plains, and Boreal Shield (Table 1, Figure 1). Ecozones are defined as an area where organisms and their physical environment endure as a system (Wiken 1986). Broad combinations of characteristics like climate, landforms, and soils make them distinctive (Wiken 1986). The Atlantic Highlands used to be part of the larger Atlantic Maritime ecozone (Wiken 1986) but have been proposed as a distinct ecozone (Canadian Council on Ecological Classification 2017). It is located east of the Mixedwood Plains and Boreal Shield ecozones. Topography is dominated by the interior Appalachian Upland, covered by glacial till (Ecological Stratification Working Group 1996). The climate is characterized by warm summers and snowy, cold winters (Ecological Stratification Working Group 1996). The Mixedwood Plains ecozone is in the southeastern part of Canada. Underlain by carbonate‐rich Paleozoic bedrock, it is characterized by gentle topography, fertile soils, warm summers, and cool winters (Ecological Stratification Working Group 1996). The Boreal Shield ecozone is situated north of the Mixedwood Plains and study plots are in central Québec and Ontario, underlain by Precambrian granitic bedrock (Ecological Stratification Working Group 1996, Figure 1). Topography is dominated by a broadly rolling mosaic of uplands (Ecological Stratification Working Group 1996). Winters are longer and colder, while summers are shorter and cooler, although the Great Lakes have a moderating effect on the climate of Boreal Shield areas of central Ontario, warming them in winter and cooling them in summer (Ecological Stratification Working Group 1996).

**TABLE 1:** Number of plots by ecozone, data source, temporal range, measurement frequency (freq), and time interval (int) between measurements. When frequency or interval varied among plots, median values were provided.

Ecozone	Data source	Number of plots	Temporal range	Measurement frequency (freq), time interval (int) between measurements
Atlantic Highlands	QC	54	1970–2022	2–6×, median freq = 6, int. = 11 years
	NB	2	2016–2022	2×, every 5 years
	NFI	1	2003–2018	3×, every 5 or 10 years
	Total	57		
Mixedwood Plains	QC	7	1970–2022	3–6×, median freq = 5, int. = 11 years
	ON	48	1992–2001	2×, median int. = 5 years
	NFI	3	2004–2017	2×, median int. = 10 years
	Total	58		
Boreal shield	QC	199	1971–2022	2–6×, median freq = 5, int. = 11 years
	ON	74	1992–2012	2–3×, median freq = 2, int. = 5 years
	NFI	2	2004–2015	2×, median int. = 9 years
	Total	275		

Abbreviations: NB, Government of New Brunswick; NFI, Federal National Forest Inventory; ON, Government of Ontario; QC, Government of Québec.

At the time of the first measurement, plots were representative of temperate deciduous forests of the region, with a mean tree density of 525 trees ha^−1^ and a mean BA of 22.1 m^2^ ha^−1^ (Table 2). Species composition in the overstory (% total BA) was 77% sugar maple and 4% American beech (Table 2). Mean initial sapling density was 692 stems ha^−1^, with 61% sugar maple and 8% American beech (Table 2). Relative abundance of sugar maple saplings at the first measurement was 59% in the Mixedwood Plains, 60% in the Boreal Shield, and 69% in the Atlantic Highlands, but there was more variation in the Mixedwood Plains and Atlantic Highlands compared to the Boreal Shield (Table 2). At the plot level, relative abundance covered the entire range of possible values (0%–100%) for sugar maple and American beech saplings (Table 2).

**TABLE 2:** Mean (± standard error) tree and sapling density (stems ha^−1^), basal area (BA, m^2^ ha^−1^), and relative species composition (% BA) of sugar maple dominated plots at first measurement (initial measurement) by harvest treatment and ecozone.

Plot type and treatment	Number of sites	Density (stems ha^−1^)	BA (m^2^ ha^−1^)	Sugar maple (% BA)	Sugar maple range (% BA)	American beech (% BA)	American beech range (% BA)
Trees							
Partial cut	134	502 ± 17	21.6 ± 0.6	74.0 ± 1.4	50–100	6.3 ± 1.4	0–47
Uncut	256	544 ± 15	22.3 ± 0.5	78.0 ± 1.0	50–100	3.5 ± 0.5	0–48
Atlantic Highlands	57	614 ± 39	21.2 ± 1.3	78.2 ± 2.3	50–100	3.3 ± 1.0	0–31
Mixedwood Plains	58	502 ± 32	22.2 ± 0.9	75.6 ± 2.2	50–100	5.4 ± 1.4	0–48
Boreal Shield	275	518 ± 12	22.3 ± 0.5	76.5 ± 1.0	50–100	4.5 ± 0.6	0–47
All	390	530 ± 12	22.1 ± 0.4	76.6 ± 0.9	50–100	4.4 ± 0.8	0–48
Saplings							
Partial cut	134	630 ± 52	1.2 ± 0.1	56.1 ± 3.7	0–100	9.2 ± 3.7	0–100
Uncut	256	725 ± 64	1.3 ± 0.1	63.9 ± 2.4	0–100	8.0 ± 1.4	0–100
Atlantic Highlands	57	501 ± 41	1.0 ± 0.1	68.6 ± 5.6	0–100	4.4 ± 2.5	0–100
Mixedwood Plains	58	996 ± 159	1.9 ± 0.3	59.0 ± 4.4	0–100	12.7 ± 3.3	0–100
Boreal Shield	275	668 ± 41	1.2 ± 0.1	60.2 ± 2.5	0–100	8.4 ± 1.5	0–100
All	390	692 ± 46	1.3 ± 0.1	61.2 ± 2.0	0–100	8.4 ± 2.0	0–100

*Note:* The range of relative BA values is also shown for sugar maple and American beech.

### 2.2 Climate Variables

Climate variables were estimated using the BioSIM software (version 11, see Régnière et al. 2017). Approximate longitude and latitude coordinates of each plot were combined with climate normals provided in BioSIM to estimate daily values from 1980 to 2020. Daily values were averaged into months, and months were averaged into summer (June, July, and August) or annual values. The 40‐year average (1980–2020) was estimated for the following variables: mean air temperature, minimum air temperature, maximum air temperature, mean summer temperature, summer precipitation, total precipitation, and snow depth accumulation. Mean annual temperature and mean total annual precipitation for the 1980–2020 period were estimated at 4.2°C and1055 mm, respectively. Climatic conditions varied among plots, with mean annual temperature ranging from 1.3°C to 9.4°C and total annual precipitation ranging from 825 to 1525 mm.

### 2.3 Tree and Sapling Measurements

Tree and sapling data were collected through forest inventory programs of Ontario, Québec, New Brunswick, and the Federal NFI. In summary, fixed‐area ground plots were established as part of standard protocols to measure trees, defined as stems with > 9.0 cm in diameter at breast height (dbh) in this study. Tree plot size was 400 m^2^, except for 16 plots in Ontario that were 1000 m^2^. Tree status (living, living fallen, dead), species, height, and dbh were recorded. Tree condition was also noted, such as the presence and cause of tree injury, but information detail varied by data source. For example, as it pertains to beech bark disease (Evans et al. 2005), diseased trees were recorded in NFI plots, perennial target cankers were identified in Ontario plots, and trees affected by perennial *Nectria* spp. cankers were identified in Québec plots. Smaller fixed‐area plots (e.g., 25 or 50 m^2^) were established to measure saplings, i.e., stems with dbh > 1.0 cm and < 9.1 cm. Stand density and BA values in this study were scaled on a per‐hectare basis. An example of detailed field protocols can be found in National Forest Inventory (2008).

### 2.4 Silvicultural Treatments

Most silvicultural treatments were carried out operationally on public land, i.e., not in an experimental setting. Silvicultural treatments in temperate deciduous forests refer to periodical harvests of 15%–40% stand BA, carried out at regular intervals to reduce losses from mortality, improve stand quality, and maintain an uneven‐aged stand structure (Nyland 1996; Oliver and Larson 1996; Leak et al. 2014). Given that specific objectives and application detail information were limited, treatments were described using harvest intensity, i.e., the percent difference between preharvest and postharvest stand BA (preharvest—postharvest/postharvest BA). At least one follow‐up measurement was required to measure a treatment response (Table 1). Harvest intensities were also grouped into treatment class categories: Uncut (0%), low (5%–40%), and high (41%–80%) as per Bose et al. (2020). A fourth category (very high) was added for plots that were between 81% and 92% BA removal. The wide range of harvest intensities estimated reflects the small plot size (400 m^2^). For example, harvesting one large sugar maple tree (e.g., 50 cm in dbh) in a plot with a BA of 20 m^2^ ha^−1^ would lower the BA estimate by 5 m^2^ ha^−1^. This tree alone would represent a harvest intensity of 25%.

Documented partial cutting treatments were carried out in 134 plots between 1973 and 2012 (Table 3). A total of 111 partially harvested plots (83%) were in the Boreal Shield, 21 plots (16%) were in the Atlantic Highlands, and two plots were in the Mixedwood Plains. Harvest intensity averaged 53% (Table 3). Forty‐seven plots had low harvest intensity, 68 plots had high harvest intensity, and 19 plots had very high harvest intensity. Time since treatment ranged from 1 to 44 years, and mean time since treatment was 20 years. Postharvest measurement frequency and interval varied greatly among ecozones and treatment classes (Table 3). The specific management history of individual stands is not known, but sugar maple stands were most likely subjected to some form of partial harvest prior to the establishment of inventory programs (Majcen 1994). For the purposes of this study, plots with no documented treatments were considered uncut to allow comparison with partial cut plots.

**TABLE 3:** Descriptive statistics of partial harvesting treatments carried out operationally in sugar maple forests of eastern Canada by ecozone and treatment class.

Ecozone	Trt	Plots	Treatment year			Harvest intensity (%)			Time since treatment (years)		
			Min	Mean	Max	Min	Mean	Max	Min	Mean	Max
Atlantic	L	6	1978	1994	2008	24	31	38	1	15	44
Highlands	H	12	1986	1996	2008	45	60	74	1	16	32
	VH	3	2001	2007	2012	80	84	92	1	9	21
Mixedwood	L	1	2010	2010	2010	5	5	5	4	4	4
Plains	H	1	1994	1994	1994	58	58	58	15	22	28
	VH	0									
Boreal	L	40	1973	1993	2011	5	29	41	1	14	41
Shield	H	57	1977	1997	2013	41	60	80	1	13	39
	VH	16	1982	1996	2010	81	86	92	1	14	35

Abbreviations: H, high (41%–80%); L, low (5%–40%); Trt, treatment; and VH, very high (81%–92%) harvest intensity as defined by basal area removal.

### 2.5 Statistical Analyses

Study objectives were tested using linear mixed‐effects models. All fixed and random effects were specified prior to testing based on biological meaning and peer‐reviewed literature (Table 4). Each candidate model represented a biological hypothesis to explain variations in the response variable, i.e., the relative abundance of sugar maple saplings (% total sapling BA). The full model included all variables (Mazerolle 2006), including time (measurement year), while other candidate models had subsets of variables that focused on stand, quantitative and categorical harvest intensity, regional, or climatic conditions, alone or in combination (Table 5). Akaike's Information Criterion (Akaike 1973), Akaike weights (Burnham and Anderson 2002; Mazerolle 2006), and multimodel inference (Mazerolle 2006) were used to assess model fit and selection. Multimodel inference is recommended when no candidate models reach an Akaike weight ≥ 0.9 (Burnham and Anderson 2002). Marginal (fixed‐effects only) and conditional R^2^ (fixed and random effects) were calculated for each candidate model to estimate the amount of variation explained in the dependent variable. Plot identity was added as a random effect to account for the repeated‐measures nature of the data (Ducey et al. 2024). The initial measurement of the relative BA of sugar maple saplings was used as a covariate to account for potential preharvest (baseline) differences. Collinearity among potential explanatory variables was assessed using Pearson correlation. Preliminary analyses included several climate variables, i.e., mean, minimum, and maximum annual temperature, summer temperature, total and summer precipitation, as well as snow depth accumulation. Many of these climate variables were correlated with each other (see Appendix S1). Hence, mean summer temperature and precipitation were used in the models to resolve collinearity issues and better reflect growing season conditions (see Appendix S1). Pearson correlations were also used to associate relative abundance with absolute abundance in stand density or BA. Descriptive statistics were used to help explain the results. Homogeneity of variance and normality of residuals assumptions were met without data transformation. Given the large sample size, 95% confidence intervals with means were reported in all figures (Bose et al. 2020; Zarfos et al. 2024). The statistical programming language R was used to conduct all statistical analyses (version 4.4.2, R Core Team 2024). Source code and packages are included for all analyses in Appendix S1.

**TABLE 4:** List of variables used in candidate models.

Model variable	Group	Effect	Units	Range	Literature sources
1. Plot id	All	Random	Integer	1–390	Ducey et al. (2024)
2. Measurement year (temporal change)	All	Fixed	Year	1970–2022	Gauthier et al. (2015); Bose et al. (2020)
3. Relative BA of sugar maple trees (overstory)	Stand	Fixed	%	0–1	Bose et al. (2017a, 2017b)
4. Relative BA of American beech saplings	Stand	Fixed	%	0–1	Bose et al. (2017a); Zarfos et al. (2024)
5. Relative density of American beech trees with diseased stems or target cankers	Stand	Fixed	%	0–1	Zarfos et al. (2024)
6. Harvest intensity	Harvest	Fixed	%	0 for uncut, 0.05–0.92 for cut	Bose et al. (2020); Leduc et al. (2024)
7. Treatment class	Harvest	Fixed	Category	Uncut (0), low (0.05–0.40), high (0.41–0.80), Very high (0.81–0.92)	Bose et al. (2020)
8. Ecozone	Region	Fixed	Category	Atlantic Highlands, Mixedwood Plains, Boreal Shield	(Wiken 1986; Canadian Council on Ecological Classification 2017)
9. Mean summer temperature, 1980–2020	Climate	Fixed	°C	14.8–20.9	Stern et al. (2023)
10. Mean total summer precipitation, 1980–2020	Climate	Fixed	m	0.223–0.427	Stern et al. (2023)
11. Initial relative BA of sugar maple saplings	All	Covariate	%	0–1	Canham et al. (2006); Bose et al. (2023)

Abbreviation: BA, basal area.

**TABLE 5:** List of candidate models used to explain variation in the relative abundance of sugar maple saplings (% total BA).

Candidate model	AIC	Delta AIC	Akaike weight	Marginal *R* ^2^	Conditional *R* ^2^
1. Full model	758.9	22.1	< 0.01	0.36	0.53
2. Stand conditions model	736.8	0.0	0.63	0.34	0.52
3. Harvest intensity model	869.1	132.3	< 0.01	0.26	0.47
4. Regional conditions model	868.3	131.5	< 0.01	0.25	0.47
5. Climate model	853.1	116.3	< 0.01	0.26	0.47
6. Stand and harvest model	751.5	14.7	< 0.01	0.35	0.53
7. Stand and region model	746.7	10.0	< 0.01	0.34	0.52
8. Stand and climate model	737.8	1.1	0.37	0.35	0.53
9. Harvest and region model	881.6	144.8	< 0.01	0.26	0.47
10. Harvest and climate model	864.9	128.1	< 0.01	0.27	0.47
11. Region and climate model	853.2	116.4	< 0.01	0.27	0.47

Abbreviations: AIC, Akaike Information Criterion; *R* ^2^, regression coefficient for marginal (fixed effects only) or conditional (fixed and random effects) regressions.

## 3 Results

**FIGURE 2:**
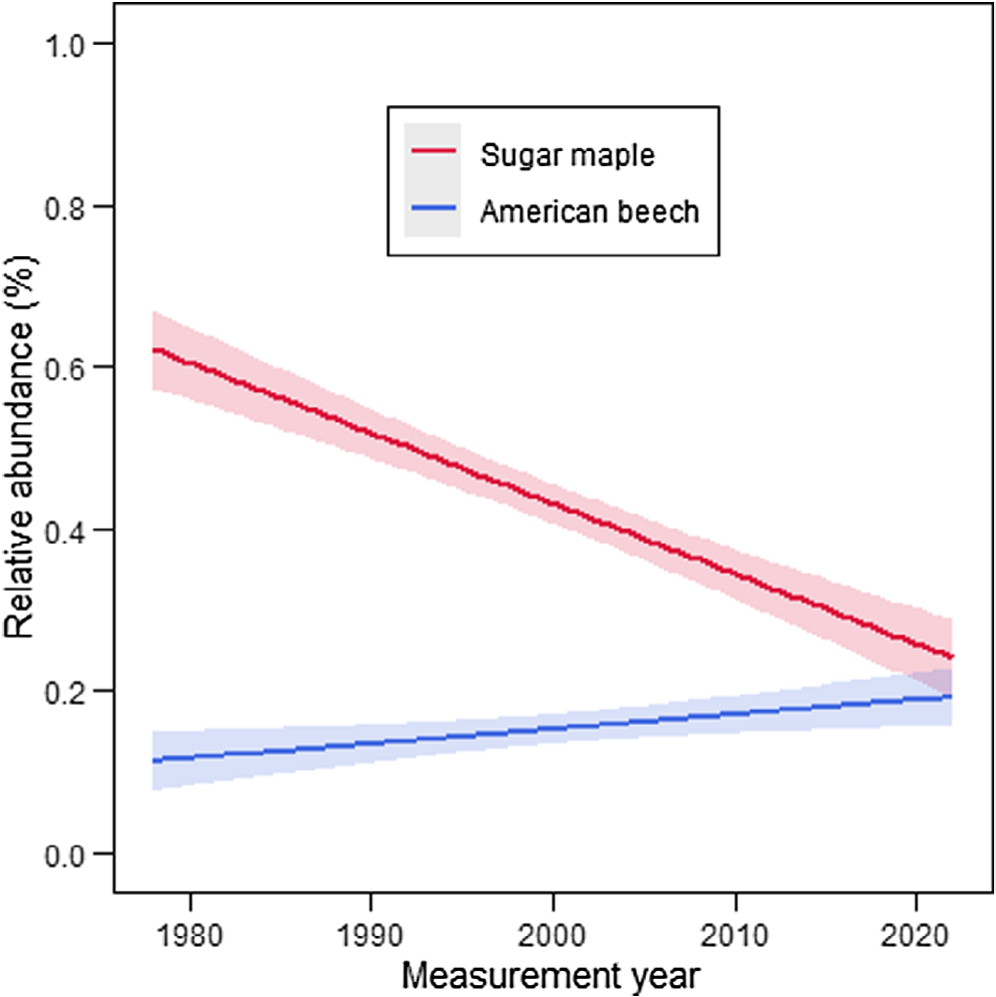
Change in relative abundance of sugar maple (above, red) and American beech (below, blue) saplings (% total sapling basal area, m^2^ ha^−1^) over time, from 1970 to 2022. Solid line shows the mean value of the linear fit and shaded areas represent the upper and lower confidence intervals at 95% based on observed data from all study plots and measurement years.

**FIGURE 3:**
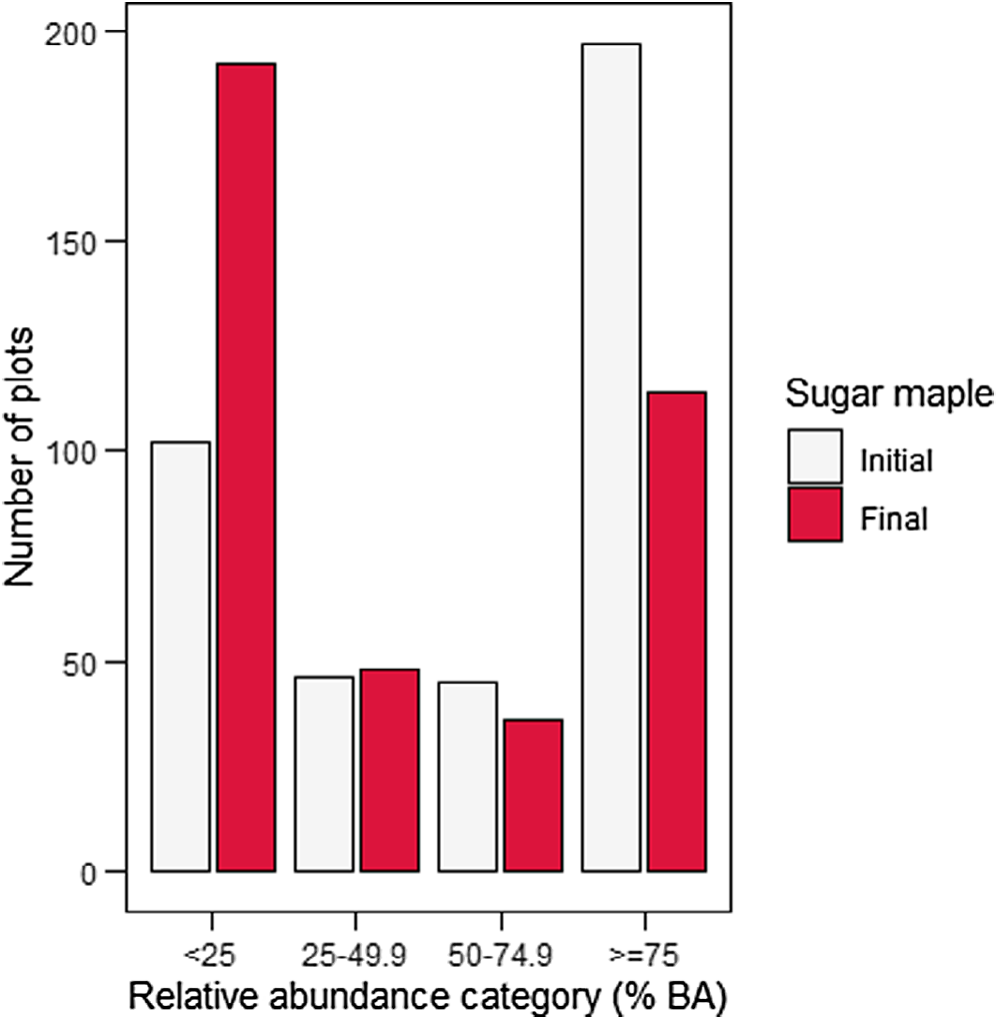
Plot count of the relative abundance of sugar maple saplings (% total sapling basal area, m^2^ ha^−1^) at initial and final measurements over time. Relative abundance categories are as follows: 0%–24.99%, 25%–49.99%, 50%–74.99%, and 75%–100%.

**FIGURE 4:**
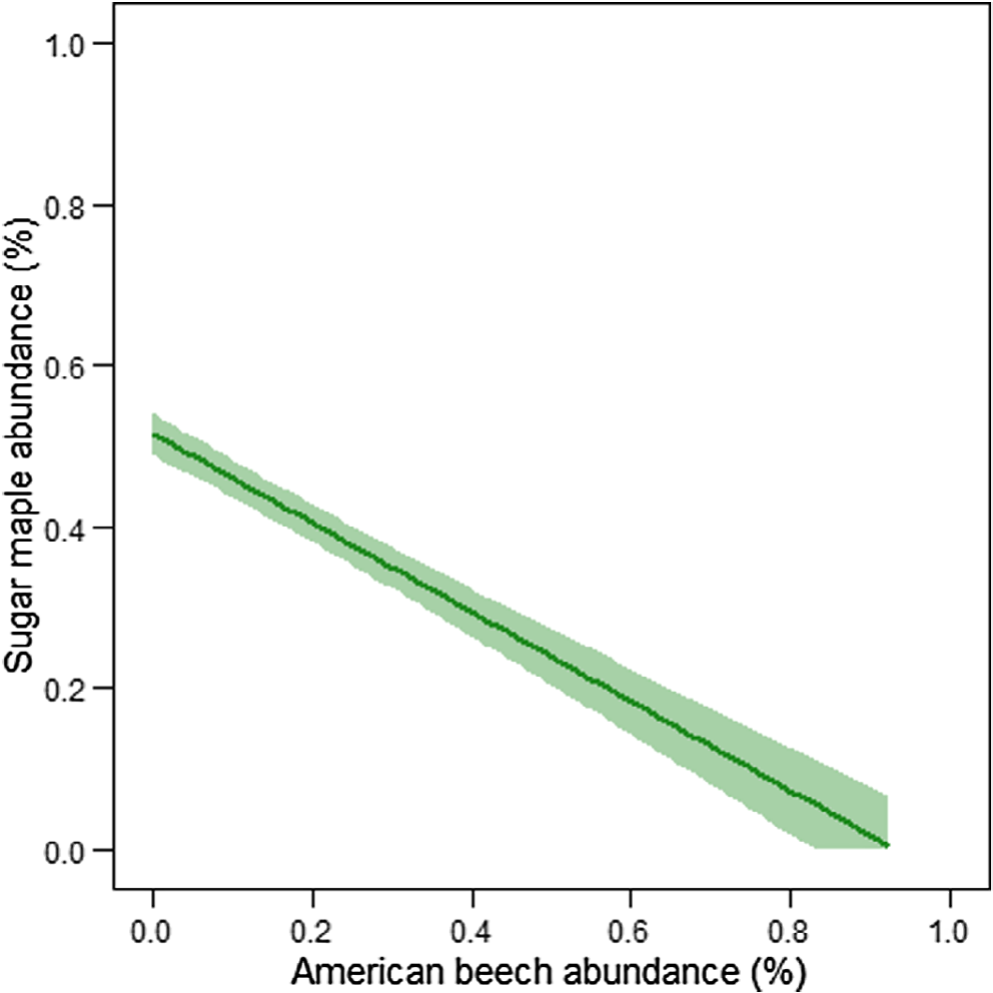
Influence of the relative abundance of American beech saplings (% total sapling basal area, m^2^ ha^−1^) on the relative abundance of sugar maple saplings (% total sapling basal area, m^2^ ha^−1^). Solid line shows the mean value of the linear fit and shaded areas represent the upper and lower confidence intervals at 95% based on observed data from all plots and measurement years.

**FIGURE 5:**
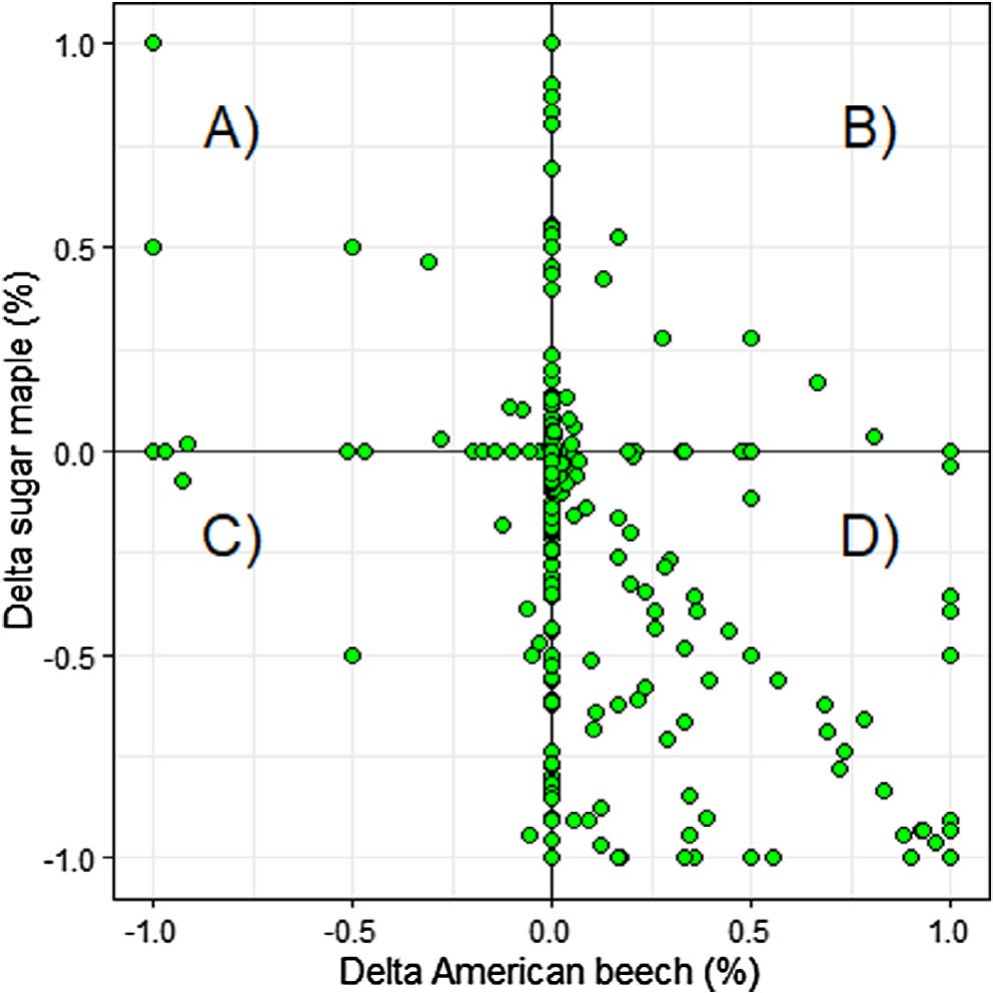
Plot‐specific distribution of the change in the relative abundance (% total sapling basal area) of sugar maple and American beech saplings (Delta = relative abundance at final measurement—relative abundance at initial measurement). Plots in quadrant (A) had an increase in sugar maple and a decrease in beech, plots in quadrant (B) showed an increase in maple and an increase in beech, plots in quadrant (C) had a decrease in maple and a decrease in beech, while plots in quadrant (D) had a decrease in maple and an increase in beech. Plots located at the intersection of all quadrats (0, 0) had no change between final and initial measurement.

Candidate models had AIC values ranging from 736.8 to 881.6 (Table 5). The stand conditions model had the lowest AIC (736.8), the lowest delta AIC (0), and the highest Akaike weight (0.63), indicating it had a 63% probability of having the best fit. Its marginal *R* ^2^ was 0.34 and conditional *R* ^2^ was 0.52. The stand conditions and climate model came in second with an Akaike weight of 0.37 and *R* ^2^ values that were comparable to the stand conditions model. The Akaike weight of other models was very low (Table 5). Multimodel inference confirmed that only variables from the stand conditions model were significant (*p* < 0.05): measurement year, relative abundance of American beech saplings, and the covariate (Table 6). No significant differences were found in harvest intensity, treatment class, region (ecozone), and climate variables (Table 6). The measurement year effect showed that sugar maple sapling abundance declined significantly between 1970 and 2022, as reflected in the observed data (Table 6, Figure 2). At the initial measurement, many plots had at least 75% of their total sapling BA as sugar maple (Figure 3). At the final measurement, however, many plots had less than 25% of their total sapling BA as sugar maple (Figure 3). Decreases in sugar maple sapling abundance were found with increases in the relative abundance of American beech saplings (Table 6, Figure 4). The plot‐specific distribution of change between the final and initial measurements over time revealed that many plots showing a decline in relative sugar maple sapling abundance also experienced an increase in relative American beech sapling abundance (Figure 5, quadrant D). The covariate effect accounted for potential baseline differences (Table 6).

**TABLE 6:** Model‐averaged coefficients from multimodel inference of relative abundance of sugar maple saplings.

Variable	Estimate	SE	*z*	*p*
Intercept	16.99	1.576	10.764	< 0.001
Measurement year	−0.008	0.001	10.846	< 0.001
Relative BA of sugar maple trees	−0.014	0.054	0.255	0.799
Relative BA of American beech saplings	−0.450	0.038	11.873	< 0.001
Relative density of American beech trees with diseased stems/target cankers	0.262	0.193	1.357	0.175
Covariate (initial sugar maple sapling abundance)	0.365	0.035	10.686	< 0.001
Mean summer temperature	−0.003	0.009	0.370	0.711
Mean summer precipitation	−0.375	0.537	0.698	0.485
Ecozone—Atlantic Highlands	Reference	—	—	—
Ecozone—Mixedwood Plains	0.004	0.007	0.056	0.955
Ecozone—Boreal Shield	< 0.001	0.003	0.034	0.973
Harvest intensity	< 0.001	0.009	0.017	0.986
Treatment class—Uncut	Reference	—	—	—
Treatment class—High	< 0.001	0.005	0.017	0.986
Treatment class—Low	< 0.001	0.002	0.010	0.992
Treatment class—Very high	< 0.001	0.007	0.017	0.986

Abbreviations: BA, basal area; SE, standard error of the mean.

The relative abundance of sugar maple saplings was positively correlated with the absolute density (*r* = 0.54) and BA (*r* = 0.58) of sugar maple saplings (Table 7) and negatively correlated with the absolute density (*r* = −0.35) and BA (*r* = −0.33) of American beech saplings (Table 7). The relative abundance of American beech saplings was positively correlated with the absolute density (*r* = 0.76) and BA (*r* = 0.76) of beech saplings (Table 7). Correlations between both species and total density or total BA were low or non‐significant.

**TABLE 7:** Pearson correlation coefficients (*r*) and corresponding *p*‐values between relative and the absolute abundance of sugar maple and American beech saplings.

Variable	Relative maple BA (*r* and *p*)	Relative beech BA (*r* and *p*)
Relative maple abundance (% total sapling BA)	NA	
Relative beech abundance (% total sapling BA)	−0.40, *p* < 0.001	NA
Maple sapling BA (m^2^ ha^−1^)	0.58, *p* < 0.001	−0.25, *p* < 0.001
Beech sapling BA (m^2^ ha^−1^)	−0.33, *p* < 0.001	0.76, *p* < 0.001
Maple sapling density (stems ha^−1^)	0.54, *p* < 0.001	−0.24, *p* < 0.001
Beech sapling density (stems ha^−1^)	−0.35, *p* < 0.001	0.76, *p* < 0.001
Total sapling BA (m^2^ ha^−1^)	NS, *p* = 0.093	NS, *p* = 0.873
Total sapling density (stems ha^−1^)	−0.13, *p* < 0.001	NS, *p* = 0.276

Abbreviations: BA, basal area; NA, not applicable; NS, not significant (*p* > 0.05).

## 4 Discussion

### 4.1 Sugar Maple Decline Over Time and Influence of Stand Conditions

Results showed that the relative abundance of sugar maple saplings declined in sugar maple forests over the past six decades in eastern Canada, and the relative abundance was correlated with absolute measures of sapling abundance. Relative abundance of American beech saplings was an important explanatory variable of sugar maple decline. The negative quantitative effect of beech sapling abundance on sugar maple sapling abundance has not been reported in long‐term, large‐scale studies because species or species groups are often analyzed separately (e.g., Bose et al. 2017a, 2017b; Ducey et al. 2024; Zarfos et al. 2024). Hence, results from this study complement smaller scale, mechanistic studies that demonstrate American beech is a better competitor for light, water, and nutrients compared to sugar maple. Beech has greater shade tolerance (Hane 2003); its asexual reproduction ability provides saplings with an interconnected root system, increasing survival (Jones and Raynal 1986; Nyland et al. 2006). Moreover, beech is not preferred deer food (Tripler et al. 2005), and its growth is more tolerant of soil acidity and pollution (Stern et al. 2023; Zarfos et al. 2024). It should be noted, however, that many of the study plots that showed a decline in the relative abundance of sugar maple saplings showed no change in the relative abundance of beech. Given the low relative abundance of beech at first measurement (8% on average), other variables not tested in this study likely had a negative impact on sugar maple regeneration, particularly at the beginning of the temporal scale. One of these variables may be related to the history of long‐term atmospheric acid deposition that lowered site fertility by depleting soil base cations in temperate deciduous forests of northeastern North America (Sullivan et al. 2013; Cleavitt et al. 2018). Soils of the southeastern portion of the Boreal Shield ecozone, where most of the study plots were located, are known to be acidic, and base cation deficiencies were correlated with lower sugar maple sapling abundance (Gauthier et al. 2015). Unfortunately, the effect of soil base cation deficiencies could not be tested in this study due to lack of comprehensive soil data. The covariate effect accounted for the influence of potential baseline differences as shown by earlier studies in temperate deciduous forests (Canham et al. 2006; Bose et al. 2020).

Not all variables related to stand conditions influenced sugar maple sapling abundance. No relationship was found between the abundance of sugar maple trees in the overstory and increased sugar maple saplings in the understory. This was unexpected, because sugar maple trees act as the seed source for regeneration. Gauthier et al. (2015) and Bose et al. (2017a) both reported statistically significant relationships between the absolute density of sugar maple overstory BA and sugar maple sapling density. Perhaps selecting plots where sugar maple trees accounted for at least 50% of the initial BA was too narrow and limited the potential to identify a significant relationship. Likewise, the amount of diseased American beech stems did not appear to influence sugar maple saplings. This variable was used as a proxy to estimate the amount of beech bark disease in the stand. Zarfos et al. (2024) found that beech sapling density was unrelated to the overall proportion of overstory beech or beech bark disease severity. Overstory beech crown decline and mortality release beech seedlings and sprouts through increased light availability (Flinn et al. 2022), not the presence of the disease on its host (Roy and Nolet 2018). The dbh and relative growth rate of beech trees could be used to evaluate beech bark disease severity based on a recent study carried out in Ontario (Kish et al. 2022).

### 4.2 Harvest Intensity

No relationship was found between harvest intensity and sugar maple sapling abundance. Some studies have reported similar findings with low‐intensity harvesting (Fraver et al. 2009; Kern et al. 2017) while others have reported increases in sapling growth and mortality after high‐intensity harvesting (Bose et al. 2020). MAGPlot data used to estimate harvest intensity for this study was at the plot level and did not include the size of harvest operations and treatment objectives. It is likely that the wide range of intensities estimated (5%–92%) reflects the small plot size (400 m^2^). Larger plots would have provided a more accurate estimate of BA removal at the stand level. Nevertheless, the lack of differences between harvested and unharvested plots suggests understory treatments like mechanical removal may be required to control beech saplings, and soil treatments such as liming may be required to promote sugar maple regeneration and development. This echoes recent findings from Bose et al. (2023) and Leduc et al. (2024), with the latter reporting that adjusting the harvest intensity (0%–80%) alone cannot favor the growth of sugar maple regeneration where American beech saplings dominate the understory. Other findings from the literature suggest that partial harvests of diseased beech overstory can help maintain advance regeneration of sugar maple in the understory, but do not control or mitigate the proliferation of beech asexual reproduction, such as stump sprouts and root suckers (Dracup and MacLean 2017). Modifying light availability alone does not appear to promote sugar maple regeneration over beech regeneration either (Bannon et al. 2015). Mechanical removal of beech regeneration with brush saws alone has not proven effective (Nolet et al. 2015). Liming was shown to increase growth and development of sugar maple in soils with low to medium fertility (Ouimet et al. 2017; Bognounou et al. 2023). Concerns with liming are related to the risk of long‐term earthworm invasions and subsequent negative impacts on ecological processes (Moore et al. 2015). Herbicide application to eliminate asexual reproduction of beech has proved effective (Kochenderfer et al. 2013), but social acceptance is low, and herbicide use is not permitted in some jurisdictions. Preferential browsing by deer may reduce the efficiency of herbicide application (Bose et al. 2018). Soil scarification has shown a limited effect on seedling establishment of sugar maple (Bognounou et al. 2023). Soil scarification has been effective in regenerating associated tree species like yellow birch ( *Betula alleghaniensis* Britt.) and white birch ( *Betula papyrifera* Marsh.) in some studies (Leak 1999; Gauthier et al. 2016; Bognounou et al. 2023), but not in others (Bolton and D'Amato 2011; Kern et al. 2013; Poznanovic et al. 2013; D'Amato et al. 2015).

### 4.3 Climate and Regional Conditions

Despite the wide range of summer temperature and precipitation gradients among study plots, no relationship was found with sugar maple sapling abundance. Increases in temperature have been associated with both increases (Zarfos et al. 2024) and reductions (Bose et al. 2017a, 2017b) in sugar maple sapling abundance, while some studies did not find any relationship between maple sapling abundance and climate (Gauthier et al. 2015). Growth reductions were reported with increases in vapor pressure deficits (Boakye et al. 2023), as well as frequency and extent of drought events (Moreau et al. 2020). Growth increases were linked to increased moisture during the summer and increased precipitation during winter (Stern et al. 2023). Climate variables better reflect differences in sugar maple growth compared to differences in abundance. There does not appear to be a consensus on climate variables that drive sugar maple sapling abundance. Relating climate variables with temporal changes in abundance is challenging because tree and sapling mortality are difficult to estimate in temperate deciduous forests due to their stochastic nature and rare occurrence (e.g., Guillemette et al. 2017). Likewise, despite variations in climate and topography, ecozones did not help explain variation in sugar maple sapling abundance. There was an unbalanced number of plots per ecozone and not all harvest intensities were present in each ecozone. Greater variation among plots was found in the Atlantic Highlands and Mixedwood Plains compared to the Boreal Shield. The ecozone scale may be too large to capture regional conditions, and using a smaller scale like ecoregions may be preferable.

Overall, results indicate (1) relative sugar maple sapling abundance declined substantially over time, (2) harvest intensity had no effect on sugar maple sapling abundance, and (3) increases in beech sapling abundance helped to explain the decline in sugar maple. Combining partial harvests with understory mechanical removal of beech saplings may help reduce competition. Soil liming treatments to promote sugar maple regeneration may be beneficial as well.

## Author Contributions

**Martin‐Michel Gauthier:** conceptualization (lead), formal analysis (lead), investigation (lead), methodology (lead), writing – original draft (lead), writing – review and editing (lead).

## Conflicts of Interest

The author declares no conflicts of interest.

## Supporting information


**Table S1.** List of predictor variable abbreviations, definitions, range, and units.
**Table S2.** Preliminary Peason correlation coefficients (*r*). Strong correlations shown in yellow.
**Table S3.** Final Pearson correlation coefficients (*r*).

## Data Availability

The data that support the findings of this study are available from Canada's National Forest Inventory (NFI) and the MAGPlot Working Group, a collaborative initiative of Canadian federal, provincial, and territorial government agencies, as well as other data contributors. Restrictions apply to the availability of these data, which were used under license for this study. Data are available on request: https://open.canada.ca/data/en/dataset/8824392d‐464e‐413d‐8bde‐eaed61c79743 with the permission of NFI and the MAGPlot Working Group.
