# Supplementary material for: Six Decades of Forest Inventory Data Highlight Decline of Sugar Maple (Acer saccharum) Sapling Abundance in Eastern Canada
Source: Ecol Evol. 2025 May 15;15(5):e71386. doi: 10.1002/ece3.71386 (PMC12081324; doi:10.1002/ece3.71386)
Supplement: Supplementary file 1 — Table S1. List of predictor variable abbreviations, definitions, range, and units. Table S2. Preliminary Peason correlation coefficients (r). Strong correlations shown in yellow. Table S3. Final Pearson correlation coefficients (r). [file ECE3-15-e71386-s001.docx]

Supplementary Material S1. Results from paired Pearson correlations (r) of predictor variables to assess potential collinearity prior to linear mixed-effects models.

Table S1. List of predictor variable abbreviations, definitions, range, and units.

| Value | Definition and units |
| --- | --- |
| Yr | Measurement year, 1970–2022 |
| Cov | Relative abundance of sugar maple at first measurement, sapling basal area, 0–1% |
| Over | Relative abundance of sugar maple in the overstory, tree basal area, 0–1% |
| Beech | Relative abundance of American beech, sapling basal area, 0–1% |
| Harv | Harvesting intensity, percent basal area removal, 0–0.92% |
| Tmean | Mean annual temperature, 1980–2020, 1.3–9.4 °C |
| Tmin | Minimum annual temperature, 1980–2020, -5.0–5.4 °C |
| Tmax | Maximum annual temperature, 1980–2020, 7.5–13.6 °C |
| Tsum | Mean summer temperature, 1980–2020, 14.8–20.9 °C |
| Psum | Mean total summer precipitation, 1980–2020, 0.22–0.43 m |
| Ptot | Mean total annual precipitation, 1980–2020, 0.82–1.53 m |
| Snow | Mean annual snow depth accumulation, 1980–2020, 0.5–4.5 m |
| Dis | Percent density of American beech with presence of diseased stems or perennial target cankers of *Nectria* spp., 0–1% |

Table S2. Preliminary Peason correlation coefficients (*r*). Strong correlations shown in yellow.

| Var | Yr | Cov | Over | Beech | Harv | Tmean | Tmin | Tmax | Tsum | Psum | Ptot | Snow | Dis |
| --- | --- | --- | --- | --- | --- | --- | --- | --- | --- | --- | --- | --- | --- |
| Yr | 1 |  |  |  |  |  |  |  |  |  |  |  |  |
| Cov | 0.01 | 1 |  |  |  |  |  |  |  |  |  |  |  |
| Over | -0.20 | 0.29 | 1 |  |  |  |  |  |  |  |  |  |  |
| Beech | 0.08 | -0.19 | -0.18 | 1 |  |  |  |  |  |  |  |  |  |
| Harv | 0.04 | -0.10 | -0.11 | 0.09 | 1 |  |  |  |  |  |  |  |  |
| Tmean | -0.03 | -0.05 | -0.03 | 0.06 | -0.28 | 1 |  |  |  |  |  |  |  |
| Tmin | -0.04 | 0.01 | -0.01 | 0.03 | -0.27 | 0.97 | 1 |  |  |  |  |  |  |
| Tmax | -0.01 | -0.13 | -0.06 | 0.09 | -0.25 | 0.90 | 0.76 | 1 |  |  |  |  |  |
| Tsum | -0.04 | -0.10 | -0.08 | 0.09 | -0.26 | 0.94 | 0.88 | 0.90 | 1 |  |  |  |  |
| Psum | 0.01 | 0.10 | -0.01 | 0.03 | 0.01 | -0.09 | 0.05 | -0.30 | -0.18 | 1 |  |  |  |
| Ptot | 0.01 | 0.10 | 0.01 | 0.01 | -0.06 | 0.04 | 0.18 | -0.20 | -0.08 | 0.92 | 1 |  |  |
| Snow | 0.04 | 0.09 | 0.09 | -0.09 | 0.09 | -0.71 | -0.60 | -0.78 | -0.71 | 0.26 | 0.36 | 1 |  |
| Dis | 0.16 | 0.00 | -0.18 | 0.16 | -0.03 | 0.09 | 0.08 | 0.08 | 0.08 | 0.07 | 0.05 | -0.08 | 1 |

Table S3. Final Pearson correlation coefficients (*r*).

| Var | Yr | Cov | Over | Beech | Harv | Tsum | Psum | Dis |
| --- | --- | --- | --- | --- | --- | --- | --- | --- |
| Yr | 1 |  |  |  |  |  |  |  |
| Cov | 0.01 | 1 |  |  |  |  |  |  |
| Over | -0.20 | 0.29 | 1 |  |  |  |  |  |
| Beech | 0.08 | -0.19 | -0.18 | 1 |  |  |  |  |
| Harv | 0.04 | -0.10 | -0.11 | 0.09 | 1 |  |  |  |
| Tsum | -0.04 | -0.10 | -0.08 | 0.09 | -0.26 | 1 |  |  |
| Psum | 0.01 | 0.10 | -0.01 | 0.03 | 0.01 | -0.18 | 1 |  |
| Dis | 0.16 | 0.01 | -0.18 | 0.16 | -0.03 | 0.08 | 0.07 | 1 |

Source code for R programming language

FILE 1) COMPILE DATA QUÉBEC

#################################################

### COMPILE THE COMPOSITE DATASET ##########

### REFER TO DATA DICTIONARY FOR TERMS ##########

#################################################

### 1. IMPORT QUEBEC MAGPLOT DATA

library(data.table)

library(dplyr)

library(sf)

library(tidyr)

library(ggplot2)

library(knitr)

library(broom)

# Import tree data - Replace with the path to your own CSV file

data <- fread("C:/Users/martigau/OneDrive - NRCan RNCan/Documents/Personnel/Travail/EcolEvol/NFI Data/QC_PSP/magp_trees.csv")

# Data header

data_header <- fread("C:/Users/martigau/OneDrive - NRCan RNCan/Documents/Personnel/Travail/EcolEvol/NFI Data/QC_PSP/magp_tree_header.csv")

# Ensure numeric columns are treated as numeric

data$dbh <- as.numeric(data$dbh)

data_header$meas_plot_size <- as.numeric(data_header$meas_plot_size)

data_header$meas_year <- as.numeric(data_header$meas_year)

#Data on plot site

unique_plot_sizes <- data_header %>%

group_by(plot_type_id) %>%

summarise(unique_meas_plot_size = unique(meas_plot_size))

# View the result

print(unique_plot_sizes)

# Merge based on magp_site_id, plot_type_id, meas_num

merged_data <- merge(data, data_header, by = c("magp_site_id", "plot_type_id", "meas_num"))

# Specify the file path where you want to save the CSV file

# This will take some time

file_path <- "C:/Users/martigau/OneDrive - NRCan RNCan/Documents/Personnel/Travail/EcolEvol/NFI Data/QC_PSP/merged_data.csv"

write.csv(merged_data, file = file_path)

# Calculate basal area (m^2/ha)

merged_data$ba_ha <- (pi * (merged_data$dbh / 200)^2) / merged_data$meas_plot_size

write.csv(merged_data, file = file_path)

# Keep only the living trees and saplings (exclude mortality)

relevant_rows <- merged_data[merged_data$tree_status %in% c('LF', 'LS', 'L', '-1'), ]

file_path <- "C:/Users/martigau/OneDrive - NRCan RNCan/Documents/Personnel/Travail/EcolEvol/NFI Data/QC_PSP/relevant_rows.csv"

write.csv(relevant_rows, file = file_path)

# CALCULATE BASAL AREA FOR EACH TREE BY SITE, PLOT, MEAS_NUM, and SPECIES

library(dplyr)

# Sum basal area by experimental unit

ba_sum <- relevant_rows %>%

group_by(magp_site_id, plot_type_id, meas_num, species_gs, meas_year) %>%

summarize(total_basal_area_m2 = sum(ba_ha),

total_stems_per_ha = sum(stem_ha))

colnames(ba_sum)

# Keep specific columns in summary_data

summary_data_filtered <- ba_sum %>%

select(magp_site_id, plot_type_id, meas_num, meas_year, species_gs, total_stems_per_ha, total_basal_area_m2)

# View the filtered summary data

print(summary_data_filtered)

# Export file along with French version (_fr)

file_path <- "C:/Users/martigau/OneDrive - NRCan RNCan/Documents/Personnel/Travail/EcolEvol/NFI Data/QC_PSP/summary_data_filtered.csv"

write.csv(summary_data_filtered, file = file_path)

write.csv2(summary_data_filtered, "summary_data_fr.csv", row.names = FALSE)

# Merge with 'magp_sites' file to have latitude, longitude, and elevation

data_sites <- fread("C:/Users/martigau/OneDrive - NRCan RNCan/Documents/Personnel/Travail/EcolEvol/NFI Data/QC_PSP/magp_sites.csv")

merged_data_sites <- merge(summary_data_filtered, data_sites, by = c("magp_site_id"), all.x = TRUE)

print(merged_data_sites)

# Merge with 'magp_treatment' file to have silvicultural treatments

data_treat <- fread("C:/Users/martigau/OneDrive - NRCan RNCan/Documents/Personnel/Travail/EcolEvol/NFI Data/QC_PSP/magp_treatment.csv")

data_treat2 <- data_treat %>%

select(magp_site_id, treatment_type, treatment_year, treatment_month, treatment_pct)

# Left join to keep 'control' plots

merged_site_treat <- merge(merged_data_sites, data_treat2, by = c("magp_site_id"), all.x = TRUE)

# Replace NA values in treatment_type with "Control"

merged_site_treat$treatment_type[is.na(merged_site_treat$treatment_type)] <- "CON"

# Check the resulting data

head(merged_site_treat)

# Specify the file path where you want to save the CSV file

file_path <- "C:/Users/martigau/OneDrive - NRCan RNCan/Documents/Personnel/Travail/EcolEvol/NFI Data/QC_PSP/merged_site_treat.csv"

write.csv(merged_site_treat, file = file_path)

write.csv2(merged_site_treat, "merged_site_treat_fr.csv", row.names = FALSE)

#### PIVOT TABLE - PUT SPECIES IN COLUMN FORMAT

#Pivot the data from long format to wide format, where species are columns

library(dplyr)

library(tidyr)

wide_df <- merged_site_treat %>%

pivot_wider(names_from = species_gs,

values_from = c(total_stems_per_ha, total_basal_area_m2))

# Replace NA with 0

wide_df2 <- wide_df %>%

mutate(across(starts_with("total_stems_per_ha_"), ~replace(., is.na(.), 0)),

across(starts_with("total_basal_area_m2_"), ~replace(., is.na(.), 0)))

# Print the result

print(wide_df2)

# Export file

file_path <- "C:/Users/martigau/OneDrive - NRCan RNCan/Documents/Personnel/Travail/EcolEvol/NFI Data/QC_PSP/wide_df2.csv"

write.csv(wide_df2, file = file_path)

write.csv2(wide_df2, "pivot_table_species_fr.csv", row.names = FALSE)

# IDENTIFY UNIQUE SPECIES LIST FOR QUEBEC, n = 64

unique_species_qc <- unique(relevant_rows$species_gs)

# Print the unique species

print(unique_species_qc)

#Save as .csv file

file_path <- "C:/Users/martigau/OneDrive - NRCan RNCan/Documents/Personnel/Travail/EcolEvol/NFI Data/QC_PSP/unique_species_qc.csv"

write.csv(unique_species_qc, file = file_path)

# ADD DENSITY AND BASAL AREA TOTALS FOR QUEBEC SPECIES

library(dplyr)

library(tidyr)

# List of species from unique list

species_list <- c(

"ACER.SAH", "ACER.RUB", "FAGU.GRA", "FRAX.AME", "OSTR.VIR",

"ULMU.AME", "BETU.ALL", "BETU.PAP", "ABIE.BAL", "ACER.PEN",

"QUER.RUB", "TILI.AME", "TSUG.CAN", "PRUN.SER", "AMEL.SPP",

"UNKN.SPP", "PICE.RUB", "PICE.MAR", "PICE.GLA", "PRUN.PEN",

"THUJ.OCC", "SORB.AME", "ACER.SPI", "SORB.DEC", "POPU.TRE",

"POPU.BAL", "SALI.SPP", "PINU.STR", "ALNU.RUG", "LARI.LAR",

"CRAT.SPP", "FRAX.NIG", "QUER.MAC", "POPU.GRA", "PINU.BAN",

"BETU.POP", "ACER.SAC", "FRAX.PEN", "CARY.COR", "CORN.ALT",

"ULMU.RUB", "JUGL.CIN", "PINU.RES", "ALNU.VIR", "RHAM.SPP",

"PICE.ABI", "PRUN.VIR", "ACER.NIG", "POPU.DEL", "MALU.SPP",

"ULMU.THO", "POPU.SPP", "QUER.ALB", "CARP.CAR", "QUER.BIC",

"HAMA.VIR", "JUNI.VIR", "ACER.NEG", "RHUS.TYP", "PINU.SYL",

"LARI.MAR", "CARY.OVA", "PINU.RIG", "CELT.OCC"

)

# Generate column names for the species in the list

stem_columns <- paste0("total_stems_per_ha_", species_list)

basal_area_columns <- paste0("total_basal_area_m2_", species_list)

# Ensure the columns exist in the dataframe

stem_columns <- intersect(stem_columns, names(wide_df2))

basal_area_columns <- intersect(basal_area_columns, names(wide_df2))

# Perform the calculations

wide_df3 <- wide_df2 %>%

rowwise() %>%

mutate(

total_density = sum(c_across(all_of(stem_columns)), na.rm = TRUE),

total_basal_area = sum(c_across(all_of(basal_area_columns)), na.rm = TRUE)

) %>%

ungroup() %>%

mutate(

# Create percentage columns for density

across(

starts_with("total_stems_per_ha_"),

~ . / (total_density + 1e-6) * 100,

.names = "pct_density_{col}"

),

# Create percentage columns for basal area

across(

starts_with("total_basal_area_m2_"),

~ . / (total_basal_area + 1e-6) * 100,

.names = "pct_basal_area_{col}"

)

)

# Extract only the percentage columns

percentage_columns <- names(wide_df3) %>%

.[grepl("pct_density_|pct_basal_area_", .)]

# Create a final data frame with just the percentage columns and any other necessary columns

wide_df3_final <- wide_df3 %>%

select(all_of(percentage_columns))

# Save the modified data frame to CSV

write.csv(wide_df3_final, file = "C:/Users/martigau/OneDrive - NRCan RNCan/Documents/Personnel/Travail/EcolEvol/NFI Data/QC_PSP/wide_df3.csv", row.names = FALSE)

write.csv2(wide_df3_final, file = "C:/Users/martigau/OneDrive - NRCan RNCan/Documents/Personnel/Travail/EcolEvol/NFI Data/QC_PSP/wide_df3_fr.csv", row.names = FALSE)

# Calculate total density and total basal area for each plot

# Calculate the percentage of sugar maple, American beech, and yellow birch

wide_df3 <- wide_df2 %>%

mutate(total_density = total_stems_per_ha_ACER.RUB + total_stems_per_ha_ACER.SAH +

total_stems_per_ha_FAGU.GRA + total_stems_per_ha_FRAX.AME + total_stems_per_ha_ULMU.AME +

total_stems_per_ha_OSTR.VIR + total_stems_per_ha_BETU.ALL + total_stems_per_ha_BETU.PAP +

total_stems_per_ha_ABIE.BAL + total_stems_per_ha_ACER.PEN + total_stems_per_ha_QUER.RUB +

total_stems_per_ha_TILI.AME + total_stems_per_ha_TSUG.CAN + total_stems_per_ha_PRUN.SER +

total_stems_per_ha_AMEL.SPP + total_stems_per_ha_UNKN.SPP + total_stems_per_ha_PICE.RUB +

total_stems_per_ha_PRUN.PEN + total_stems_per_ha_PICE.GLA + total_stems_per_ha_PICE.MAR +

total_stems_per_ha_THUJ.OCC + total_stems_per_ha_SORB.AME + total_stems_per_ha_ACER.SPI +

total_stems_per_ha_SORB.DEC + total_stems_per_ha_POPU.BAL + total_stems_per_ha_POPU.TRE +

total_stems_per_ha_SALI.SPP + total_stems_per_ha_PINU.STR + total_stems_per_ha_ALNU.RUG +

total_stems_per_ha_LARI.LAR + total_stems_per_ha_CRAT.SPP + total_stems_per_ha_FRAX.NIG +

total_stems_per_ha_QUER.MAC + total_stems_per_ha_POPU.GRA + total_stems_per_ha_PINU.BAN +

total_stems_per_ha_BETU.POP + total_stems_per_ha_ACER.SAC + total_stems_per_ha_FRAX.PEN +

total_stems_per_ha_CARY.COR + total_stems_per_ha_CORN.ALT + total_stems_per_ha_ULMU.RUB +

total_stems_per_ha_JUGL.CIN + total_stems_per_ha_PINU.RES + total_stems_per_ha_ALNU.VIR +

total_stems_per_ha_RHAM.SPP + total_stems_per_ha_PICE.ABI + total_stems_per_ha_PRUN.VIR +

total_stems_per_ha_ACER.NIG + total_stems_per_ha_POPU.DEL + total_stems_per_ha_MALU.SPP +

total_stems_per_ha_ULMU.THO + total_stems_per_ha_POPU.SPP + total_stems_per_ha_QUER.ALB +

total_stems_per_ha_CARP.CAR + total_stems_per_ha_QUER.BIC + total_stems_per_ha_HAMA.VIR +

total_stems_per_ha_JUNI.VIR + total_stems_per_ha_ACER.NEG + total_stems_per_ha_RHUS.TYP +

total_stems_per_ha_PINU.SYL + total_stems_per_ha_LARI.MAR + total_stems_per_ha_CARY.OVA +

total_stems_per_ha_PINU.RIG + total_stems_per_ha_CELT.OCC,

pct_density_ACER.SAH = total_stems_per_ha_ACER.SAH / total_density * 100,

pct_density_BETU.ALL = total_stems_per_ha_BETU.ALL / total_density * 100,

pct_density_FAGU.GRA = total_stems_per_ha_FAGU.GRA / total_density * 100,

total_basal_area = total_basal_area_m2_ACER.RUB + total_basal_area_m2_ACER.SAH +

total_basal_area_m2_FAGU.GRA + total_basal_area_m2_FRAX.AME + total_basal_area_m2_ULMU.AME +

total_basal_area_m2_OSTR.VIR + total_basal_area_m2_BETU.ALL + total_basal_area_m2_BETU.PAP +

total_basal_area_m2_ABIE.BAL + total_basal_area_m2_ACER.PEN + total_basal_area_m2_QUER.RUB +

total_basal_area_m2_TILI.AME + total_basal_area_m2_TSUG.CAN + total_basal_area_m2_PRUN.SER +

total_basal_area_m2_AMEL.SPP + total_basal_area_m2_UNKN.SPP + total_basal_area_m2_PICE.RUB +

total_basal_area_m2_PRUN.PEN + total_basal_area_m2_PICE.GLA + total_basal_area_m2_PICE.MAR +

total_basal_area_m2_THUJ.OCC + total_basal_area_m2_SORB.AME + total_basal_area_m2_ACER.SPI +

total_basal_area_m2_SORB.DEC + total_basal_area_m2_POPU.BAL + total_basal_area_m2_POPU.TRE +

total_basal_area_m2_SALI.SPP + total_basal_area_m2_PINU.STR + total_basal_area_m2_ALNU.RUG +

total_basal_area_m2_LARI.LAR + total_basal_area_m2_CRAT.SPP + total_basal_area_m2_FRAX.NIG +

total_basal_area_m2_QUER.MAC + total_basal_area_m2_POPU.GRA + total_basal_area_m2_PINU.BAN +

total_basal_area_m2_BETU.POP + total_basal_area_m2_ACER.SAC + total_basal_area_m2_FRAX.PEN +

total_basal_area_m2_CARY.COR + total_basal_area_m2_CORN.ALT + total_basal_area_m2_ULMU.RUB +

total_basal_area_m2_JUGL.CIN + total_basal_area_m2_PINU.RES + total_basal_area_m2_ALNU.VIR +

total_basal_area_m2_RHAM.SPP + total_basal_area_m2_PICE.ABI + total_basal_area_m2_PRUN.VIR +

total_basal_area_m2_ACER.NIG + total_basal_area_m2_POPU.DEL + total_basal_area_m2_MALU.SPP +

total_basal_area_m2_ULMU.THO + total_basal_area_m2_POPU.SPP + total_basal_area_m2_QUER.ALB +

total_basal_area_m2_CARP.CAR + total_basal_area_m2_QUER.BIC + total_basal_area_m2_HAMA.VIR +

total_basal_area_m2_JUNI.VIR + total_basal_area_m2_ACER.NEG + total_basal_area_m2_RHUS.TYP +

total_basal_area_m2_PINU.SYL + total_basal_area_m2_LARI.MAR + total_basal_area_m2_CARY.OVA +

total_basal_area_m2_PINU.RIG + total_basal_area_m2_CELT.OCC,

pct_basal_area_ACER.SAH = total_basal_area_m2_ACER.SAH / total_basal_area * 100,

pct_basal_area_BETU.ALL = total_basal_area_m2_BETU.ALL / total_basal_area * 100,

pct_basal_area_FAGU.GRA = total_basal_area_m2_FAGU.GRA / total_basal_area * 100)

# Export file

file_path <- "C:/Users/martigau/OneDrive - NRCan RNCan/Documents/Personnel/Travail/EcolEvol/NFI Data/QC_PSP/wide_df3.csv"

write.csv(wide_df3, file = file_path)

write.csv2(wide_df3, "wide_df3_fr.csv", row.names = FALSE)

#############################################################

#############################################################

##### Update the list of sites for Québec #######

#############################################################

#############################################################

# Step 1: Load the dataset

wide_df3 <- fread("C:/Users/martigau/OneDrive - NRCan RNCan/Documents/Personnel/Travail/EcolEvol/NFI Data/QC_PSP/wide_df3.csv")

colnames(wide_df3)

print(wide_df3)

unique(wide_df3$"pct_density_total_stems_per_ha_ACER.SAH")

# Check for duplicate column names

anyDuplicated(names(wide_df3))

# Ensure column names are unique

names(wide_df3) <- make.unique(names(wide_df3))

# Update the treatment_year where magp_site_id is '30001990'

wide_df3$treatment_year[wide_df3$magp_site_id == '30001990'] <- 1988

# Verify the changes

head(wide_df3[wide_df3$magp_site_id == '30001990', ])

# Import tree condition data - Replace with the path to your own CSV file

tree_condition <- fread("C:/Users/martigau/OneDrive - NRCan RNCan/Documents/Personnel/Travail/EcolEvol/NFI Data/QC_PSP/magp_tree_condition.csv")

print(tree_condition)

colnames(tree_condition)

# Merge with 'tree_condition' to have the number of trees with cankers, cracked bark due to BBD

bbd <- tree_condition %>%

filter(condition == "DSKT4") %>%

group_by(magp_site_id, plot_type_id, meas_num) %>%

summarise(count = n(), .groups = "drop") %>%

rename(bbd_count = count) # Renaming the count column to bbd_count

# Merge with original data frame to set bbd_count = 0 where missing

wide_df4 <- wide_df3 %>%

left_join(bbd, by = c("magp_site_id", "plot_type_id", "meas_num")) %>%

mutate(bbd_count = if_else(is.na(bbd_count), 0, bbd_count)) # Set missing bbd_count values to 0

# Print the result

print(wide_df4, n = 511)

#Calculate density/ha and % density of FAGU.GRA

wide_df5 <- wide_df4 %>%

mutate(pct_dens_bbd = ((bbd_count * 25) / total_stems_per_ha_FAGU.GRA) * 100)

head(wide_df5)

nrow(wide_df4)

nrow(wide_df5)

# Step 2: Filter for sugar maple stands (>50% basal area)

site_ids <- c(

'30000549', '30000558', '30000569', '30000581', '30000583', '30000601', '30000622', '30000673',

'30000713', '30000728', '30000733', '30000799', '30000841', '30000927', '30001001', '30001019',

'30001212', '30001226', '30001450', '30001451', '30001517', '30001574', '30001591', '30001623',

'30001644', '30001986', '30002011', '30002020', '30002047', '30002050', '30002021', '30002024', '30002027', '30002054',

'30002141', '30002160', '30002162', '30002178', '30002219', '30002220', '30002261', '30002359', '30002385', '30002431', '30002757', '30002794',

'30002822', '30002905', '30002923', '30002933', '30002936', '30002950', '30003054', '30003089',

'30003094', '30003174', '30003313', '30003529', '30003591', '30003610', '30004447', '30004479',

'30004492', '30005338', '30005529', '30005541', '30005546', '30005572', '30005629', '30005653',

'30005655', '30005662', '30005664', '30005698', '30005730', '30005750', '30005757', '30005758',

'30005821', '30005831', '30005838', '30005855', '30005876', '30005891', '30005903', '30005909',

'30005931', '30005932', '30005938', '30005943', '30005994', '30006013', '30006026', '30006037',

'30006061', '30006067', '30006068', '30006090', '30006348', '30006480', '30006594', '30006596', '30006622',

'30006627', '30006631', '30006642', '30006697', '30006700', '30006707', '30006748', '30006755',

'30006817', '30000592', '30000608', '30000609', '30000610', '30000667', '30000690', '30001502',

'30001508', '30001603', '30001983', '30001984', '30001985', '30001995', '30001999', '30002002',

'30002007', '30002040', '30002042', '30002043', '30002052', '30002063', '30002097', '30002127',

'30002156', '30002172', '30002186', '30002215', '30002271', '30002273', '30002287', '30002353',

'30002780', '30002781', '30002791', '30002832', '30002844', '30002845', '30002852', '30002853',

'30002855', '30002872', '30002879', '30002925', '30002938', '30002943', '30002962', '30002963',

'30003005', '30003295', '30003389', '30003466', '30005535', '30005697', '30005883', '30005884',

'30005885', '30006337', '30006576', '30006602', '30006603', '30006614', '30006623', '30006632',

'30006633', '30006641', '30006650', '30006670', '30006673', '30006677', '30006683', '30006690',

'30006691', '30006695', '30006720', '30006726', '30006728', '30006734', '30006736', '30006737',

'30006738', '30006746', '30006747', '30006859', '30000550', '30000554', '30000555', '30000740',

'30000763', '30000764', '30000765', '30000771', '30000789', '30000802', '30000913', '30000973',

'30000982', '30001435', '30001453', '30001543', '30001570', '30001584', '30001585', '30001594',

'30001642', '30002009', '30002140', '30002148', '30002415', '30002871', '30003097', '30003326',

'30003449', '30003492', '30004469', '30004478', '30004488', '30004489', '30004568', '30004613',

'30005542', '30005633', '30005678', '30005964', '30005999', '30006595', '30006704', '30001210',

'30001516', '30001595', '30001630', '30001635', '30001957', '30002129', '30002142', '30002294',

'30003042', '30003347', '30003413', '30005121', '30005579', '30005635', '30006405',

'30000548', '30000566', '30000574', '30000580', '30000582', '30000598', '30000599', '30000613',

'30000634', '30000635', '30000647', '30000649', '30000656', '30000658', '30000659', '30000681',

'30000684', '30000685', '30000712', '30000732', '30000734', '30000735', '30000736', '30000745',

'30000749', '30000770', '30000783', '30000793', '30000816', '30000817', '30000819', '30000824',

'30000843', '30000845', '30000878', '30000898', '30000900', '30000923', '30000937', '30001217',

'30001219', '30001395', '30001397', '30001417', '30001419', '30001432', '30001497', '30001580',

'30001588', '30001600', '30001616', '30001617', '30001620', '30001622', '30001638', '30001639',

'30001647', '30001650', '30001653', '30001654', '30002003', '30002010', '30002014', '30002034',

'30002100', '30002145', '30002218', '30002384', '30002413', '30002424', '30002425', '30002795',

'30002818', '30003336', '30003367', '30003552', '30003553', '30004438', '30004501', '30005515',

'30005518', '30005522', '30005523', '30005526', '30005545', '30005560', '30005562', '30005566',

'30005570', '30005596', '30005648', '30005676', '30005737', '30005738', '30005756', '30005764',

'30005825', '30005837', '30005847', '30005867', '30005873', '30005879', '30005880', '30005894',

'30005895', '30005898', '30005899', '30005900', '30005977', '30005983', '30006015', '30006024',

'30006029', '30006038', '30006379', '30006400', '30006403', '30006413', '30006414', '30006416',

'30006572', '30006585', '30006606', '30006611', '30006621', '30006628', '30006668', '30006669',

'30006679', '30006701', '30006714', '30006719', '30006722', '30006749', '30006812', '30007083',

'30007094', '30005127', '30006724', '30001975', '30001998', '30002157',

'30002202', '30002414', '30002823', '30002920', '30006710',

'30002051', '30002095', '30002165', '30006032', '30006539',

'30006680', '30007182', '30002260', '30002295', '30002358',

'30006472', '30006702', '30001952', '30002280', '30003583',

'30006711', '30002416', '30002001', '30006625', '30002922',

'30002114', '30003355', '30006121', '30001990'

)

# Filter the dataset based on site id

sm <- wide_df5 %>%

filter(magp_site_id %in% site_ids)

str(sm)

# Filter again by plot type to exclude L2

sm2 <- sm %>%

filter(plot_type_id %in% c('L1', 'S1'))

str(sm2)

# See full list of treatments

unique(sm2$treatment_type)

# Filter again by meas_num to exclude 6 - not enough data

sm4<- sm2%>%

filter(meas_num %in% c(0, 1, 2, 3, 4, 5))

str(sm4)

# Export file along with French version (_fr)

file_path <- "C:/Users/martigau/OneDrive - NRCan RNCan/Documents/Personnel/Travail/EcolEvol/NFI Data/QC_PSP/sm4.csv"

write.csv(sm4, file = file_path)

write.csv2(sm4, "sm4_fr.csv", row.names = FALSE)

### ADDITIONAL SCREENING REQUIRED TO FILTER OUT SITES THAT HAD DUPLICATES

### SEE R FILE 5_Combine_all_stats

FILE 2) COMPILE DATA ONTARIO

##########################################

##### COMPILE ONTARIO DATASET ############

##########################################

# IMPORT ONTARIO PSP DATA

library(data.table)

library(dplyr)

library(sf)

library(tidyr)

library(ggplot2)

library(knitr)

library(broom)

# Replace with the path to your CSV file

data_on <- fread("C:/Users/martigau/OneDrive - NRCan RNCan/Documents/Personnel/Travail/EcolEvol/NFI Data/ON_PSPPGP/magp_trees.csv")

# Data header

data_header_on <- fread("C:/Users/martigau/OneDrive - NRCan RNCan/Documents/Personnel/Travail/EcolEvol/NFI Data/ON_PSPPGP/magp_tree_header.csv")

# Ensure numeric columns are treated as numeric

data_on$dbh <- as.numeric(data_on$dbh)

data_header_on$meas_plot_size <- as.numeric(data_header_on$meas_plot_size)

data_header_on$meas_year <- as.numeric(data_header_on$meas_year)

# Separate saplings (S1) from commercial trees (L1)

data_on$plot_type_id2 <- with(data_on,

ifelse(plot_type_id == 'L1' & dbh < 9.1, 'S1',

ifelse(plot_type_id == 'L2' & dbh < 9.1, 'S2',

ifelse(plot_type_id == 'L3' & dbh < 9.1, 'S3', plot_type_id)))

)

# Display the updated data frame

print(data_on)

#Data on plot size

unique(data_header_on$meas_plot_size)

# List of sites

site_ids <- c(

"30452502", "30454614", "30454623", "30454624", "30454626", "30454634",

"30454637", "30454638", "30454657", "30454665", "30454692", "30454715",

"30454737", "30454764", "30454774", "30454778", "30454861", "30454897",

"30454914", "30454944", "30454969", "30454974", "30454976", "30454998",

"30455015", "30455097", "30452315", "30454633", "30454645", "30454647",

"30454654", "30454743", "30454747", "30455049", "30455076", "30454710",

"30454766", "30454773", "30454973", "30455003", "30455023", "30455115",

"30454942", "30455029", "30455108", "30452316", "30454617", "30454632",

"30454966", "30454977", "30452501", "30454621", "30454644", "30454664",

"30454779", "30454887", "30454955", "30454968", "30455017", "30455030",

"30454742", "30454776", "30454840", "30452512", "30453654", "30454474",

"30454656", "30454720", "30454761", "30454867", "30454639", "30454841",

"30454950", "30455002", "30454886", "30454954", "30454965", "30455016",

"30454852", "30452505", "30454643", "30454835", "30455482", "30452362",

"30453649", "30454671", "30454984", "30453576", "30454616", "30455034",

"30454563", "30454800", "30454983", "30454994", "30454615", "30454629",

"30454770", "30454881", "30454980", "30455098", "30453785", "30454631",

"30454839", "30453795", "30454658", "30455052", "30455090", "30454765",

"30454806", "30454815", "30455025", "30454900", "30454964", "30454979",

"30454988", "30455039", "30454620", "30454802", "30454903", "30455011",

"30454741", "30454769", "30454987", "30454660", "30454745", "30454995",

"30455013", "30454676", "30454810", "30454812", "30454874", "30454919",

"30455093", "30454619", "30454902", "30455114", "30453692", "30454618",

"30455006", "30455095", "30454803", "30452331", "30454739", "30454653",

"30454744", "30454878", "30454948", "30454963", "30455014", "30455032",

"30455072", "30455476", "30454799", "30454834", "30454736", "30454838",

"30454991", "30454628", "30454630", "30454642", "30454877", "30454898",

"30455012", "30455027", "30455073", "30452332", "30454801", "30454996",

"30450871", "30454836", "30455048", "30452319", "30453589", "30454625",

"30454708", "30454913", "30454999", "30453661", "30453723", "30455001",

"30455458", "30454837", "30454981", "30455019", "30455046", "30455060",

"30455118", "30454907", "30455045", "30455061", "30455101", "30454809",

"30454937", "30454655", "30454873", "30454908", "30455031", "30455094",

"30453792", "30454768", "30455102", "30452511", "30450865", "30453748",

"30455056", "30455502", "30450874", "30452326", "30454993", "30455036",

"30455077", "30450867", "30452333", "30454871", "30455112", "30452507",

"30454938", "30455000"

)

# Extract unique values of meas_plot_size for the specified magp_site_id values

unique_meas_plot_size <- data_header_on %>%

filter(magp_site_id %in% site_ids) %>% # Filter for the specified magp_site_ids

distinct(meas_plot_size) # Extract unique values of meas_plot_size

# Print the result

print(unique_meas_plot_size)

# Count the number of plots for each plot size

plot_count_by_size <- data_header_on %>%

filter(magp_site_id %in% site_ids) %>% # Filter for the specified magp_site_ids

group_by(meas_plot_size) %>% # Group by meas_plot_size

summarise(plot_count = n(), .groups = "drop") # Count the number of plots for each plot size

# Print the result

print(plot_count_by_size)

# A tibble: 2 × 2

# meas_plot_size plot_count

# <dbl> <int>

#1 0.04 918 (202 out of 218)

#2 0.1 45 (16 out of 218)

#>

# Count the number of re-measurements for each plot size (0.04 ha and 0.1 ha) for each magp_site_id

plot_count_by_size <- data_header_on %>%

filter(magp_site_id %in% site_ids, meas_plot_size %in% c(0.04, 0.1)) %>% # Filter for the specified site_ids and plot sizes

group_by(magp_site_id, meas_plot_size) %>% # Group by magp_site_id and meas_plot_size

summarise(plot_count = n(), .groups = "drop") # Count the number of re-measurements (rows)

# Print the result

print(plot_count_by_size, n = 218)

# Merge based on magp_site_id, plot_type_id, meas_num

merged_data_on <- merge(data_on, data_header_on, by = c("magp_site_id", "plot_type_id", "meas_num"))

# Specify the file path where you want to save the CSV file

file_path <- "C:/Users/martigau/OneDrive - NRCan RNCan/Documents/Personnel/Travail/EcolEvol/NFI Data/ON_PSPPGP/merged_data_on.csv"

write.csv(merged_data_on, file = file_path)

colnames(merged_data_on)

# Calculate basal area (m^2/ha)

merged_data_on$ba_ha <- (pi * (merged_data_on$dbh / 200)^2) / merged_data_on$meas_plot_size

write.csv(merged_data_on, file = file_path)

# VIEW DATA

merged_data_on

head(merged_data_on, 10)

str(merged_data_on)

names(merged_data_on)

# KEEP ONLY THE LIVING TREES AND SAPLINGS

relevant_rows_on <- merged_data_on[merged_data_on$tree_status %in% c('LF', 'LS', 'L', '-1'), ]

file_path <- "C:/Users/martigau/OneDrive - NRCan RNCan/Documents/Personnel/Travail/EcolEvol/NFI Data/ON_PSPPGP/relevant_rows_on.csv"

write.csv(relevant_rows_on, file = file_path)

# SUM BASAL AREA FOR EACH TREE BY SITE, PLOT, MEAS_NUM, and SPECIES

library(dplyr)

# Sum basal area by experimental unit

ba_sum_on <- relevant_rows_on %>%

group_by(magp_site_id, plot_type_id2, meas_num, species_gs, meas_year) %>%

summarize(total_basal_area_m2 = sum(ba_ha),

total_stems_per_ha = sum(stem_ha))

names(ba_sum_on)

# Keep specific columns in summary_data

summary_data_filtered_on <- ba_sum_on %>%

select(magp_site_id, plot_type_id2, meas_num, meas_year, species_gs, total_stems_per_ha, total_basal_area_m2)

# View the filtered summary data

print(summary_data_filtered_on)

# Export file along with French version (_fr)

file_path <- "C:/Users/martigau/OneDrive - NRCan RNCan/Documents/Personnel/Travail/EcolEvol/NFI Data/ON_PSPPGP/summary_data_filtered_on.csv"

write.csv(summary_data_filtered_on, file = file_path)

write.csv2(summary_data_filtered_on, "summary_data_on_fr.csv", row.names = FALSE)

# Merge with 'magp_sites' to have lat/long/elev/aspect/slope

data_sites_on <- fread("C:/Users/martigau/OneDrive - NRCan RNCan/Documents/Personnel/Travail/EcolEvol/NFI Data/ON_PSPPGP/magp_sites.csv")

merged_data_sites_on <- merge(summary_data_filtered_on, data_sites_on, by = c("magp_site_id"), all.x = TRUE)

# Merge with 'magp_treatment'

data_treat_on <- fread("C:/Users/martigau/OneDrive - NRCan RNCan/Documents/Personnel/Travail/EcolEvol/NFI Data/ON_PSPPGP/magp_treatment.csv")

data_treat_on2 <- data_treat_on %>%

select(magp_site_id, treatment_type, treatment_year, treatment_month, treatment_pct)

# Left join to keep 'control' plots

merged_site_treat_on <- merge(merged_data_sites_on, data_treat_on2, by = c("magp_site_id"), all.x = TRUE)

# Replace NA values in treatment_type with "Control"

merged_site_treat_on$treatment_type[is.na(merged_site_treat_on$treatment_type)] <- "CON"

# Check the resulting data

head(merged_site_treat_on)

# Specify the file path where you want to save the CSV file

file_path <- "C:/Users/martigau/OneDrive - NRCan RNCan/Documents/Personnel/Travail/EcolEvol/NFI Data/ON_PSPPGP/merged_site_treat_on.csv"

write.csv(merged_site_treat_on, file = file_path)

write.csv2(merged_site_treat_on, "merged_site_treat_on_fr.csv", row.names = FALSE)

#### PIVOT TABLE TIME - PUT SPECIES IN EACH COLUMN

#Pivot the data from long format to wide format, where species are columns

library(dplyr)

library(tidyr)

wide_df_on <- merged_site_treat_on %>%

pivot_wider(names_from = species_gs,

values_from = c(total_stems_per_ha, total_basal_area_m2))

# Replace NA with 0

wide_df_on2 <- wide_df_on %>%

mutate(across(starts_with("total_stems_per_ha_"), ~replace(., is.na(.), 0)),

across(starts_with("total_basal_area_m2_"), ~replace(., is.na(.), 0)))

# Print the result

print(wide_df_on2)

# Export file wide_df_on

file_path <- "C:/Users/martigau/OneDrive - NRCan RNCan/Documents/Personnel/Travail/EcolEvol/NFI Data/ON_PSPPGP/wide_df_on.csv"

write.csv(wide_df_on, file = file_path)

write.csv2(wide_df_on, "wide_df_on_fr.csv", row.names = FALSE)

# Export file wide_df_on2

file_path <- "C:/Users/martigau/OneDrive - NRCan RNCan/Documents/Personnel/Travail/EcolEvol/NFI Data/ON_PSPPGP/wide_df_on2.csv"

write.csv(wide_df_on2, file = file_path)

write.csv2(wide_df_on2, "pivot_table_species_on_fr.csv", row.names = FALSE)

# IDENTIFY UNIQUE SPECIES LIST FOR ONTARIO, n = 79

unique_species <- unique(relevant_rows_on$species_gs)

# Print the unique species

print(unique_species)

#Save as .csv file

file_path <- "C:/Users/martigau/OneDrive - NRCan RNCan/Documents/Personnel/Travail/EcolEvol/NFI Data/ON_PSPPGP/unique_species.csv"

write.csv(unique_species, file = file_path)

### ADD DENSITY AND BASAL AREA TOTAL FOR ONTARIO SPECIES

library(dplyr)

library(tidyr)

# List of species from your unique list

species_list <- c(

"BETU.PAP", "LARI.LAR", "POPU.BAL", "POPU.TRE", "PICE.MAR", "PINU.BAN",

"ABIE.BAL", "PICE.GLA", "PINU.RES", "ACER.RUB", "SORB.AME", "SALI.SPP",

"THUJ.OCC", "FRAX.NIG", "PINU.STR", "FRAX.AME", "OSTR.VIR", "QUER.MAC",

"TILI.AME", "POPU.GRA", "SORB.SPP", "PRUN.PEN", "ACER.SAH", "SALI.PEN",

"PICE.ABI", "AMEL.SPP", "SORB.DEC", "BETU.ALL", "UNKN.SPP", "QUER.SHU",

"QUER.RUB", "PRUN.SER", "LIRI.TUL", "PINU.SYL", "ULMU.AME", "ACER.PEN",

"TSUG.CAN", "FAGU.GRA", "LARI.DEC", "GENH.SPP", "JUNI.VIR", "PICE.RUB",

"QUER.ALB", "POPU.SPP", "SORB.AUC", "ULMU.RUB", "JUGL.NIG", "QUER.PAL",

"ACER.NIG", "CARY.COR", "FRAX.PEN", "CARY.OVA", "CARP.CAR", "ACER.SAC",

"CELT.OCC", "ROBI.PSE", "JUGL.CIN", "CRAT.SPP", "QUER.VEL", "SASS.ALB",

"QUER.BIC", "QUER.MUE", "CAST.DEN", "RHAM.CAT", "CARY.BIC", "PRUN.AVI",

"ULMU.THO", "MAGN.ACU", "SALI.ALB", "NYSS.SYL", "CAST.SAT", "POPU.CAN",

"ACER.NEG", "TILI.PLA", "PLAT.OCC", "PINU.RIG", "BETU.POP", "POPU.DEL",

"SALI.BAB"

)

# Generate column names for the species in the list

stem_columns <- paste0("total_stems_per_ha_", species_list)

basal_area_columns <- paste0("total_basal_area_m2_", species_list)

# Ensure the columns exist in your dataframe

stem_columns <- intersect(stem_columns, names(wide_df_on2))

basal_area_columns <- intersect(basal_area_columns, names(wide_df_on2))

# Perform the calculations

wide_df_on3 <- wide_df_on2 %>%

rowwise() %>%

mutate(

total_density = sum(c_across(all_of(stem_columns)), na.rm = TRUE),

total_basal_area = sum(c_across(all_of(basal_area_columns)), na.rm = TRUE)

) %>%

ungroup() %>%

mutate(

# Create percentage columns for density

across(

starts_with("total_stems_per_ha_"),

~ . / (total_density + 1e-6) * 100,

.names = "pct_density_{col}"

),

# Create percentage columns for basal area

across(

starts_with("total_basal_area_m2_"),

~ . / (total_basal_area + 1e-6) * 100,

.names = "pct_basal_area_{col}"

)

) %>%

# Add columns for total sums

mutate(

total_density_sum = total_density,

total_basal_area_sum = total_basal_area

)

# Extract percentage columns

percentage_columns <- names(wide_df_on3) %>%

.[grepl("pct_density_|pct_basal_area_", .)]

# Columns to keep from the original dataframe

additional_columns <- c(

"magp_site_id", "plot_type_id2", "meas_num", "meas_year", "V1",

"province", "latitude", "longitude", "elevation", "aspect_class",

"slope_class", "slope_position", "gpp_type", "sources", "treatment_type",

"treatment_year", "treatment_month", "treatment_pct"

)

# Ensure the additional columns are in the dataframe

additional_columns <- intersect(additional_columns, names(wide_df_on2))

# Create the final dataframe with percentage columns, additional columns, and total sum columns

wide_df_on3_final <- wide_df_on3 %>%

select(all_of(additional_columns), all_of(percentage_columns), total_density_sum, total_basal_area_sum)

# Save the modified data frame to CSV

write.csv(wide_df_on3_final, file = "C:/Users/martigau/OneDrive - NRCan RNCan/Documents/Personnel/Travail/EcolEvol/NFI Data/ON_PSPPGP/wide_df_on3.csv", row.names = FALSE)

write.csv2(wide_df_on3_final, file = "C:/Users/martigau/OneDrive - NRCan RNCan/Documents/Personnel/Travail/EcolEvol/NFI Data/ON_PSPPGP/wide_df_on3_fr.csv", row.names = FALSE)

# Your dataset

wide_df_on3 <- fread("C:/Users/martigau/OneDrive - NRCan RNCan/Documents/Personnel/Travail/EcolEvol/NFI Data/ON_PSPPGP/wide_df_on3.csv")

print(wide_df_on3)

# Step 1: Check for duplicate column names, NONE found

anyDuplicated(names(wide_df_on3))

# Rename the main dependent variables:

wide_df_on4 <- wide_df_on3 %>%

rename(pct_basal_area_ACER.SAH = pct_basal_area_total_basal_area_m2_ACER.SAH,

pct_basal_area_FAGU.GRA = pct_basal_area_total_basal_area_m2_FAGU.GRA,

pct_basal_area_BETU.ALL = pct_basal_area_total_basal_area_m2_BETU.ALL

)

#View data

print(wide_df_on4)

# Import tree condition data - Replace with the path to your own CSV file

tree_condition_on <- fread("C:/Users/martigau/OneDrive - NRCan RNCan/Documents/Personnel/Travail/EcolEvol/NFI Data/ON_PSPPGP/magp_tree_condition.csv")

# Merge with 'tree_condition' to have the number of trees with cankers, cracked bark due to BBD

bbd_on <- tree_condition_on %>%

filter(condition == "DSKT") %>%

group_by(magp_site_id, plot_type_id, meas_num) %>%

summarise(count = n(), .groups = "drop") %>%

rename(bbd_count = count) # Renaming the count column to bbd_count

print(bbd_on, n = 141)

#Keep only L1

bbd_on2 <- bbd_on %>%

filter(plot_type_id == "L1")

print(bbd_on2, n = 53)

# Rename the column plot_type_id to plot_type_id2

bbd_on3 <- bbd_on2 %>%

rename(plot_type_id2 = plot_type_id)

# Merge with original data frame (assuming it's 'wide_df4') to set bbd_count = 0 where missing

wide_df_on5 <- wide_df_on4 %>%

left_join(bbd_on3, by = c("magp_site_id", "plot_type_id2", "meas_num")) %>%

mutate(bbd_count = if_else(is.na(bbd_count), 0, bbd_count)) # Set missing bbd_count values to 0

# Print the result

print(wide_df_on5)

colnames(wide_df_on5)

# Save the modified data frame to CSV

write.csv(wide_df_on5, file = "C:/Users/martigau/OneDrive - NRCan RNCan/Documents/Personnel/Travail/EcolEvol/NFI Data/ON_PSPPGP/wide_df_on5.csv", row.names = FALSE)

write.csv2(wide_df_on5, file = "C:/Users/martigau/OneDrive - NRCan RNCan/Documents/Personnel/Travail/EcolEvol/NFI Data/ON_PSPPGP/wide_df_on5_fr.csv", row.names = FALSE)

colnames(wide_df_on5)

head(wide_df_on5)

# Add per hectare values of bbd count

wide_df_on6 <- wide_df_on5 %>%

mutate(pct_dens_bbd = case_when(

magp_site_id %in% c("30450865", "30450867", "30450871",

"30450874", "30452315", "30452316",

"30452331", "30452332", "30452333",

"30452362", "30452501", "30452502",

"30452505", "30452507", "30452511",

"30452512" ) ~ ((bbd_count * 10 / 0.1) / (pct_density_total_stems_per_ha_FAGU.GRA * total_density_sum)) * 100,

TRUE ~ ((bbd_count * 25 / 0.04) / (pct_density_total_stems_per_ha_FAGU.GRA * total_density_sum)) * 100

))

nrow(wide_df_on5)

nrow(wide_df_on6)

colnames(wide_df_on6)

# Save the modified data frame to CSV

write.csv(wide_df_on6, file = "C:/Users/martigau/OneDrive - NRCan RNCan/Documents/Personnel/Travail/EcolEvol/NFI Data/ON_PSPPGP/wide_df_on6.csv", row.names = FALSE)

write.csv2(wide_df_on6, file = "C:/Users/martigau/OneDrive - NRCan RNCan/Documents/Personnel/Travail/EcolEvol/NFI Data/ON_PSPPGP/wide_df_on6_fr.csv", row.names = FALSE)

# Filter, select relevant variables, keep one row per magp_site_id, and sort by magp_site_id

sugar_maple_on <- wide_df_on6 %>%

filter(plot_type_id2 == "L1" & meas_num == 0 & pct_basal_area_ACER.SAH >= 50) %>%

select(magp_site_id, plot_type_id2, meas_num, meas_year, pct_basal_area_ACER.SAH) %>%

distinct(magp_site_id, .keep_all = TRUE) %>%

arrange(magp_site_id)

print(sugar_maple_on, n = 219)

str(sugar_maple_on)

# Save the modified data frame to CSV

write.csv(sugar_maple_on, file = "C:/Users/martigau/OneDrive - NRCan RNCan/Documents/Personnel/Travail/EcolEvol/NFI Data/ON_PSPPGP/sugar_maple_on.csv", row.names = FALSE)

write.csv2(sugar_maple_on, file = "C:/Users/martigau/OneDrive - NRCan RNCan/Documents/Personnel/Travail/EcolEvol/NFI Data/ON_PSPPGP/sugar_maple_on_fr.csv", row.names = FALSE)

# Data for the columns

magp_site_id_original <- c(

30452315, 30452316, 30452319, 30452326, 30452331, 30452332, 30452333, 30452362,

30452501, 30452502, 30452504, 30452505, 30452507, 30452511, 30452512, 30453576,

30453589, 30453649, 30453654, 30453661, 30453692, 30453723, 30453748, 30453785,

30453792, 30453795, 30454474, 30454563, 30454614, 30454615, 30454616, 30454617,

30454618, 30454619, 30454620, 30454621, 30454623, 30454624, 30454625, 30454626,

30454628, 30454629, 30454630, 30454631, 30454632, 30454633, 30454634, 30454637,

30454638, 30454639, 30454642, 30454643, 30454644, 30454645, 30454646, 30454647,

30454653, 30454654, 30454655, 30454656, 30454657, 30454658, 30454660, 30454664,

30454665, 30454671, 30454675, 30454676, 30454692, 30454708, 30454710, 30454715,

30454720, 30454736, 30454737, 30454739, 30454741, 30454742, 30454743, 30454744,

30454745, 30454747, 30454761, 30454764, 30454765, 30454766, 30454768, 30454769,

30454770, 30454773, 30454774, 30454776, 30454778, 30454779, 30454799, 30454800,

30454801, 30454802, 30454803, 30454806, 30454809, 30454810, 30454812, 30454815,

30454834, 30454835, 30454836, 30454837, 30454838, 30454839, 30454840, 30454841,

30454852, 30454861, 30454867, 30454871, 30454873, 30454874, 30454877, 30454878,

30454881, 30454886, 30454887, 30454897, 30454898, 30454900, 30454902, 30454903,

30454907, 30454908, 30454913, 30454914, 30454919, 30454937, 30454938, 30454942,

30454944, 30454948, 30454950, 30454954, 30454955, 30454963, 30454964, 30454965,

30454966, 30454968, 30454969, 30454973, 30454974, 30454976, 30454977, 30454979,

30454980, 30454981, 30454983, 30454984, 30454987, 30454988, 30454991, 30454993,

30454994, 30454995, 30454996, 30454998, 30454999, 30455001, 30455002, 30455003,

30455006, 30455011, 30455012, 30455013, 30455014, 30455015, 30455016, 30455017,

30455019, 30455023, 30455025, 30455027, 30455029, 30455030, 30455031, 30455032,

30455034, 30455036, 30455039, 30455045, 30455046, 30455048, 30455049, 30455052,

30455056, 30455060, 30455061, 30455072, 30455073, 30455076, 30455077, 30455090,

30455093, 30455094, 30455095, 30455097, 30455098, 30455101, 30455108, 30455112,

30455114, 30455115, 30455118, 30455123, 30455458, 30455476, 30455482, 30455502

)

magp_site_id_new <- c(

30450865, 30450867, 30450871, 30450874, 30452315, 30452316, 30452319, 30452326,

30452331, 30452332, 30452333, 30452362, 30452501, 30452502, 30452505, 30452507,

30452511, 30452512, 30453576, 30453589, 30453649, 30453654, 30453661, 30453692,

30453723, 30453748, 30453785, 30453792, 30453795, 30454474, 30454563, 30454614,

30454615, 30454616, 30454617, 30454618, 30454619, 30454620, 30454621, 30454623,

30454624, 30454625, 30454626, 30454628, 30454629, 30454630, 30454631, 30454632,

30454633, 30454634, 30454637, 30454638, 30454639, 30454642, 30454643, 30454644,

30454645, 30454646, 30454647, 30454653, 30454654, 30454655, 30454656, 30454657,

30454658, 30454660, 30454664, 30454665, 30454671, 30454675, 30454676, 30454692,

30454708, 30454710, 30454715, 30454720, 30454736, 30454737, 30454739, 30454741,

30454742, 30454743, 30454744, 30454745, 30454747, 30454761, 30454764, 30454765,

30454766, 30454768, 30454769, 30454770, 30454773, 30454774, 30454776, 30454778,

30454779, 30454799, 30454776, 30454779, 30454799, 30454800, 30454801, 30454802,

30454803, 30454806, 30454809, 30454810, 30454812, 30454815, 30454834, 30454835,

30454836, 30454837, 30454838, 30454839, 30454840, 30454841, 30454852, 30454861,

30454867, 30454871, 30454873, 30454874, 30454877, 30454878, 30454881, 30454886,

30454887, 30454897, 30454898, 30454900, 30454902, 30454903, 30454907, 30454908,

30454913, 30454914, 30454919, 30454937, 30454938, 30454942, 30454944, 30454948,

30454950, 30454954, 30454955, 30454963, 30454964, 30454965, 30454966, 30454968,

30454969, 30454973, 30454974, 30454976, 30454977, 30454979, 30454980, 30454981,

30454983, 30454984, 30454987, 30454988, 30454991, 30454993, 30454994, 30454995,

30454996, 30454998, 30454999, 30455001, 30455002, 30455003, 30455006, 30455011,

30455012, 30455013, 30455014, 30455015, 30455016, 30455017, 30455019, 30455023,

30455025, 30455027, 30455029, 30455030, 30455031, 30455032, 30455034, 30455036,

30455039, 30455045, 30455046, 30455048, 30455049, 30455052, 30455056, 30455060,

30455061, 30455072, 30455073, 30455076, 30455077, 30455090, 30455093, 30455094,

30455095, 30455097, 30455098, 30455101, 30455108, 30455112, 30455114, 30455115,

30455118, 30455123, 30455458, 30455476, 30455482, 30455502

)

# Convert to sets to find differences

original_set <- unique(magp_site_id_original)

new_set <- unique(magp_site_id_new)

# Find unique values in each

unique_in_original <- setdiff(original_set, new_set)

unique_in_new <- setdiff(new_set, original_set)

# Print the results

cat("Unique values in 'magp_site_id_original' but not in 'magp_site_id_new':\n")

print(unique_in_original)

cat("\nUnique values in 'magp_site_id_new' but not in 'magp_site_id_original':\n")

print(unique_in_new)

# print(unique_in_original)

#[1] 30452504: value very close to 50

# print(unique_in_new)

#[1] 30450865 30450867 30450871 30450874: various treatments in plots

# Step 2: Filter sugar maple stands (>=50% BA)

site_ids <- c(

"30452502", "30454614", "30454623", "30454624", "30454626", "30454634",

"30454637", "30454638", "30454657", "30454665", "30454692", "30454715",

"30454737", "30454764", "30454774", "30454778", "30454861", "30454897",

"30454914", "30454944", "30454969", "30454974", "30454976", "30454998",

"30455015", "30455097", "30452315", "30454633", "30454645", "30454647",

"30454654", "30454743", "30454747", "30455049", "30455076", "30454710",

"30454766", "30454773", "30454973", "30455003", "30455023", "30455115",

"30454942", "30455029", "30455108", "30452316", "30454617", "30454632",

"30454966", "30454977", "30452501", "30454621", "30454644", "30454664",

"30454779", "30454887", "30454955", "30454968", "30455017", "30455030",

"30454742", "30454776", "30454840", "30452512", "30453654", "30454474",

"30454656", "30454720", "30454761", "30454867", "30454639", "30454841",

"30454950", "30455002", "30454886", "30454954", "30454965", "30455016",

"30454852", "30452505", "30454643", "30454835", "30455482", "30452362",

"30453649", "30454671", "30454984", "30453576", "30454616", "30455034",

"30454563", "30454800", "30454983", "30454994", "30454615", "30454629",

"30454770", "30454881", "30454980", "30455098", "30453785", "30454631",

"30454839", "30453795", "30454658", "30455052", "30455090", "30454765",

"30454806", "30454815", "30455025", "30454900", "30454964", "30454979",

"30454988", "30455039", "30454620", "30454802", "30454903", "30455011",

"30454741", "30454769", "30454987", "30454660", "30454745", "30454995",

"30455013", "30454676", "30454810", "30454812", "30454874", "30454919",

"30455093", "30454619", "30454902", "30455114", "30453692", "30454618",

"30455006", "30455095", "30454803", "30452331", "30454739", "30454653",

"30454744", "30454878", "30454948", "30454963", "30455014", "30455032",

"30455072", "30455476", "30454799", "30454834", "30454736", "30454838",

"30454991", "30454628", "30454630", "30454642", "30454877", "30454898",

"30455012", "30455027", "30455073", "30452332", "30454801", "30454996",

"30450871", "30454836", "30455048", "30452319", "30453589", "30454625",

"30454708", "30454913", "30454999", "30453661", "30453723", "30455001",

"30455458", "30454837", "30454981", "30455019", "30455046", "30455060",

"30455118", "30454907", "30455045", "30455061", "30455101", "30454809",

"30454937", "30454655", "30454873", "30454908", "30455031", "30455094",

"30453792", "30454768", "30455102", "30452511", "30450865", "30453748",

"30455056", "30455502", "30450874", "30452326", "30454993", "30455036",

"30455077", "30450867", "30452333", "30454871", "30455112", "30452507",

"30454938", "30455000"

)

# Filter the dataset based on site id

sm_on <- wide_df_on6 %>%

filter(magp_site_id %in% site_ids)

print(sm_on)

# Filter again by plot type to exclude L2

sm_on2 <- sm_on %>%

filter(plot_type_id2 %in% c('L1', 'S1'))

print(sm_on2)

# Filter again by treatment to keep CON - there are no harvested plots

sm_on3 <- sm_on2 %>%

filter(treatment_type %in% c('CON'))

# Filter again by meas_num to exclude 5 and 6

sm_on4<- sm_on3%>%

filter(meas_num %in% c(0, 1, 2, 3, 4))

nrow(sm_on3)

nrow(sm_on4)

# Export file along with French version (_fr)

file_path <- "C:/Users/martigau/OneDrive - NRCan RNCan/Documents/Personnel/Travail/EcolEvol/NFI Data/ON_PSPPGP/sm_on4.csv"

write.csv(sm_on4, file = file_path)

write.csv2(sm_on4, "sm4_on_fr.csv", row.names = FALSE)

### NEEDS FURTHER COMPILATION

### SEE R FILE 5_Combine_all_stats

FILE 3) COMPILE NEW BRUNSWICK DATA

##############################

#### NEW BRUNSWICK DATA ######

##############################

# Upload packages

library(data.table)

library(dplyr)

library(sf)

library(tidyr)

library(ggplot2)

library(knitr)

library(broom)

# Replace with the path to your own CSV file

data_nb <- fread("C:/Users/martigau/OneDrive - NRCan RNCan/Documents/Personnel/Travail/EcolEvol/NFI Data/NB_CLI_NoHolderId_16/magp_trees_nohld_16.csv")

data_header_nb <- fread("C:/Users/martigau/OneDrive - NRCan RNCan/Documents/Personnel/Travail/EcolEvol/NFI Data/NB_CLI_NoHolderId_16/magp_tree_header_nohld_16.csv")

colnames(data_nb)

###TROUBLESHOOTING

# Check for duplicate key combinations in 'data_header_nb'

duplicates_header_nb <- data_header_nb %>%

group_by(magp_site_id, plot_type_id, meas_num) %>%

filter(n() > 1) %>%

summarize(count = n())

print(duplicates_header_nb, n = 900)

# Remove duplicates from 'data_header_nb'

data_header_nb2 <- data_header_nb %>%

distinct(magp_site_id, plot_type_id, meas_num, .keep_all = TRUE)

# Perform the merge

merged_data_nb <- merge(data_nb, data_header_nb2, by = c("magp_site_id", "plot_type_id", "meas_num"))

colnames(merged_data_nb)

str(merged_data_nb)

# Export file along with French version (_fr)

file_path <- "C:/Users/martigau/OneDrive - NRCan RNCan/Documents/Personnel/Travail/EcolEvol/NFI Data/NB_CLI_NoHolderId_16/merged_data_nb.csv"

write.csv(merged_data_nb, file = file_path)

write.csv2(merged_data_nb, "merged_data_nb_fr.csv", row.names = FALSE)

# Exclude rows where dbh = -1

merged_data_nb2 <- merged_data_nb[merged_data_nb$dbh != -1, ]

data_30308718 <- merged_data_nb %>%

filter(magp_site_id == 30308718)

print(data_30308718)

# Info on plot size: 0.04 for L1 and 0.005 ha for S1

colnames(merged_data_nb2)

unique(merged_data_nb2$meas_plot_size)

# Calculate basal area (m^2/ha)

merged_data_nb2$ba_ha <- (pi * (merged_data_nb2$dbh / 200)^2) / merged_data_nb2$meas_plot_size

# VIEW DATA

merged_data_nb2

head(merged_data_nb2, 10)

str(merged_data_nb2)

names(merged_data_nb2)

# KEEP ONLY THE LIVING TREES AND SAPLINGS

relevant_rows_nb <- merged_data_nb2[

merged_data_nb2$tree_status %in% c('LF', 'LS', 'L', '-1')

#(merged_data_nb2$dbh < 9.1 | merged_data_nb2$plot_type_id == 'S1'),

]

relevant_rows_nb2 <- relevant_rows_nb %>%

mutate(relevant_rows_nb$dbh < 9.1 | relevant_rows_nb$plot_type_id == 'S1')

print(relevant_rows_nb2)

str(relevant_rows_nb2)

unique(relevant_rows_nb2$plot_type_id)

# CALCULATE BASAL AREA FOR EACH TREE BY SITE, PLOT, MEAS_NUM, and SPECIES

library(dplyr)

# Sum basal area by experimental unit

ba_sum_nb <- relevant_rows_nb2 %>%

group_by(magp_site_id, plot_type_id, meas_num, species_gs, meas_year) %>%

summarize(total_basal_area_m2 = sum(ba_ha),

total_stems_per_ha = sum(stem_ha))

print(ba_sum_nb)

# Keep specific columns in summary_data

summary_data_filtered_nb <- ba_sum_nb %>%

select(magp_site_id, plot_type_id, meas_num, species_gs, total_stems_per_ha, total_basal_area_m2, meas_year)

# View the filtered summary data

print(summary_data_filtered_nb)

# Export file along with French version (_fr)

file_path <- "C:/Users/martigau/OneDrive - NRCan RNCan/Documents/Personnel/Travail/EcolEvol/NFI Data/NB_CLI_NoHolderId_16/summary_data_filtered_nb.csv"

write.csv(summary_data_filtered_nb, file = file_path)

write.csv2(summary_data_filtered_nb, "summary_data_fr_nb.csv", row.names = FALSE)

# Merge with 'magp_sites' to have lat/long/elev/aspect/slope

data_sites_nb <- fread("C:/Users/martigau/OneDrive - NRCan RNCan/Documents/Personnel/Travail/EcolEvol/NFI Data/NB_CLI_NoHolderId_16/magp_sites_nohld_16.csv")

merged_data_sites_nb <- merge(summary_data_filtered_nb, data_sites_nb, by = c("magp_site_id"), all.x = TRUE)

# Merge with 'magp_treatment'

data_treat_nb <- fread("C:/Users/martigau/OneDrive - NRCan RNCan/Documents/Personnel/Travail/EcolEvol/NFI Data/NB_CLI_NoHolderId_16/magp_treatment_nohld_16.csv")

data_treat_nb2 <- data_treat_nb %>%

select(magp_site_id, treatment_type, treatment_year, treatment_month, treatment_pct)

# Left join to keep control plots

merged_site_treat_nb <- merge(merged_data_sites_nb, data_treat_nb2, by = c("magp_site_id"), all.x = TRUE)

# Replace NA values in treatment_type with "Control"

merged_site_treat_nb$treatment_type[is.na(merged_site_treat_nb$treatment_type)] <- "CON"

# Check the resulting data

head(merged_site_treat_nb)

# Specify the file path where you want to save the CSV file

file_path <- "C:/Users/martigau/OneDrive - NRCan RNCan/Documents/Personnel/Travail/EcolEvol/NFI Data/NB_CLI_NoHolderId_16/merged_site_treat_nb.csv"

write.csv(merged_site_treat_nb, file = file_path)

write.csv2(merged_site_treat_nb, "merged_site_treat_fr.csv", row.names = FALSE)

#Identify duplicates

# Load necessary libraries

library(dplyr)

# Identify duplicates

duplicates <- merged_site_treat_nb %>%

group_by(magp_site_id, plot_type_id, meas_num, species_gs) %>%

filter(n() > 1) %>%

summarise(duplicate_count = n(), .groups = 'drop')

print(n = 3224, duplicates)

#Remove duplicates, those are errors

# Remove duplicates, keeping only the first occurrence

merged_site_treat_nb2 <- merged_site_treat_nb %>%

distinct(magp_site_id, plot_type_id, meas_num, species_gs, .keep_all = TRUE)

#### PIVOT TABLE TIME - PUT SPECIES IN EACH COLUMN

#Pivot the data from long format to wide format, where species are columns

library(dplyr)

library(tidyr)

wide_df_nb <- merged_site_treat_nb2 %>%

pivot_wider(names_from = species_gs,

values_from = c(total_stems_per_ha, total_basal_area_m2))

# Replace NA with 0

wide_df_nb2 <- wide_df_nb %>%

mutate(across(starts_with("total_stems_per_ha_"), ~replace(., is.na(.), 0)),

across(starts_with("total_basal_area_m2_"), ~replace(., is.na(.), 0)))

# Print the result

print(wide_df_nb2)

# Export file wide_df_nb2

file_path <- "C:/Users/martigau/OneDrive - NRCan RNCan/Documents/Personnel/Travail/EcolEvol/NFI Data/NB_CLI_NoHolderId_16/wide_df_nb2.csv"

write.csv(wide_df_nb2, file = file_path)

write.csv2(wide_df_nb2, "pivot_table_species_nb_fr.csv", row.names = FALSE)

# IDENTIFY UNIQUE SPECIES LIST FOR NEW BRUNSWICK, n = 52

unique_species_nb <- unique(relevant_rows_nb2$species_gs)

# Print the unique species

print(unique_species_nb)

#Save as .csv file

file_path <- "C:/Users/martigau/OneDrive - NRCan RNCan/Documents/Personnel/Travail/EcolEvol/NFI Data/NB_CLI_NoHolderId_16/unique_species_nb.csv"

write.csv(unique_species_nb, file = file_path)

### ADD DENSITY AND BASAL AREA TOTAL FOR NEW BRUNSWICK SPECIES

library(dplyr)

library(tidyr)

# List of species from your unique list

species_list_nb <- c(

"LARI.LAR", "ABIE.BAL", "BETU.PAP", "PICE.RUB",

"BETU.ALL", "PICE.GLA", "ALNU.SPP", "PICE.MAR", "PINU.BAN",

"ACER.RUB", "UNKN.SPP", "SORB.AME", "POPU.TRE", "ACER.PEN",

"ACER.SPI", "PRUN.PEN", "SALI.SPP", "AMEL.SPP", "THUJ.OCC",

"POPU.GRA", "BETU.POP", "PINU.STR", "PRUN.VIR", "OSTR.VIR",

"FAGU.GRA", "TSUG.CAN", "CORN.ALT", "VIBU.CAS", "PRUN.SER",

"JUGL.CIN", "ILEX.MUC", "CRAT.SPP", "MALU.SPP", "FRAX.AME",

"ACER.SAH", "FRAX.NIG", "QUER.SPP", "PINU.RES", "GENH.SPP",

"POPU.BAL", "ULMU.SPP", "PICE.ABI", "GENC.SPP", "FRAX.PEN",

"ACER.SAC", "SAMB.NIG", "SAMB.RAC", "VIBU.LAN", "TILI.AME",

"PINU.SYL", "GLED.TRI", "RUBU.OCC"

)

# Generate column names for the species in the list

stem_columns <- paste0("total_stems_per_ha_", species_list_nb)

basal_area_columns <- paste0("total_basal_area_m2_", species_list_nb)

# Ensure the columns exist in your dataframe

stem_columns <- intersect(stem_columns, names(wide_df_nb2))

basal_area_columns <- intersect(basal_area_columns, names(wide_df_nb2))

# Perform the calculations

wide_df_nb3 <- wide_df_nb2 %>%

rowwise() %>%

mutate(

total_density = sum(c_across(all_of(stem_columns)), na.rm = TRUE),

total_basal_area = sum(c_across(all_of(basal_area_columns)), na.rm = TRUE)

) %>%

ungroup() %>%

mutate(

# Create percentage columns for density

across(

starts_with("total_stems_per_ha_"),

~ . / (total_density + 1e-6) * 100,

.names = "pct_density_{col}"

),

# Create percentage columns for basal area

across(

starts_with("total_basal_area_m2_"),

~ . / (total_basal_area + 1e-6) * 100,

.names = "pct_basal_area_{col}"

)

) %>%

# Add columns for total sums

mutate(

total_density_sum = total_density,

total_basal_area_sum = total_basal_area

)

# Extract percentage columns

percentage_columns <- names(wide_df_nb3) %>%

.[grepl("pct_density_|pct_basal_area_", .)]

# Columns to keep from the original dataframe

additional_columns <- c(

"magp_site_id", "plot_type_id", "meas_num", "meas_year", "V1",

"province", "latitude", "longitude", "elevation", "aspect_class",

"slope_class", "slope_position", "gpp_type", "sources", "treatment_type",

"treatment_year", "treatment_month", "treatment_pct"

)

# Ensure the additional columns are in the dataframe

additional_columns <- intersect(additional_columns, names(wide_df_nb2))

# Create the final dataframe with percentage columns, additional columns, and total sum columns

wide_df_nb3 <- wide_df_nb3 %>%

select(all_of(additional_columns), all_of(percentage_columns), total_density_sum, total_basal_area_sum)

# Save the modified data frame to CSV

write.csv(wide_df_nb3, file = "C:/Users/martigau/OneDrive - NRCan RNCan/Documents/Personnel/Travail/EcolEvol/NFI Data/NB_CLI_NoHolderId_16/wide_df_nb3.csv", row.names = FALSE)

write.csv2(wide_df_nb3, file = "C:/Users/martigau/OneDrive - NRCan RNCan/Documents/Personnel/Travail/EcolEvol/NFI Data/NB_CLI_NoHolderId_16/wide_df_nb3_fr.csv", row.names = FALSE)

# Export file wide_df_nb3

file_path <- "C:/Users/martigau/OneDrive - NRCan RNCan/Documents/Personnel/Travail/EcolEvol/NFI Data/NB_CLI_NoHolderId_16/wide_df_nb3.csv"

write.csv(wide_df_nb3, file = file_path)

write.csv2(wide_df_nb3, "pivot_table_species_nb_fr.csv", row.names = FALSE)

# Load dataset

wide_df_nb3 <- fread("C:/Users/martigau/OneDrive - NRCan RNCan/Documents/Personnel/Travail/EcolEvol/NFI Data/NB_CLI_NoHolderId_16/wide_df_nb3.csv")

# Step 1: Check for duplicate column names

anyDuplicated(names(wide_df_nb3))

# Ensure column names are unique

names(wide_df_nb3) <- make.unique(names(wide_df_nb3))

# Step 2: Filter (n = 300)

# Sugar maple (>=50% BA), TRT= CLR, CON

site_ids <- c(

"30307072", "30308005", "30307891", "30307159", "30308723", "30308456",

"30305960", "30306897", "30307307", "30304219", "30304352", "30301803",

"30308182", "30303283", "30301413", "30306155", "30306614", "30308788",

"30307258", "30301354", "30306762", "30308976", "30307996", "30307849",

"30306218", "30306608", "30308808", "30307984", "30306517", "30306679",

"30306681", "30303865", "30307066", "30306353", "30306884", "30306219",

"30308501", "30308042", "30307314", "30305971", "30305667", "30307439",

"30306099", "30307915", "30305974", "30308055", "30308516", "30301295",

"30304749", "30306358", "30307991", "30307569", "30307379", "30307832",

"30305220", "30303518", "30307237", "30308314", "30301752", "30306518",

"30308374", "30309026", "30301683", "30309024", "30308251", "30306434",

"30304894", "30304267", "30308268", "30306157", "30301039", "30306979",

"30307912", "30303866", "30304688", "30300494", "30307069", "30306903",

"30304269", "30300412", "30308648", "30308654", "30308586", "30302263",

"30307632", "30305176", "30308549", "30307970", "30308853", "30308441",

"30305110", "30301403", "30300986", "30307576", "30308065", "30306156",

"30306038", "30307464", "30304624", "30303449", "30306284", "30305883",

"30307985", "30300536", "30301471", "30307380", "30306682", "30303870",

"30308422", "30307722", "30305277", "30305169", "30304559", "30306750",

"30301038", "30306232", "30305184", "30304492", "30301635", "30301644",

"30307689", "30308635", "30306830", "30307129", "30306418", "30304838",

"30307064", "30307241", "30303766", "30306435", "30300653", "30306898",

"30306261", "30306801", "30304563", "30306662", "30306338", "30303375",

"30301148", "30305287", "30306287", "30305168", "30305771", "30306761",

"30304750", "30308066", "30308598", "30301192", "30303649", "30309025",

"30307562", "30303461", "30308053", "30307522", "30301407", "30307792",

"30301470", "30301414", "30301748", "30307828", "30305319", "30308212",

"30308718", "30305917", "30303131", "30304950", "30302899", "30308416",

"30308870", "30305107", "30305332", "30305069", "30301034", "30308799",

"30300527", "30303459", "30303321", "30301643", "30307895", "30306470",

"30309097", "30307831", "30302204", "30307882", "30304463", "30307062",

"30307947", "30305935", "30303528", "30308975", "30308810", "30306029",

"30304746", "30302870", "30307164", "30306977", "30307971", "30303863",

"30307256", "30303744", "30304214", "30308400", "30304044", "30301151",

"30306968", "30302683", "30300903", "30303513", "30303296", "30308795",

"30303133", "30304557", "30308096", "30307778", "30300797", "30308235",

"30304581", "30307855", "30307497", "30305772", "30304813", "30305062",

"30305234", "30307041", "30305209", "30300788", "30308518", "30307688",

"30308376", "30300839", "30301974", "30306502", "30303829", "30307149",

"30302834", "30303926", "30307221", "30301145", "30306620", "30308358",

"30307121", "30306890", "30307892", "30308968", "30308455", "30308361",

"30303026", "30301350", "30300229", "30301656", "30308468", "30303325",

"30301523", "30301111", "30301310", "30308855", "30304723", "30301973",

"30306873"

)

# Import tree condition data - Replace with the path to your own CSV file

tree_condition_nb <- fread("C:/Users/martigau/OneDrive - NRCan RNCan/Documents/Personnel/Travail/EcolEvol/NFI Data/NB_CLI_NoHolderId_16/magp_tree_condition_nohld_16.csv")

print(tree_condition_nb)

colnames(tree_condition_nb)

# Calculate the number of diseased trees (DI)

bbd_nb <- tree_condition_nb %>%

filter(condition == "DI") %>%

group_by(magp_site_id, plot_type_id, meas_num) %>%

summarise(count = n(), .groups = "drop") %>%

rename(bbd_count = count) # Renaming the count column to bbd_count

print(bbd_nb, n = 100)

# Merge with original data frame to set bbd_count = 0 where missing

wide_df_nb4 <- wide_df_nb3 %>%

left_join(bbd_nb, by = c("magp_site_id", "plot_type_id", "meas_num")) %>%

mutate(bbd_count = if_else(is.na(bbd_count), 0, bbd_count)) # Set missing bbd_count values to 0

# Print the result

print(wide_df_nb4)

colnames(wide_df_nb4)

# Add per hectare values of bbd count

wide_df_nb5 <- wide_df_nb4 %>%

mutate(pct_dens_bbd = case_when(

magp_site_id %in% c("30450865", "30450867", "30450871",

"30450874", "30452315", "30452316",

"30452331", "30452332", "30452333",

"30452362", "30452501", "30452502",

"30452505", "30452507", "30452511",

"30452512" ) ~ ((bbd_count * 10 / 0.1) / (pct_density_total_stems_per_ha_FAGU.GRA * total_density_sum)) * 100,

TRUE ~ ((bbd_count * 25 / 0.04) / (pct_density_total_stems_per_ha_FAGU.GRA * total_density_sum)) * 100

))

nrow(wide_df_nb4)

nrow(wide_df_nb5)

# Filter the dataset based on site id

sm_nb <- wide_df_nb5 %>%

filter(magp_site_id %in% site_ids)

# Filter again by plot type to exclude L2

sm_nb2 <- sm_nb %>%

filter(plot_type_id %in% c('L1', 'S1'))

# Filter again by treatment to keep CLR and CON

# Only a handful of sugar maple sites in CLR, none have treatment_pct

sm_nb3 <- sm_nb2 %>%

filter(treatment_type %in% c('CLR', 'CON'))

# Filter again by meas_num

sm_nb4<- sm_nb3%>%

filter(meas_num %in% c(0, 1))

# Export file along with French version (_fr)

file_path <- "C:/Users/martigau/OneDrive - NRCan RNCan/Documents/Personnel/Travail/EcolEvol/NFI Data/NB_CLI_NoHolderId_16/sm_nb4.csv"

write.csv(sm_nb4, file = file_path)

write.csv2(sm_nb4, "sm_nb4_fr.csv", row.names = FALSE)

### NEEDS FURTHER COMPILATION

### SEE R FILE 5_Combine_all_stats

FILE 4) COMPILE NFI DATA

##########################################

#### COMPILE DATA FOR NFI GROUND PLOTS ###

##########################################

#IMPORT NFI GROUND PLOT DATA FROM ALL THREE PROVINCES

library(data.table)

library(dplyr)

library(sf)

library(tidyr)

library(ggplot2)

library(geosphere)

library(parallel)

library(FNN)

library(RcppAnnoy)

library(knitr)

library(broom)

# Replace with the path to your CSV file

nfi_qc <- fread("C:/Users/martigau/OneDrive - NRCan RNCan/Documents/Personnel/Travail/EcolEvol/NFI Data/NFI_GPs/QC/magp_trees.csv")

nfi_on <- fread("C:/Users/martigau/OneDrive - NRCan RNCan/Documents/Personnel/Travail/EcolEvol/NFI Data/NFI_GPs/ON/magp_trees.csv")

nfi_nb <- fread("C:/Users/martigau/OneDrive - NRCan RNCan/Documents/Personnel/Travail/EcolEvol/NFI Data/NFI_GPs/NB/magp_trees.csv")

nfi_head_qc <- fread("C:/Users/martigau/OneDrive - NRCan RNCan/Documents/Personnel/Travail/EcolEvol/NFI Data/NFI_GPs/QC/magp_tree_header.csv")

nfi_head_on <- fread("C:/Users/martigau/OneDrive - NRCan RNCan/Documents/Personnel/Travail/EcolEvol/NFI Data/NFI_GPs/ON/magp_tree_header.csv")

nfi_head_nb <- fread("C:/Users/martigau/OneDrive - NRCan RNCan/Documents/Personnel/Travail/EcolEvol/NFI Data/NFI_GPs/NB/magp_tree_header.csv")

# Ensure numeric columns are treated as numeric

nfi_qc$dbh <- as.numeric(nfi_qc$dbh)

nfi_head_qc$meas_plot_size <- as.numeric(nfi_head_qc$meas_plot_size)

nfi_head_qc$meas_year <- as.numeric(nfi_head_qc$meas_year)

nfi_on$dbh <- as.numeric(nfi_on$dbh)

nfi_head_on$meas_plot_size <- as.numeric(nfi_head_on$meas_plot_size)

nfi_head_on$meas_year <- as.numeric(nfi_head_on$meas_year)

nfi_nb$dbh <- as.numeric(nfi_nb$dbh)

nfi_head_nb$meas_plot_size <- as.numeric(nfi_head_nb$meas_plot_size)

nfi_head_nb$meas_year <- as.numeric(nfi_head_nb$meas_year)

# Merge based on magp_site_id, plot_type_id, and meas_num

merged_nfi_qc <- merge(nfi_qc, nfi_head_qc, by = c("magp_site_id", "plot_type_id", "meas_num"))

merged_nfi_on <- merge(nfi_on, nfi_head_on, by = c("magp_site_id", "plot_type_id", "meas_num"))

merged_nfi_nb <- merge(nfi_nb, nfi_head_nb, by = c("magp_site_id", "plot_type_id", "meas_num"))

# Info on plot size

# Sugar maple (>=50% BA), meas_num 0, meas_year btw 1993-2017, TRT= CLR, CON

site_ids <- c("13013760", "11294361", "12806410", "11019911", "11019911", "13013210", "10950660", "12463510",

"9987660", "11019661", "13013010", "13013010", "11707511", "9850510", "9850510", "11225560",

"11225561", "12257110", "10950661", "11019910", "13082510", "13082510", "13082510", "11019660",

"12257710", "9781560", "13358110", "11363211", "12119360")

# QC: Six different plot sizes

merged_nfi_qc2 <- merged_nfi_qc %>%

filter(magp_site_id %in% site_ids)

unique(merged_nfi_qc2$meas_plot_size)

# ON: Two different plot sizes

merged_nfi_on2 <- merged_nfi_on %>%

filter(magp_site_id %in% site_ids)

unique(merged_nfi_on2$meas_plot_size)

# NB: Two different plot sizes

merged_nfi_nb2 <- merged_nfi_nb %>%

filter(magp_site_id %in% site_ids)

unique(merged_nfi_nb2$meas_plot_size)

# Dig deeper into NFI QC plots

unique_plot_sizes <- merged_nfi_qc2 %>%

group_by(plot_type_id) %>%

summarise(unique_meas_plot_size = unique(meas_plot_size))

# View the result

print(unique_plot_sizes)

# Count the number of plots for each plot size

plot_count_by_size <- merged_nfi_qc2 %>%

filter(magp_site_id %in% site_ids) %>% # Filter for the specified magp_site_ids

group_by(meas_plot_size) %>% # Group by meas_plot_size

summarise(plot_count = n(), .groups = "drop") # Count the number of plots for each plot size

# Print the result

print(plot_count_by_size)

# Specify the file path where you want to save the CSV file

file_path <- "C:/Users/martigau/OneDrive - NRCan RNCan/Documents/Personnel/Travail/EcolEvol/NFI Data/NFI_GPs/QC/merged_nfi_qc.csv"

write.csv(merged_nfi_qc, file = file_path)

# Specify the file path where you want to save the CSV file

file_path <- "C:/Users/martigau/OneDrive - NRCan RNCan/Documents/Personnel/Travail/EcolEvol/NFI Data/NFI_GPs/ON/merged_nfi_on.csv"

write.csv(merged_nfi_on, file = file_path)

# Specify the file path where you want to save the CSV file

file_path <- "C:/Users/martigau/OneDrive - NRCan RNCan/Documents/Personnel/Travail/EcolEvol/NFI Data/NFI_GPs/NB/merged_nfi_nb.csv"

write.csv(merged_nfi_nb, file = file_path)

# Calculate basal area (m^2/ha)

merged_nfi_qc$ba_ha <- (pi * (merged_nfi_qc$dbh / 200)^2) / merged_nfi_qc$meas_plot_size

write.csv(merged_nfi_qc, file = file_path)

merged_nfi_on$ba_ha <- (pi * (merged_nfi_on$dbh / 200)^2) / merged_nfi_on$meas_plot_size

write.csv(merged_nfi_on, file = file_path)

merged_nfi_nb$ba_ha <- (pi * (merged_nfi_nb$dbh / 200)^2) / merged_nfi_nb$meas_plot_size

write.csv(merged_nfi_nb, file = file_path)

# VIEW DATA

merged_nfi_qc

head(merged_nfi_qc, 10)

str(merged_nfi_qc)

names(merged_nfi_qc)

# KEEP ONLY THE LIVING TREES AND SAPLINGS

# KEEP ONLY THE LIVING TREES AND SAPLINGS WITH dbh > 1.0 FOR S1

live_nfi_qc <- merged_nfi_qc[

(merged_nfi_qc$tree_status %in% c('LF', 'LS', 'L', '-1')) &

(merged_nfi_qc$dbh > 1.0 | merged_nfi_qc$plot_type_id != 'S1'),

]

live_nfi_on <- merged_nfi_on[

(merged_nfi_on$tree_status %in% c('LF', 'LS', 'L', '-1')) &

(merged_nfi_on$dbh > 1.0 | merged_nfi_on$plot_type_id != 'S1'),

]

live_nfi_nb <- merged_nfi_nb[

(merged_nfi_nb$tree_status %in% c('LF', 'LS', 'L', '-1')) &

(merged_nfi_nb$dbh > 1.0 | merged_nfi_nb$plot_type_id != 'S1'),

]

# CALCULATE BASAL AREA FOR EACH TREE BY SITE, PLOT, MEAS_NUM, and SPECIES

library(dplyr)

# Sum basal area by experimental unit

ba_nfi_qc <- live_nfi_qc %>%

group_by(magp_site_id, plot_type_id, meas_num, species_gs, meas_year) %>%

summarize(total_basal_area_m2 = sum(ba_ha),

total_stems_per_ha = sum(stem_ha))

# Sum basal area by experimental unit

ba_nfi_on <- live_nfi_on %>%

group_by(magp_site_id, plot_type_id, meas_num, species_gs, meas_year) %>%

summarize(total_basal_area_m2 = sum(ba_ha),

total_stems_per_ha = sum(stem_ha))

# Sum basal area by experimental unit

ba_nfi_nb <- live_nfi_nb %>%

group_by(magp_site_id, plot_type_id, meas_num, species_gs, meas_year) %>%

summarize(total_basal_area_m2 = sum(ba_ha),

total_stems_per_ha = sum(stem_ha))

names(ba_nfi_qc)

# Keep specific columns in summary_data

ba_nfi_qc2 <- ba_nfi_qc %>%

select(magp_site_id, plot_type_id, meas_num, meas_year, species_gs, total_stems_per_ha, total_basal_area_m2)

ba_nfi_on2 <- ba_nfi_on %>%

select(magp_site_id, plot_type_id, meas_num, meas_year, species_gs, total_stems_per_ha, total_basal_area_m2)

ba_nfi_nb2 <- ba_nfi_nb %>%

select(magp_site_id, plot_type_id, meas_num, meas_year, species_gs, total_stems_per_ha, total_basal_area_m2)

# View the filtered summary data

print(ba_nfi_qc2)

# Export files

file_path <- "C:/Users/martigau/OneDrive - NRCan RNCan/Documents/Personnel/Travail/EcolEvol/NFI Data/NFI_GPs/QC/ba_nfi_qc2.csv"

write.csv(ba_nfi_qc2, file = file_path)

file_path <- "C:/Users/martigau/OneDrive - NRCan RNCan/Documents/Personnel/Travail/EcolEvol/NFI Data/NFI_GPs/ON/ba_nfi_on2.csv"

write.csv(ba_nfi_on2, file = file_path)

file_path <- "C:/Users/martigau/OneDrive - NRCan RNCan/Documents/Personnel/Travail/EcolEvol/NFI Data/NFI_GPs/NB/ba_nfi_nb2.csv"

write.csv(ba_nfi_nb2, file = file_path)

# Merge with 'magp_sites' to have latitude, longitude, and elevation

# QUEBEC

nfi_sites_qc <- fread("C:/Users/martigau/OneDrive - NRCan RNCan/Documents/Personnel/Travail/EcolEvol/NFI Data/NFI_GPs/QC/magp_sites.csv")

merged_nfi_sites_qc <- merge(ba_nfi_qc2, nfi_sites_qc, by = c("magp_site_id"), all.x = TRUE)

# ONTARIO

nfi_sites_on <- fread("C:/Users/martigau/OneDrive - NRCan RNCan/Documents/Personnel/Travail/EcolEvol/NFI Data/NFI_GPs/ON/magp_sites.csv")

merged_nfi_sites_on <- merge(ba_nfi_on2, nfi_sites_on, by = c("magp_site_id"), all.x = TRUE)

# NEW BRUNSWICK

nfi_sites_nb <- fread("C:/Users/martigau/OneDrive - NRCan RNCan/Documents/Personnel/Travail/EcolEvol/NFI Data/NFI_GPs/NB/magp_sites.csv")

merged_nfi_sites_nb <- merge(ba_nfi_nb2, nfi_sites_nb, by = c("magp_site_id"), all.x = TRUE)

# MERGE WITH TREATMENT - QUÉBEC

nfi_treat_qc <- fread("C:/Users/martigau/OneDrive - NRCan RNCan/Documents/Personnel/Travail/EcolEvol/NFI Data/NFI_GPs/QC/magp_treatment.csv")

nfi_treat_qc2 <- nfi_treat_qc %>%

select(magp_site_id, treatment_type, treatment_year, treatment_month, treatment_pct)

# Left join to keep control plots

nfi_site_treat_qc <- merge(merged_nfi_sites_qc, nfi_treat_qc2, by = c("magp_site_id"), all.x = TRUE)

# Replace NA values in treatment_type with "Control"

nfi_site_treat_qc$treatment_type[is.na(nfi_site_treat_qc$treatment_type)] <- "CON"

# Check the resulting data

head(nfi_site_treat_qc)

# Specify the file path where you want to save the CSV file

file_path <- "C:/Users/martigau/OneDrive - NRCan RNCan/Documents/Personnel/Travail/EcolEvol/NFI Data/NFI_GPs/QC/nfi_site_treat_qc.csv"

write.csv(nfi_site_treat_qc, file = file_path)

write.csv2(nfi_site_treat_qc, "nfi_site_treat_qc_fr.csv", row.names = FALSE)

# MERGE WITH TREATMENT - ONTARIO

nfi_treat_on <- fread("C:/Users/martigau/OneDrive - NRCan RNCan/Documents/Personnel/Travail/EcolEvol/NFI Data/NFI_GPs/ON/magp_treatment.csv")

nfi_treat_on2 <- nfi_treat_on %>%

select(magp_site_id, treatment_type, treatment_year, treatment_month, treatment_pct)

# Left join to keep control plots

nfi_site_treat_on <- merge(merged_nfi_sites_on, nfi_treat_on2, by = c("magp_site_id"), all.x = TRUE)

# Replace NA values in treatment_type with "Control"

nfi_site_treat_on$treatment_type[is.na(nfi_site_treat_on$treatment_type)] <- "CON"

# Check the resulting data

head(nfi_site_treat_on)

# Specify the file path where you want to save the CSV file

file_path <- "C:/Users/martigau/OneDrive - NRCan RNCan/Documents/Personnel/Travail/EcolEvol/NFI Data/NFI_GPs/ON/nfi_site_treat_on.csv"

write.csv(nfi_site_treat_on, file = file_path)

write.csv2(nfi_site_treat_on, "nfi_site_treat_on_fr.csv", row.names = FALSE)

# MERGE WITH TREATMENT - NEW BRUNSWICK

nfi_treat_nb <- fread("C:/Users/martigau/OneDrive - NRCan RNCan/Documents/Personnel/Travail/EcolEvol/NFI Data/NFI_GPs/NB/magp_treatment.csv")

nfi_treat_nb2 <- nfi_treat_nb %>%

select(magp_site_id, treatment_type, treatment_year, treatment_month, treatment_pct)

# Left join to keep control plots

nfi_site_treat_nb <- merge(merged_nfi_sites_nb, nfi_treat_nb2, by = c("magp_site_id"), all.x = TRUE)

# Replace NA values in treatment_type with "Control"

nfi_site_treat_nb$treatment_type[is.na(nfi_site_treat_nb$treatment_type)] <- "CON"

# Check the resulting data

head(nfi_site_treat_nb)

# Specify the file path where you want to save the CSV file

file_path <- "C:/Users/martigau/OneDrive - NRCan RNCan/Documents/Personnel/Travail/EcolEvol/NFI Data/NFI_GPs/NB/nfi_site_treat_nb.csv"

write.csv(nfi_site_treat_nb, file = file_path)

write.csv2(nfi_site_treat_nb, "nfi_site_treat_nb_fr.csv", row.names = FALSE)

##########################################################

### COMBINE NFI GROUND PLOTS FROM ALL THREE PROVINCES ###

##########################################################

# Combine the datasets by rows

nfi_all <- rbind(nfi_site_treat_qc, nfi_site_treat_on, nfi_site_treat_nb)

# Specify the file path where you want to save the CSV file

file_path <- "C:/Users/martigau/OneDrive - NRCan RNCan/Documents/Personnel/Travail/EcolEvol/NFI Data/NFI_GPs/nfi_all.csv"

write.csv(nfi_all, file = file_path)

write.csv2(nfi_all, "nfi_all_fr.csv", row.names = FALSE)

#### PIVOT TABLE TIME - PUT SPECIES IN EACH COLUMN

#Pivot the data from long format to wide format, where species are columns

library(dplyr)

library(tidyr)

wide_nfi <- nfi_all %>%

pivot_wider(names_from = species_gs,

values_from = c(total_stems_per_ha, total_basal_area_m2))

# Replace NA with 0

wide_nfi2 <- wide_nfi %>%

mutate(across(starts_with("total_stems_per_ha_"), ~replace(., is.na(.), 0)),

across(starts_with("total_basal_area_m2_"), ~replace(., is.na(.), 0)))

# Print the result

print(wide_nfi2)

# Export file wide_df_nfi2

file_path <- "C:/Users/martigau/OneDrive - NRCan RNCan/Documents/Personnel/Travail/EcolEvol/NFI Data/NFI_GPs/wide_nfi2.csv"

write.csv(wide_nfi2, file = file_path)

write.csv2(wide_nfi2, "wide_nfi2_fr.csv", row.names = FALSE)

# IDENTIFY UNIQUE SPECIES LIST FOR THE THREE PROVINCES, n = 84

unique_species_nfi <- unique(nfi_all$species_gs)

# Print the unique species

print(unique_species_nfi)

#Save as .csv file

file_path <- "C:/Users/martigau/OneDrive - NRCan RNCan/Documents/Personnel/Travail/EcolEvol/NFI Data/NFI_GPs/unique_species_nfi.csv"

write.csv(unique_species_nfi, file = file_path)

### ADD DENSITY AND BASAL AREA TOTALS

library(dplyr)

library(tidyr)

# List of species from your unique list

species_list_nfi <- c("ABIE.BAL", "BETU.PAP", "PICE.GLA", "PICE.MAR", "SALI.HUM", "LARI.LAR", "UNKN.SPP", "BETU.GLA", "ACER.RUB",

"POPU.TRE", "ACER.SPI", "ALNU.CRI", "NEMO.MUC", "ALNU.INC", "CORN.STO", "SORB.AME", "AMEL.BAR", "ILEX.MUC",

"SORB.DEC", "AMEL.SPP", "PRUN.PEN", "PINU.BAN", "SALI.BEB", "SALI.DIS", "ALNU.VIR", "LEDU.GRO", "SALI.LUC",

"BETU.ALL", "ACER.PEN", "ACER.SAH", "CORY.COR", "FRAX.NIG", "THUJ.OCC", "PICE.RUB", "TAXU.CAN", "POPU.BAL",

"TSUG.CAN", "VIBU.LAN", "BETU.POP", "PINU.STR", "TILI.AME", "FAGU.GRA", "SALI.SPP", "PICE.ABI", "SAMB.RAC",

"AMEL.LAE", "PRUN.VIR", "PRUN.SER", "FRAX.AME", "AMEL.SAN", "QUER.RUB", "POPU.GRA", "OSTR.VIR", "LONI.CAN",

"CARP.CAR", "CARY.COR", "FRAX.PEN", "ULMU.AME", "ULMU.RUB", "SALI.MAC", "SALI.PEN", "QUER.MAC", "GENH.SPP",

"RHAM.CAT", "RHAM.FRA", "PINU.RES", "SORB.SPP", "PRUN.SPP", "CRAT.PUN", "CRAT.SPP", "CARY.OVA", "QUER.ALB",

"JUNI.VIR", "ACER.SPP", "ACER.NEG", "MALU.SPP", "ACER.SAC", "CRAT.CHR", "RHAM.SPP", "ACER.FRE", "JUGL.NIG",

"ULMU.THO", "POPU.DEL", "VIBU.SPP")

# Generate column names for the species in the list

stem_columns <- paste0("total_stems_per_ha_", species_list_nfi)

basal_area_columns <- paste0("total_basal_area_m2_", species_list_nfi)

# Ensure the columns exist in your dataframe

stem_columns <- intersect(stem_columns, names(wide_nfi2))

basal_area_columns <- intersect(basal_area_columns, names(wide_nfi2))

# Perform the calculations

wide_nfi3 <- wide_nfi2 %>%

rowwise() %>%

mutate(

total_density = sum(c_across(all_of(stem_columns)), na.rm = TRUE),

total_basal_area = sum(c_across(all_of(basal_area_columns)), na.rm = TRUE)

) %>%

ungroup() %>%

mutate(

# Create percentage columns for density

across(

starts_with("total_stems_per_ha_"),

~ . / (total_density + 1e-6) * 100,

.names = "pct_density_{col}"

),

# Create percentage columns for basal area

across(

starts_with("total_basal_area_m2_"),

~ . / (total_basal_area + 1e-6) * 100,

.names = "pct_basal_area_{col}"

)

) %>%

# Add columns for total sums

mutate(

total_density_sum = total_density,

total_basal_area_sum = total_basal_area

)

# Extract percentage columns

percentage_columns <- names(wide_nfi3) %>%

.[grepl("pct_density_|pct_basal_area_", .)]

# Columns to keep from the original dataframe

additional_columns <- c(

"magp_site_id", "plot_type_id", "meas_num", "meas_year", "V1",

"province", "latitude", "longitude", "elevation", "aspect_class",

"slope_class", "slope_position", "gpp_type", "sources", "treatment_type",

"treatment_year", "treatment_month", "treatment_pct"

)

# Ensure the additional columns are in the dataframe

additional_columns <- intersect(additional_columns, names(wide_nfi2))

# Create the final dataframe with percentage columns, additional columns, and total sum columns

wide_nfi3_final <- wide_nfi3 %>%

select(all_of(additional_columns), all_of(percentage_columns), total_density_sum, total_basal_area_sum)

# Save the modified data frame to CSV

write.csv(wide_nfi3_final, file = "C:/Users/martigau/OneDrive - NRCan RNCan/Documents/Personnel/Travail/EcolEvol/NFI Data/ON_PSPPGP/wide_df_on3.csv", row.names = FALSE)

write.csv2(wide_nfi3_final, file = "C:/Users/martigau/OneDrive - NRCan RNCan/Documents/Personnel/Travail/EcolEvol/NFI Data/ON_PSPPGP/wide_df_on3_fr.csv", row.names = FALSE)

# Inspect the final dataframe to ensure the columns are included

cat("Columns in wide_nfi3_final:\n")

print(names(wide_nfi3_final))

# Step 1: Check for duplicate column names

anyDuplicated(names(wide_nfi3_final))

# Ensure column names are unique

names(wide_nfi3_final) <- make.unique(names(wide_nfi3_final))

#####################################

# ADD DISEASED STEM COUNT FOR BBD ###

#####################################

# Import tree condition data - Replace with the path to your own CSV file

tree_condition_nfi_nb <- fread("C:/Users/martigau/OneDrive - NRCan RNCan/Documents/Personnel/Travail/EcolEvol/NFI Data/NFI_GPs/NB/magp_tree_condition.csv")

# Import tree condition data - Replace with the path to your own CSV file

tree_condition_nfi_on <- fread("C:/Users/martigau/OneDrive - NRCan RNCan/Documents/Personnel/Travail/EcolEvol/NFI Data/NFI_GPs/ON/magp_tree_condition.csv")

# Merge with 'tree_condition' to have the number of trees with cankers, cracked bark due to BBD

bbd_nfi_nb <- tree_condition_nfi_nb %>%

filter(condition == "DS") %>%

group_by(magp_site_id, plot_type_id, meas_num) %>%

summarise(count = n(), .groups = "drop") %>%

rename(bbd_count = count) # Renaming the count column to bbd_count

# Merge with 'tree_condition' to have the number of trees with cankers, cracked bark due to BBD

bbd_nfi_on <- tree_condition_nfi_on %>%

filter(condition == "DS") %>%

group_by(magp_site_id, plot_type_id, meas_num) %>%

summarise(count = n(), .groups = "drop") %>%

rename(bbd_count = count) # Renaming the count column to bbd_count

# Merge with original data frame to set bbd_count = 0 where missing

wide_nfi4_final <- wide_nfi3_final %>%

left_join(bbd_nfi_nb, bbd_nfi_on, by = c("magp_site_id", "plot_type_id", "meas_num")) %>%

mutate(bbd_count = if_else(is.na(bbd_count), 0, bbd_count)) # Set missing bbd_count values to 0

# Print the result

print(wide_nfi4_final)

colnames(wide_nfi4_final)

#Calculate density/ha and % density of FAGU.GRA

wide_nfi5_final <- wide_nfi4_final %>%

mutate(

pct_dens_bbd = bbd_count * 25 * (pct_density_total_stems_per_ha_FAGU.GRA / 100))

print(wide_nfi5_final)

unique(wide_nfi5_final$pct_dens_bbd)

# Step 2: Filter (n = 29)

# Sugar maple (>=50% BA), meas_num 0, meas_year btw 1993-2017, TRT= CLR, CON

site_ids <- c("13013760", "11294361", "12806410", "11019911", "11019911", "13013210", "10950660", "12463510",

"9987660", "11019661", "13013010", "13013010", "11707511", "9850510", "9850510", "11225560",

"11225561", "12257110", "10950661", "11019910", "13082510", "13082510", "13082510", "11019660",

"12257710", "9781560", "13358110", "11363211", "12119360")

# Filter the dataset based on site id

sm_nfi <- wide_nfi5_final %>%

filter(magp_site_id %in% site_ids)

str(sm_nfi)

# Filter again by plot type to exclude L2

sm_nfi2 <- sm_nfi %>%

filter(plot_type_id %in% c('L1', 'S1'))

str(sm_nfi2)

# Filter again by treatment to keep CLR and CON

sm_nfi3 <- sm_nfi2 %>%

filter(treatment_type %in% c('CLR', 'CON'))

str(sm_nfi3)

# Filter again by meas_num

sm_nfi4<- sm_nfi3%>%

filter(meas_num %in% c(0, 1, 2))

str(sm_nfi4)

colnames(sm_nfi4)

# Add a column named 'sources' with the value 'nfi' to the dataframe

sm_nfi4 <- sm_nfi4 %>%

mutate(sources = "nfi")

# Inspect the updated dataframe to confirm the new column

print(head(sm_nfi4))

# Export file along with French version (_fr)

file_path <- "C:/Users/martigau/OneDrive - NRCan RNCan/Documents/Personnel/Travail/EcolEvol/NFI Data/NFI_GPs/sm_nfi4.csv"

write.csv(sm_nfi4, file = file_path)

write.csv2(sm_nfi4, "sm_nfi4_fr.csv", row.names = FALSE)

### NEEDS FURTHER COMPILATION

### SEE R FILE 5_Combine_all_stats

FILE 5) COMBINE ALL STATS

###################################

### COMBINE ALL FOUR DATASETS ####

###################################

installed.packages()

install.packages("data.table")

install.packages("dplyr")

install.packages("tidyr")

install.packages("sf")

install.packages("ggplot2")

install.packages("knitr")

install.packages("broom")

install.packages("raster")

install.packages("climate")

library(data.table)

library(dplyr)

library(sf)

library(tidyr)

library(ggplot2)

library(knitr)

library(broom)

library(raster)

library(climate)

update.packages()

# Use your own path to import datasets from QC, ON, NB, and NFI

sm4 <- fread("C:/Users/martigau/OneDrive - NRCan RNCan/Documents/Personnel/Travail/EcolEvol/NFI Data/QC_PSP/sm4.csv")

sm_on4 <- fread("C:/Users/martigau/OneDrive - NRCan RNCan/Documents/Personnel/Travail/EcolEvol/NFI Data/ON_PSPPGP/sm_on4.csv")

sm_nb4 <- fread("C:/Users/martigau/OneDrive - NRCan RNCan/Documents/Personnel/Travail/EcolEvol/NFI Data/NB_CLI_NoHolderId_16/sm_nb4.csv")

sm_nfi4 <- fread("C:/Users/martigau/OneDrive - NRCan RNCan/Documents/Personnel/Travail/EcolEvol/NFI Data/NFI_GPs/sm_nfi4.csv")

colnames(sm4)

colnames(sm_on4)

colnames(sm_nb4)

colnames(sm_nfi4)

unique(sm_on4$magp_site_id)

# Rename multiple columns to standardize

setnames(sm4,

old = c("total_density",

"total_basal_area",

"pct_density_total_stems_per_ha_ACER.SAH",

"pct_density_total_stems_per_ha_FAGU.GRA",

"pct_density_total_stems_per_ha_BETU.ALL",

"pct_basal_area_total_basal_area_m2_ACER.SAH",

"pct_basal_area_total_basal_area_m2_FAGU.GRA",

"pct_basal_area_total_basal_area_m2_BETU.ALL"),

new = c("total_density_sum",

"total_basal_area_sum",

"pct_density_ACER.SAH",

"pct_density_FAGU.GRA",

"pct_density_BETU.ALL",

"pct_basal_area_ACER.SAH",

"pct_basal_area_FAGU.GRA",

"pct_basal_area_BETU.ALL"))

# Keep specific columns in summary_data

sm_que <- sm4 %>%

dplyr::select(

magp_site_id, plot_type_id, meas_num, meas_year, province, latitude,

longitude, elevation, aspect_class, slope_class, slope_position,

sources, treatment_type, treatment_year, treatment_pct,

total_density_sum, total_basal_area_sum, bbd_count, pct_dens_bbd,

pct_density_ACER.SAH,

pct_density_FAGU.GRA,

pct_density_BETU.ALL,

pct_basal_area_ACER.SAH,

pct_basal_area_FAGU.GRA,

pct_basal_area_BETU.ALL)

colnames(sm_que)

print(sm_que)

#Add absolute density and basal area for sugar maple and beech

sm_que2 <- sm_que %>%

mutate(

density_ACER.SAH = pct_density_ACER.SAH * total_density_sum/100,

ba_ACER.SAH = pct_basal_area_ACER.SAH * total_basal_area_sum/100,

density_FAGU.GRA = pct_density_FAGU.GRA * total_density_sum/100,

ba_FAGU.GRA = pct_basal_area_FAGU.GRA * total_basal_area_sum/100

)

# Rename multiple columns to standardize Ontario dataset

library(data.table)

setnames(sm_on4,

old = c("plot_type_id2", "pct_density_total_stems_per_ha_ACER.SAH",

"pct_density_total_stems_per_ha_FAGU.GRA",

"pct_density_total_stems_per_ha_BETU.ALL"),

new = c("plot_type_id", "pct_density_ACER.SAH", "pct_density_FAGU.GRA", "pct_density_BETU.ALL"))

colnames(sm_on4)

# Keep specific columns in summary_data

sm_ont <- sm_on4 %>%

dplyr::select(

magp_site_id, plot_type_id, meas_num, meas_year, province, latitude,

longitude, elevation, aspect_class, slope_class, slope_position,

sources, treatment_type, treatment_year, treatment_pct,

total_density_sum, total_basal_area_sum, bbd_count, pct_dens_bbd,

pct_density_ACER.SAH, pct_density_FAGU.GRA, pct_density_BETU.ALL,

pct_basal_area_ACER.SAH, pct_basal_area_FAGU.GRA, pct_basal_area_BETU.ALL)

colnames(sm_ont)

#Add absolute density and basal area for sugar maple and beech

sm_ont2 <- sm_ont %>%

mutate (

density_ACER.SAH = pct_density_ACER.SAH * total_density_sum/100,

ba_ACER.SAH = pct_basal_area_ACER.SAH * total_basal_area_sum/100,

density_FAGU.GRA = pct_density_FAGU.GRA * total_density_sum/100,

ba_FAGU.GRA = pct_basal_area_FAGU.GRA * total_basal_area_sum/100

)

# Keep specific columns in summary_data

setnames(sm_nb4,

old = c("pct_density_total_stems_per_ha_ACER.SAH",

"pct_density_total_stems_per_ha_FAGU.GRA",

"pct_density_total_stems_per_ha_BETU.ALL",

"pct_basal_area_total_basal_area_m2_ACER.SAH",

"pct_basal_area_total_basal_area_m2_FAGU.GRA",

"pct_basal_area_total_basal_area_m2_BETU.ALL"

),

new = c("pct_density_ACER.SAH",

"pct_density_FAGU.GRA",

"pct_density_BETU.ALL",

"pct_basal_area_ACER.SAH",

"pct_basal_area_FAGU.GRA",

"pct_basal_area_BETU.ALL"))

colnames(sm_nb4)

sm_nbr <- sm_nb4 %>%

dplyr::select(

magp_site_id, plot_type_id, meas_num, meas_year, province, latitude,

longitude, elevation, aspect_class, slope_class, slope_position,

sources, treatment_type, treatment_year, treatment_pct,

total_density_sum, total_basal_area_sum, bbd_count, pct_dens_bbd,

pct_density_ACER.SAH, pct_density_FAGU.GRA, pct_density_BETU.ALL,

pct_basal_area_ACER.SAH, pct_basal_area_FAGU.GRA, pct_basal_area_BETU.ALL)

colnames(sm_nbr)

#Add absolute density and basal area for sugar maple and beech

sm_nbr2 <- sm_nbr %>%

mutate(

density_ACER.SAH = pct_density_ACER.SAH * total_density_sum/100,

ba_ACER.SAH = pct_basal_area_ACER.SAH * total_basal_area_sum/100,

density_FAGU.GRA = pct_density_FAGU.GRA * total_density_sum/100,

ba_FAGU.GRA = pct_basal_area_FAGU.GRA * total_basal_area_sum/100

)

# Keep specific columns in summary_data

setnames(sm_nfi4,

old = c("pct_density_total_stems_per_ha_ACER.SAH",

"pct_density_total_stems_per_ha_FAGU.GRA",

"pct_density_total_stems_per_ha_BETU.ALL",

"pct_basal_area_total_basal_area_m2_ACER.SAH",

"pct_basal_area_total_basal_area_m2_FAGU.GRA",

"pct_basal_area_total_basal_area_m2_BETU.ALL"

),

new = c("pct_density_ACER.SAH",

"pct_density_FAGU.GRA",

"pct_density_BETU.ALL",

"pct_basal_area_ACER.SAH",

"pct_basal_area_FAGU.GRA",

"pct_basal_area_BETU.ALL"))

colnames(sm_nfi4)

sm_nfi <- sm_nfi4 %>%

dplyr::select(

magp_site_id, plot_type_id, meas_num, meas_year, province, latitude,

longitude, elevation, aspect_class, slope_class, slope_position,

sources, treatment_type, treatment_year, treatment_pct,

total_density_sum, total_basal_area_sum,

pct_density_ACER.SAH, pct_density_FAGU.GRA, pct_density_BETU.ALL,

pct_basal_area_ACER.SAH, pct_basal_area_FAGU.GRA, pct_basal_area_BETU.ALL)

colnames(sm_nfi)

#Add absolute density and basal area for sugar maple and beech

sm_nfi2 <- sm_nfi %>%

mutate(

density_ACER.SAH = pct_density_ACER.SAH * total_density_sum/100,

ba_ACER.SAH = pct_basal_area_ACER.SAH * total_basal_area_sum/100,

density_FAGU.GRA = pct_density_FAGU.GRA * total_density_sum/100,

ba_FAGU.GRA = pct_basal_area_FAGU.GRA * total_basal_area_sum/100

)

###################################################

############ COMBINE ALL OF THE DATASETS #########

###################################################

# Combine the data frames by row

sm_all <- rbind(sm_que2, sm_ont2, sm_nbr2, sm_nfi2, fill = TRUE)

#Delete 'treatment_type' = "U" in Ontario, unknown

sm_all2 <- sm_all[sm_all$treatment_type != "U", ]

# Specify the file path where you want to save the CSV file

file_path <- "C:/Users/martigau/OneDrive - NRCan RNCan/Documents/Personnel/Travail/EcolEvol/NFI Data/sm_all2.csv"

write.csv(sm_all2, file = file_path)

write.csv2(sm_all2, "sm_all2_fr.csv", row.names = FALSE)

unique(sm_all2$magp_site_id)

# Open climate data

climate_data <- fread("C:/Users/martigau/OneDrive - NRCan RNCan/Documents/Personnel/Travail/EcolEvol/NFI Data/sm_all3_climate.csv")

nrow(climate_data)

colnames(climate_data)

# Filter out duplicates to keep rows with the lowest treatment_year for each combination of magp_site_id, plot_type_id, and decade

sm_all2_unique <- sm_all2 %>%

arrange(magp_site_id, plot_type_id, meas_year, treatment_year) %>% # Sort by treatment_year within each group

group_by(magp_site_id, plot_type_id, meas_year) %>% # Group by unique combinations

slice_min(order_by = treatment_year, n = 1) %>% # Keep the row with the lowest treatment_year

ungroup() # Remove grouping

nrow(sm_all2)

nrow(sm_all2_unique)

print(sm_all2_unique)

# Specify the file path where you want to save the CSV file

file_path <- "C:/Users/martigau/OneDrive - NRCan RNCan/Documents/Personnel/Travail/EcolEvol/NFI Data/sm_all2_unique.csv"

write.csv(sm_all2_unique, file = file_path)

write.csv2(sm_all2_unique, "sm_all2_unique_fr.csv", row.names = FALSE)

print(sm_all2_unique)

unique(sm_all2_unique$pct_dens_bbd)

unique(sm_all2_unique$magp_site_id)

##############################

### CLIMATE DATA ADDITION ###

### BASED ON DAILY DATA ###

### BIOSIM VERSION 11 ###

##############################

# Load dataset created using BioSIM version 11

export_climate <- fread("C:/Users/martigau/OneDrive - NRCan RNCan/Documents/Personnel/Travail/EcolEvol/NFI Data/export_climate.csv")

colnames(export_climate)

# Rename 'Name' to 'magp_site_id'

export_climate <- export_climate %>%

rename(magp_site_id = Name)

colnames(export_climate)

# Keep variables of interest

export_climate2 <- export_climate %>%

dplyr::select("magp_site_id", "Latitude", "Longitude", "Elevation", "Year", "Month",

"Day", "Minimum Air Temperature", "Maximum Air Temperature",

"Air Temperature", "Total Precipitation",

"Atmospheric Pressure", "Snow Precipitation",

"Snow Depth Accumulation", "Snow Water Equivalent")

colnames(export_climate2)

str(export_climate2)

# Step 1: Calculate monthly means (temperature) or sums (precipitation) for each site

monthly_data <- export_climate2 %>%

group_by(magp_site_id, Year, Month) %>%

summarise(

monthly_temp = mean(`Air Temperature`, na.rm = TRUE), # Monthly average temperature

monthly_temp_min = mean(`Minimum Air Temperature`, na.rm = TRUE), # Monthly min temp

monthly_temp_max = mean(`Maximum Air Temperature`, na.rm = TRUE), # Monthly max temp

monthly_precip = sum(`Total Precipitation`, na.rm = TRUE), # Monthly total precipitation

monthly_snow = max(`Snow Depth Accumulation`, na.rm = TRUE), # Monthly snow depth accumulation

monthly_vapr = mean(`Atmospheric Pressure`, na.rm = TRUE) # Monthly mean vapr

)

print(monthly_data)

str(monthly_data)

# Step 1.5: Calculate summer temperature (June, July, August) and summer precipitation for each site

summer_data <- monthly_data %>%

filter(Month %in% c(6, 7, 8)) %>% # Filter for June (6), July (7), and August (8)

group_by(magp_site_id, Year) %>% # Group by site and year

summarise(

summer_temp = mean(monthly_temp, na.rm = TRUE), # Average summer temperature

summer_temp_min = mean(monthly_temp_min, na.rm = TRUE), # Average min summer temperature

summer_temp_max = mean(monthly_temp_max, na.rm = TRUE), # Average max summer temperature

summer_precip = sum(monthly_precip, na.rm = TRUE) # Total summer precipitation

)

print(summer_data)

# Step 2: Calculate annual means (temperature) or sums (precipitation) for each site

annual_data <- monthly_data %>%

group_by(magp_site_id, Year) %>%

summarise(

annual_temp = mean(monthly_temp, na.rm = TRUE), # Annual average temperature

annual_temp_min = mean(monthly_temp_min, na.rm = TRUE), # Annual min average temperature

annual_temp_max = mean(monthly_temp_max, na.rm = TRUE), # Annual max average temperature

annual_precip = sum(monthly_precip, na.rm = TRUE), # Annual total precipitation

annual_snow = sum(monthly_snow, na.rm = TRUE), # Annual snow depth accumulation

annual_vapr = mean(monthly_vapr, na.rm = TRUE) # Annual average vapr

)

print(annual_data)

# Step 3: Join summer_data with the annual_data in step 2

annual_data_with_summer <- annual_data %>%

left_join(summer_data, by = c("magp_site_id", "Year"))

print(annual_data_with_summer)

# Specify the file path where you want to save the CSV file

file_path <- "C:/Users/martigau/OneDrive - NRCan RNCan/Documents/Personnel/Travail/EcolEvol/NFI Data/annual_data_with_summer.csv"

write.csv(annual_data_with_summer, file = file_path)

write.csv2(annual_data_with_summer, "annual_data_with_summer_fr.csv", row.names = FALSE)

# Step 4: Calculate 40-year averages for each site (mean of the annual values)

climate_data <- annual_data_with_summer %>%

group_by(magp_site_id) %>%

summarise(

mean_temp_40_years = mean(annual_temp, na.rm = TRUE), # 40-year mean temperature

mean_temp_40_years_min = mean(annual_temp_min, na.rm = TRUE), # 40-year min temperature

mean_temp_40_years_max = mean(annual_temp_max, na.rm = TRUE), # 40-year max temperature

mean_vapr_40_years = mean(annual_vapr, na.rm = TRUE), # 40-year mean vapr

summer_temp_40_years = mean(summer_temp, na.rm = TRUE), # 40-year mean summer temp

total_precip_40_years = mean(annual_precip, na.rm = TRUE), # 40-year total precipitation

summer_precip_40_years = mean(summer_precip, na.rm = TRUE), #40-year mean summer precip

total_snow_40_years = mean(annual_snow, na.rm = TRUE) # 40-year mean snow depth accumulation

)

# View the result

print(climate_data, n = 390)

str(climate_data)

# Specify the file path where you want to save the CSV file

file_path <- "C:/Users/martigau/OneDrive - NRCan RNCan/Documents/Personnel/Travail/EcolEvol/NFI Data/climate_data.csv"

write.csv(climate_data, file = file_path)

write.csv2(climate_data, "climate_data_fr.csv", row.names = FALSE)

# Calculate the min, mean, and max for all sites combined

climate_stats <- climate_data %>%

summarise(

min_temp_40_years = min(mean_temp_40_years, na.rm = TRUE), # Minimum temperature

mean_temp_40_years = mean(mean_temp_40_years, na.rm = TRUE), # Mean temperature

max_temp_40_years = max(mean_temp_40_years, na.rm = TRUE), # Maximum temperature

min_summer_temp_40_years = min(summer_temp_40_years, na.rm = TRUE), # Minimum temperature

mean_summer_temp_40_years = mean(summer_temp_40_years, na.rm = TRUE), # Mean temperature

max_summer_temp_40_years = max(summer_temp_40_years, na.rm = TRUE), # Maximum temperature

min_precip_40_years = min(total_precip_40_years, na.rm = TRUE), # Minimum precipitation

mean_precip_40_years = mean(total_precip_40_years, na.rm = TRUE), # Mean precipitation

max_precip_40_years = max(total_precip_40_years, na.rm = TRUE), # Maximum precipitation

min_summer_precip_40_years = min(summer_precip_40_years, na.rm = TRUE), # Minimum temperature

mean_summer_precip_40_years = mean(summer_precip_40_years, na.rm = TRUE), # Mean temperature

max_summer_precip_40_years = max(summer_precip_40_years, na.rm = TRUE), # Maximum temperature

min_snow_40_years = min(total_snow_40_years, na.rm = TRUE),

mean_snow_40_years = mean(total_snow_40_years, na.rm = TRUE),

max_snow_40_years = max(total_snow_40_years, na.rm = TRUE),

min_vapr_40_years = min(mean_vapr_40_years, na.rm = TRUE),

mean_vapr_40_years = mean(mean_vapr_40_years, na.rm = TRUE),

max_vapr_40_years = max(mean_vapr_40_years, na.rm = TRUE)

)

# View the summary statistics

print(climate_stats)

str(climate_stats)

# Specify the file path where you want to save the CSV file

file_path <- "C:/Users/martigau/OneDrive - NRCan RNCan/Documents/Personnel/Travail/EcolEvol/NFI Data/climate_stats.csv"

write.csv(climate_stats, file = file_path)

write.csv2(climate_stats, "climate_stats_fr.csv", row.names = FALSE)

###################################################################

####### Exclude treatments without harvesting intensity ###########

###################################################################

#Load dataset

sm_all2_unique <- fread("C:/Users/martigau/OneDrive - NRCan RNCan/Documents/Personnel/Travail/EcolEvol/NFI Data/sm_all2_unique.csv")

colnames(sm_all2_unique)

unique(sm_all2_unique$treatment_type)

#List of magp_site_id values to exclude

exclude_ids <- c(30002050, 30002095, 30002114, 30002165, 30002280,

30002299, 30002358, 30002359, 30002822, 30002922, 30002923, 30003054,

30003355, 30006121, 30006625, 30006680,

30006702, 30006710, 30006711, 30007182)

#30001952y, 30001975y, 30001990y, 30001998y, 30002001y,

#30002047y, 30002051y,

#30002157y, 30002160y, 30002162y, 30002178y,

#30002202y, 30002219y, 30002220y, 30002260y, 30002261y,

#30002295y,

#30002414y, 30002416y, 30002823y, 30002920y,

#30002933y, 30002936y,

#30003583y, 30005127y, 30006032y,

#30006472y, 30006539y, 30006622y, 30006724y,

# Remove columns by name in-place

set(sm_all2_unique, j = "V1", value = NULL)

set(sm_all2_unique, j = "V1", value = NULL)

colnames(sm_all2_unique)

# Filter out the specified magp_site_id values

ecolevol <- sm_all2_unique %>%

filter(!magp_site_id %in% exclude_ids)

# Total 895 plots

unique(ecolevol$magp_site_id)

unique(ecolevol$treatment_type)

colnames(ecolevol)

## Save the table to a CSV file

file_path <- "C:/Users/martigau/OneDrive - NRCan RNCan/Documents/Personnel/Travail/EcolEvol/NFI Data/ecolevol.csv"

write.csv(ecolevol, file = file_path)

write.csv2(ecolevol, 'ecolevol_fr.csv', row.names = FALSE)

###############################################################

###############################################################

#Load datasets

ecolevol <- fread("C:/Users/martigau/OneDrive - NRCan RNCan/Documents/Personnel/Travail/EcolEvol/NFI Data/ecolevol.csv")

ecozones <- fread("C:/Users/martigau/OneDrive - NRCan RNCan/Documents/Personnel/Travail/EcolEvol/NFI Data/QC_PSP/magp_ecozones.csv")

colnames(ecolevol)

colnames(ecozones)

print(ecozones)

# Remove columns by name in-place

set(ecolevol, j = "V1", value = NULL)

set(ecolevol, j = "V1", value = NULL)

colnames(ecolevol)

# Merge file with ecozones

ecolevol2 <- merge(ecolevol, ecozones[, c("magp_site_id", "Name")], by = "magp_site_id", all.x = TRUE)

# View data

colnames(ecolevol2)

# Rename 'Name' to 'ecozone' using dplyr's rename() function

ecolevol3 <- ecolevol2 %>%

rename(ecozone = Name)

# View data

unique(ecolevol3$ecozone)

# Display rows where 'ecozone' is NA

na_rows <- ecolevol3[is.na(ecolevol3$ecozone), ]

# Rows without ecozone, but this gets sorted out later

print(na_rows)

nrow(na_rows)

# Step 1: Filter out sites with fewer than 2 'S1' measurements (as before)

sites_with_enough_S1 <- ecolevol3 %>%

filter(plot_type_id == 'S1') %>%

group_by(magp_site_id) %>%

summarise(s1_count = n_distinct(meas_year), .groups = 'drop') %>%

filter(s1_count >= 2) %>%

pull(magp_site_id) # Get the list of site IDs with enough 'S1' measurements

print(sites_with_enough_S1)

# Step 2: Filter the main dataset to include only sites with at least 2 'S1' measurements

ecolevol4 <- ecolevol3 %>%

filter(magp_site_id %in% sites_with_enough_S1)

nrow(ecolevol3)

nrow(ecolevol4)

# 895, 528

unique(ecolevol3$magp_site_id)

unique(ecolevol4$magp_site_id)

unique(ecolevol4$treatment_type)

## Save the table to a CSV file

file_path <- "C:/Users/martigau/OneDrive - NRCan RNCan/Documents/Personnel/Travail/EcolEvol/NFI Data/ecolevol4.csv"

write.csv(ecolevol4, file = file_path)

write.csv2(ecolevol4, 'ecolevol4_fr.csv', row.names = FALSE)

# Filter out sites that don't have enough L1 plots to calculate

# harvest intensity

sites_no_intensity <- c(

30000609, 30000789, 30000799, 30001508, 30001516,

30001570, 30001595, 30001623, 30001635, 30001647,

30001986, 30002042, 30002054, 30002063, 30002129,

30002353, 30002780, 30002791, 30002853, 30003466,

30005821, 30005884, 30005943, 30005964, 30005994,

30006576, 30006595, 30006631, 30006677, 30006747)

# Exclude sites based on the list: Down from 528 to 498 sites

ecolevol5 <- ecolevol4 %>%

filter(!magp_site_id %in% sites_no_intensity)

# 498 sites

nrow(ecolevol4)

nrow(ecolevol5)

unique(ecolevol5$magp_site_id)

#############################################################

### MERGE FILE WITH HARVESTING INTENSITY BACK TO BASEFILE ###

#############################################################

# Reload harvest dataset

# Load revised dataset of harvest intensity

harvest_merge <- fread("C:/Users/martigau/OneDrive - NRCan RNCan/Documents/Personnel/Travail/EcolEvol/NFI Data/QC_PSP/magp_treatment_intensity2.csv")

colnames(harvest_merge)

## Make sure columns are numeric

harvest_merge$treatment_year <- as.numeric(harvest_merge$treatment_year)

harvest_merge$treatment_year2 <- as.numeric(harvest_merge$treatment_year2)

harvest_merge$time_since_trt <- as.numeric(harvest_merge$time_since_trt)

harvest_merge$treatment_intensity <- as.numeric(harvest_merge$treatment_intensity)

harvest_merge$treatment_pct <- as.numeric(harvest_merge$treatment_pct)

unique(harvest_merge$treatment_intensity)

# Repopulate S1 rows

harvest_merge2 <- harvest_merge %>%

bind_rows(harvest_merge %>% mutate(plot_type_id = 'S1'))

nrow(harvest_merge)

nrow(harvest_merge2)

# Remove columns from dataset

# Keep selected variables

harvest_merge3 <- harvest_merge2 %>%

dplyr :: select ("magp_site_id", "plot_type_id", "meas_num", "meas_year",

"treatment_type", "treatment_year", "treatment_year2", "time_since_trt",

"treatment_intensity", "treatment_pct", "notes")

# Merge by 'magp_site_id', 'plot_type_id' and 'meas_year'

ecolevol7 <- ecolevol5 %>%

left_join(harvest_merge3, by = c("magp_site_id", "plot_type_id", "meas_num", "meas_year"))

print(ecolevol7)

colnames(ecolevol7)

# Check result:

ncol(ecolevol5)

ncol(ecolevol7)

# Check result: Same number of rows :0)

nrow(ecolevol5)

nrow(ecolevol7)

## Save the table to a CSV file

file_path <- "C:/Users/martigau/OneDrive - NRCan RNCan/Documents/Personnel/Travail/EcolEvol/NFI Data/ecolevol7.csv"

write.csv(ecolevol7, file = file_path)

write.csv2(ecolevol7, 'ecolevol7_fr.csv', row.names = FALSE)

ecolevol7 <- fread("C:/Users/martigau/OneDrive - NRCan RNCan/Documents/Personnel/Travail/EcolEvol/NFI Data/ecolevol7.csv")

# Count the number of measurements for each plot_type_id per magp_site_id

site_measurements <- ecolevol7 %>%

group_by(magp_site_id, plot_type_id) %>%

summarise(count = n(), .groups = "drop")

print(site_measurements, n = 996)

# Step 1: Count the number of measurements for each plot_type_id, magp_site_id, and meas_year

site_measurements_yearly <- ecolevol7 %>%

group_by(magp_site_id, plot_type_id, meas_year) %>%

summarise(count = n(), .groups = "drop")

# Step 2: Pivot data to compare the counts of each plot_type_id within each magp_site_id and meas_year

site_measurements_pivot <- site_measurements_yearly %>%

pivot_wider(names_from = plot_type_id, values_from = count, values_fill = list(count = 0))

# Step 3: Identify sites where the counts for plot_type_id differ (i.e., there is a mismatch between plot types)

sites_with_mismatched_counts <- site_measurements_pivot %>%

filter(L1 != S1) # Check if L1 and S1 have different counts for the same site and year

# Step 4: Print the result

print(sites_with_mismatched_counts, n = 327)

## Save the table to a CSV file

file_path <- "C:/Users/martigau/OneDrive - NRCan RNCan/Documents/Personnel/Travail/EcolEvol/NFI Data/sites_with_mismatched_counts.csv"

write.csv(sites_with_mismatched_counts, file = file_path)

write.csv2(sites_with_mismatched_counts, 'sites_with_mismatched_counts_fr.csv', row.names = FALSE)

# Manually filter out the extra measurements using conditions from the output

ecolevol8 <- ecolevol7 %>%

filter(

!(magp_site_id == "30000548" & plot_type_id == "L1" & meas_year == 2008),

!(magp_site_id == "30000554" & plot_type_id == "L1" & meas_year == 2022),

!(magp_site_id == "30000555" & plot_type_id == "L1" & meas_year == 2022),

!(magp_site_id == "30000566" & plot_type_id == "L1" & meas_year == 1970),

!(magp_site_id == "30000566" & plot_type_id == "L1" & meas_year == 1978),

!(magp_site_id == "30000569" & plot_type_id == "L1" & meas_year == 1978),

!(magp_site_id == "30000582" & plot_type_id == "L1" & meas_year == 1978),

!(magp_site_id == "30000583" & plot_type_id == "L1" & meas_year == 1978),

!(magp_site_id == "30000592" & plot_type_id == "L1" & meas_year == 1978),

!(magp_site_id == "30000608" & plot_type_id == "L1" & meas_year == 1978),

!(magp_site_id == "30000608" & plot_type_id == "L1" & meas_year == 1985),

!(magp_site_id == "30000608" & plot_type_id == "L1" & meas_year == 1997),

!(magp_site_id == "30000608" & plot_type_id == "L1" & meas_year == 2011),

!(magp_site_id == "30000610" & plot_type_id == "L1" & meas_year == 1970),

!(magp_site_id == "30000622" & plot_type_id == "L1" & meas_year == 1970),

!(magp_site_id == "30000634" & plot_type_id == "L1" & meas_year == 1970),

!(magp_site_id == "30000634" & plot_type_id == "L1" & meas_year == 1978),

!(magp_site_id == "30000634" & plot_type_id == "L1" & meas_year == 2008),

!(magp_site_id == "30000634" & plot_type_id == "L1" & meas_year == 2022),

!(magp_site_id == "30000635" & plot_type_id == "L1" & meas_year == 1978),

!(magp_site_id == "30000635" & plot_type_id == "L1" & meas_year == 2022),

!(magp_site_id == "30000647" & plot_type_id == "L1" & meas_year == 2022),

!(magp_site_id == "30000649" & plot_type_id == "L1" & meas_year == 1970),

!(magp_site_id == "30000656" & plot_type_id == "L1" & meas_year == 1985),

!(magp_site_id == "30000684" & plot_type_id == "L1" & meas_year == 1970),

!(magp_site_id == "30000684" & plot_type_id == "L1" & meas_year == 1978),

!(magp_site_id == "30000684" & plot_type_id == "L1" & meas_year == 1985),

!(magp_site_id == "30000684" & plot_type_id == "L1" & meas_year == 1997),

!(magp_site_id == "30000685" & plot_type_id == "L1" & meas_year == 2008),

!(magp_site_id == "30000713" & plot_type_id == "L1" & meas_year == 1978),

!(magp_site_id == "30000728" & plot_type_id == "L1" & meas_year == 1978),

!(magp_site_id == "30000728" & plot_type_id == "L1" & meas_year == 2016),

!(magp_site_id == "30000736" & plot_type_id == "L1" & meas_year == 1970),

!(magp_site_id == "30000736" & plot_type_id == "L1" & meas_year == 1978),

!(magp_site_id == "30000736" & plot_type_id == "L1" & meas_year == 1985),

!(magp_site_id == "30000736" & plot_type_id == "L1" & meas_year == 1997),

!(magp_site_id == "30000764" & plot_type_id == "L1" & meas_year == 1978),

!(magp_site_id == "30000764" & plot_type_id == "L1" & meas_year == 2009),

!(magp_site_id == "30000783" & plot_type_id == "L1" & meas_year == 1997),

!(magp_site_id == "30000793" & plot_type_id == "L1" & meas_year == 1985),

!(magp_site_id == "30000802" & plot_type_id == "L1" & meas_year == 1985),

!(magp_site_id == "30000824" & plot_type_id == "L1" & meas_year == 2016),

!(magp_site_id == "30000845" & plot_type_id == "L1" & meas_year == 1997),

!(magp_site_id == "30000845" & plot_type_id == "L1" & meas_year == 2012),

!(magp_site_id == "30000878" & plot_type_id == "L1" & meas_year == 2019),

!(magp_site_id == "30000898" & plot_type_id == "L1" & meas_year == 2012),

!(magp_site_id == "30000927" & plot_type_id == "L1" & meas_year == 1980),

!(magp_site_id == "30001001" & plot_type_id == "L1" & meas_year == 1978),

!(magp_site_id == "30001019" & plot_type_id == "L1" & meas_year == 1978),

!(magp_site_id == "30001019" & plot_type_id == "L1" & meas_year == 1985),

!(magp_site_id == "30001210" & plot_type_id == "L1" & meas_year == 1978),

!(magp_site_id == "30001212" & plot_type_id == "L1" & meas_year == 2003),

!(magp_site_id == "30001395" & plot_type_id == "L1" & meas_year == 2015),

!(magp_site_id == "30001417" & plot_type_id == "L1" & meas_year == 1971),

!(magp_site_id == "30001417" & plot_type_id == "L1" & meas_year == 1978),

!(magp_site_id == "30001417" & plot_type_id == "L1" & meas_year == 1989),

!(magp_site_id == "30001435" & plot_type_id == "L1" & meas_year == 1971),

!(magp_site_id == "30001435" & plot_type_id == "L1" & meas_year == 1978),

!(magp_site_id == "30001450" & plot_type_id == "L1" & meas_year == 1989),

!(magp_site_id == "30001451" & plot_type_id == "L1" & meas_year == 1978),

!(magp_site_id == "30001453" & plot_type_id == "L1" & meas_year == 1978),

!(magp_site_id == "30001453" & plot_type_id == "L1" & meas_year == 1989),

!(magp_site_id == "30001453" & plot_type_id == "L1" & meas_year == 2003),

!(magp_site_id == "30001591" & plot_type_id == "L1" & meas_year == 2006),

!(magp_site_id == "30001594" & plot_type_id == "L1" & meas_year == 2006),

!(magp_site_id == "30001600" & plot_type_id == "L1" & meas_year == 1995),

!(magp_site_id == "30001616" & plot_type_id == "L1" & meas_year == 1978),

!(magp_site_id == "30001616" & plot_type_id == "L1" & meas_year == 1989),

!(magp_site_id == "30001616" & plot_type_id == "L1" & meas_year == 1995),

!(magp_site_id == "30001616" & plot_type_id == "L1" & meas_year == 2006),

!(magp_site_id == "30001622" & plot_type_id == "L1" & meas_year == 2006),

!(magp_site_id == "30001639" & plot_type_id == "L1" & meas_year == 2006),

!(magp_site_id == "30001639" & plot_type_id == "L1" & meas_year == 2019),

!(magp_site_id == "30001642" & plot_type_id == "L1" & meas_year == 1989),

!(magp_site_id == "30001644" & plot_type_id == "L1" & meas_year == 1971),

!(magp_site_id == "30001644" & plot_type_id == "L1" & meas_year == 1978),

!(magp_site_id == "30001650" & plot_type_id == "L1" & meas_year == 1971),

!(magp_site_id == "30001650" & plot_type_id == "L1" & meas_year == 1997),

!(magp_site_id == "30001654" & plot_type_id == "L1" & meas_year == 1978),

!(magp_site_id == "30001654" & plot_type_id == "L1" & meas_year == 2021),

!(magp_site_id == "30001952" & plot_type_id == "L1" & meas_year == 1978),

!(magp_site_id == "30001957" & plot_type_id == "L1" & meas_year == 1971),

!(magp_site_id == "30001975" & plot_type_id == "L1" & meas_year == 1971),

!(magp_site_id == "30001975" & plot_type_id == "L1" & meas_year == 1978),

!(magp_site_id == "30001984" & plot_type_id == "L1" & meas_year == 1971),

!(magp_site_id == "30001984" & plot_type_id == "L1" & meas_year == 1978),

!(magp_site_id == "30001995" & plot_type_id == "L1" & meas_year == 1971),

!(magp_site_id == "30001995" & plot_type_id == "L1" & meas_year == 1989),

!(magp_site_id == "30002001" & plot_type_id == "L1" & meas_year == 1989),

!(magp_site_id == "30002001" & plot_type_id == "L1" & meas_year == 1995),

!(magp_site_id == "30002001" & plot_type_id == "L1" & meas_year == 2019),

!(magp_site_id == "30002009" & plot_type_id == "L1" & meas_year == 1978),

!(magp_site_id == "30002011" & plot_type_id == "L1" & meas_year == 1995),

!(magp_site_id == "30002011" & plot_type_id == "L1" & meas_year == 2006),

!(magp_site_id == "30002011" & plot_type_id == "L1" & meas_year == 2019),

!(magp_site_id == "30002014" & plot_type_id == "L1" & meas_year == 1997),

!(magp_site_id == "30002020" & plot_type_id == "L1" & meas_year == 1989),

!(magp_site_id == "30002020" & plot_type_id == "L1" & meas_year == 2006),

!(magp_site_id == "30002024" & plot_type_id == "L1" & meas_year == 1978),

!(magp_site_id == "30002024" & plot_type_id == "L1" & meas_year == 1997),

!(magp_site_id == "30002024" & plot_type_id == "L1" & meas_year == 2006),

!(magp_site_id == "30002034" & plot_type_id == "L1" & meas_year == 1971),

!(magp_site_id == "30002034" & plot_type_id == "L1" & meas_year == 1978),

!(magp_site_id == "30002034" & plot_type_id == "L1" & meas_year == 1989),

!(magp_site_id == "30002040" & plot_type_id == "L1" & meas_year == 1989),

!(magp_site_id == "30002127" & plot_type_id == "L1" & meas_year == 1971),

!(magp_site_id == "30002127" & plot_type_id == "L1" & meas_year == 1978),

!(magp_site_id == "30002141" & plot_type_id == "L1" & meas_year == 1989),

!(magp_site_id == "30002157" & plot_type_id == "L1" & meas_year == 1978),

!(magp_site_id == "30002157" & plot_type_id == "L1" & meas_year == 1989),

!(magp_site_id == "30002157" & plot_type_id == "L1" & meas_year == 2003),

!(magp_site_id == "30002178" & plot_type_id == "L1" & meas_year == 1978),

!(magp_site_id == "30002202" & plot_type_id == "L1" & meas_year == 2003),

!(magp_site_id == "30002218" & plot_type_id == "L1" & meas_year == 1978),

!(magp_site_id == "30002220" & plot_type_id == "L1" & meas_year == 1989),

!(magp_site_id == "30002220" & plot_type_id == "L1" & meas_year == 2006),

!(magp_site_id == "30002260" & plot_type_id == "L1" & meas_year == 1978),

!(magp_site_id == "30002260" & plot_type_id == "L1" & meas_year == 1989),

!(magp_site_id == "30002273" & plot_type_id == "L1" & meas_year == 1971),

!(magp_site_id == "30002273" & plot_type_id == "L1" & meas_year == 1978),

!(magp_site_id == "30002273" & plot_type_id == "L1" & meas_year == 1989),

!(magp_site_id == "30002273" & plot_type_id == "L1" & meas_year == 1997),

!(magp_site_id == "30002287" & plot_type_id == "L1" & meas_year == 1978),

!(magp_site_id == "30002287" & plot_type_id == "L1" & meas_year == 1997),

!(magp_site_id == "30002295" & plot_type_id == "L1" & meas_year == 1971),

!(magp_site_id == "30002295" & plot_type_id == "L1" & meas_year == 1997),

!(magp_site_id == "30002415" & plot_type_id == "L1" & meas_year == 1971),

!(magp_site_id == "30002416" & plot_type_id == "L1" & meas_year == 1978),

!(magp_site_id == "30002425" & plot_type_id == "L1" & meas_year == 1972),

!(magp_site_id == "30002425" & plot_type_id == "L1" & meas_year == 1979),

!(magp_site_id == "30002425" & plot_type_id == "L1" & meas_year == 1997),

!(magp_site_id == "30002757" & plot_type_id == "L1" & meas_year == 1989),

!(magp_site_id == "30002794" & plot_type_id == "L1" & meas_year == 2018),

!(magp_site_id == "30002795" & plot_type_id == "L1" & meas_year == 1979),

!(magp_site_id == "30002832" & plot_type_id == "L1" & meas_year == 2018),

!(magp_site_id == "30002852" & plot_type_id == "L1" & meas_year == 1979),

!(magp_site_id == "30002852" & plot_type_id == "L1" & meas_year == 1989),

!(magp_site_id == "30002852" & plot_type_id == "L1" & meas_year == 2003),

!(magp_site_id == "30002871" & plot_type_id == "L1" & meas_year == 1972),

!(magp_site_id == "30002872" & plot_type_id == "L1" & meas_year == 2003),

!(magp_site_id == "30002905" & plot_type_id == "L1" & meas_year == 1979),

!(magp_site_id == "30002950" & plot_type_id == "L1" & meas_year == 1972),

!(magp_site_id == "30002962" & plot_type_id == "L1" & meas_year == 1972),

!(magp_site_id == "30002963" & plot_type_id == "L1" & meas_year == 1989),

!(magp_site_id == "30003094" & plot_type_id == "L1" & meas_year == 2003),

!(magp_site_id == "30003094" & plot_type_id == "L1" & meas_year == 2016),

!(magp_site_id == "30003174" & plot_type_id == "L1" & meas_year == 1972),

!(magp_site_id == "30003174" & plot_type_id == "L1" & meas_year == 1979),

!(magp_site_id == "30003313" & plot_type_id == "L1" & meas_year == 1972),

!(magp_site_id == "30003313" & plot_type_id == "L1" & meas_year == 1979),

!(magp_site_id == "30003336" & plot_type_id == "L1" & meas_year == 1979),

!(magp_site_id == "30003347" & plot_type_id == "L1" & meas_year == 1972),

!(magp_site_id == "30003553" & plot_type_id == "L1" & meas_year == 1979),

!(magp_site_id == "30003553" & plot_type_id == "L1" & meas_year == 1989),

!(magp_site_id == "30003553" & plot_type_id == "L1" & meas_year == 1997),

!(magp_site_id == "30003583" & plot_type_id == "L1" & meas_year == 1972),

!(magp_site_id == "30003610" & plot_type_id == "L1" & meas_year == 1972),

!(magp_site_id == "30003610" & plot_type_id == "L1" & meas_year == 1979),

!(magp_site_id == "30003610" & plot_type_id == "L1" & meas_year == 2018),

!(magp_site_id == "30004438" & plot_type_id == "L1" & meas_year == 2003),

!(magp_site_id == "30004447" & plot_type_id == "L1" & meas_year == 1975),

!(magp_site_id == "30004447" & plot_type_id == "L1" & meas_year == 1993),

!(magp_site_id == "30004489" & plot_type_id == "L1" & meas_year == 1975),

!(magp_site_id == "30004489" & plot_type_id == "L1" & meas_year == 1981),

!(magp_site_id == "30004489" & plot_type_id == "L1" & meas_year == 2017),

!(magp_site_id == "30004492" & plot_type_id == "L1" & meas_year == 1981),

!(magp_site_id == "30004501" & plot_type_id == "L1" & meas_year == 2020),

!(magp_site_id == "30004613" & plot_type_id == "L1" & meas_year == 1981),

!(magp_site_id == "30005127" & plot_type_id == "L1" & meas_year == 1975),

!(magp_site_id == "30005127" & plot_type_id == "L1" & meas_year == 1983),

!(magp_site_id == "30005127" & plot_type_id == "L1" & meas_year == 2003),

!(magp_site_id == "30005338" & plot_type_id == "L1" & meas_year == 1976),

!(magp_site_id == "30005522" & plot_type_id == "L1" & meas_year == 1976),

!(magp_site_id == "30005522" & plot_type_id == "L1" & meas_year == 1984),

!(magp_site_id == "30005522" & plot_type_id == "L1" & meas_year == 1989),

!(magp_site_id == "30005522" & plot_type_id == "L1" & meas_year == 1997),

!(magp_site_id == "30005523" & plot_type_id == "L1" & meas_year == 1976),

!(magp_site_id == "30005529" & plot_type_id == "L1" & meas_year == 2019),

!(magp_site_id == "30005535" & plot_type_id == "L1" & meas_year == 1987),

!(magp_site_id == "30005545" & plot_type_id == "L1" & meas_year == 1984),

!(magp_site_id == "30005545" & plot_type_id == "L1" & meas_year == 1989),

!(magp_site_id == "30005545" & plot_type_id == "L1" & meas_year == 2008),

!(magp_site_id == "30005546" & plot_type_id == "L1" & meas_year == 1989),

!(magp_site_id == "30005560" & plot_type_id == "L1" & meas_year == 1976),

!(magp_site_id == "30005560" & plot_type_id == "L1" & meas_year == 1984),

!(magp_site_id == "30005570" & plot_type_id == "L1" & meas_year == 1983),

!(magp_site_id == "30005570" & plot_type_id == "L1" & meas_year == 1995),

!(magp_site_id == "30005570" & plot_type_id == "L1" & meas_year == 2006),

!(magp_site_id == "30005596" & plot_type_id == "L1" & meas_year == 1995),

!(magp_site_id == "30005596" & plot_type_id == "L1" & meas_year == 2006),

!(magp_site_id == "30005629" & plot_type_id == "L1" & meas_year == 1977),

!(magp_site_id == "30005633" & plot_type_id == "L1" & meas_year == 1989),

!(magp_site_id == "30005635" & plot_type_id == "L1" & meas_year == 1977),

!(magp_site_id == "30005635" & plot_type_id == "L1" & meas_year == 1984),

!(magp_site_id == "30005635" & plot_type_id == "L1" & meas_year == 1989),

!(magp_site_id == "30005653" & plot_type_id == "L1" & meas_year == 1976),

!(magp_site_id == "30005653" & plot_type_id == "L1" & meas_year == 1984),

!(magp_site_id == "30005655" & plot_type_id == "L1" & meas_year == 1984),

!(magp_site_id == "30005678" & plot_type_id == "L1" & meas_year == 1989),

!(magp_site_id == "30005678" & plot_type_id == "L1" & meas_year == 2015),

!(magp_site_id == "30005730" & plot_type_id == "L1" & meas_year == 1997),

!(magp_site_id == "30005730" & plot_type_id == "L1" & meas_year == 2008),

!(magp_site_id == "30005738" & plot_type_id == "L1" & meas_year == 2008),

!(magp_site_id == "30005756" & plot_type_id == "L1" & meas_year == 1976),

!(magp_site_id == "30005758" & plot_type_id == "L1" & meas_year == 1976),

!(magp_site_id == "30005764" & plot_type_id == "L1" & meas_year == 1989),

!(magp_site_id == "30005825" & plot_type_id == "L1" & meas_year == 1976),

!(magp_site_id == "30005837" & plot_type_id == "L1" & meas_year == 1985),

!(magp_site_id == "30005838" & plot_type_id == "L1" & meas_year == 1976),

!(magp_site_id == "30005847" & plot_type_id == "L1" & meas_year == 1987),

!(magp_site_id == "30005855" & plot_type_id == "L1" & meas_year == 1997),

!(magp_site_id == "30005855" & plot_type_id == "L1" & meas_year == 2008),

!(magp_site_id == "30005876" & plot_type_id == "L1" & meas_year == 1986),

!(magp_site_id == "30005879" & plot_type_id == "L1" & meas_year == 2011),

!(magp_site_id == "30005880" & plot_type_id == "L1" & meas_year == 1976),

!(magp_site_id == "30005883" & plot_type_id == "L1" & meas_year == 1976),

!(magp_site_id == "30005883" & plot_type_id == "L1" & meas_year == 1984),

!(magp_site_id == "30005883" & plot_type_id == "L1" & meas_year == 1989),

!(magp_site_id == "30005894" & plot_type_id == "L1" & meas_year == 1976),

!(magp_site_id == "30005894" & plot_type_id == "L1" & meas_year == 1984),

!(magp_site_id == "30005894" & plot_type_id == "L1" & meas_year == 1989),

!(magp_site_id == "30005898" & plot_type_id == "L1" & meas_year == 1984),

!(magp_site_id == "30005899" & plot_type_id == "L1" & meas_year == 1997),

!(magp_site_id == "30005899" & plot_type_id == "L1" & meas_year == 2008),

!(magp_site_id == "30005903" & plot_type_id == "L1" & meas_year == 1976),

!(magp_site_id == "30005903" & plot_type_id == "L1" & meas_year == 1984),

!(magp_site_id == "30005903" & plot_type_id == "L1" & meas_year == 1997),

!(magp_site_id == "30005903" & plot_type_id == "L1" & meas_year == 2019),

!(magp_site_id == "30005909" & plot_type_id == "L1" & meas_year == 1997),

!(magp_site_id == "30005931" & plot_type_id == "L1" & meas_year == 1976),

!(magp_site_id == "30005931" & plot_type_id == "L1" & meas_year == 1987),

!(magp_site_id == "30005932" & plot_type_id == "L1" & meas_year == 1976),

!(magp_site_id == "30005932" & plot_type_id == "L1" & meas_year == 2019),

!(magp_site_id == "30005983" & plot_type_id == "L1" & meas_year == 2011),

!(magp_site_id == "30005983" & plot_type_id == "L1" & meas_year == 2022),

!(magp_site_id == "30006015" & plot_type_id == "L1" & meas_year == 2022),

!(magp_site_id == "30006024" & plot_type_id == "L1" & meas_year == 2022),

!(magp_site_id == "30006029" & plot_type_id == "L1" & meas_year == 1976),

!(magp_site_id == "30006037" & plot_type_id == "L1" & meas_year == 1988),

!(magp_site_id == "30006038" & plot_type_id == "L1" & meas_year == 1976),

!(magp_site_id == "30006038" & plot_type_id == "L1" & meas_year == 1988),

!(magp_site_id == "30006403" & plot_type_id == "L1" & meas_year == 1987),

!(magp_site_id == "30006403" & plot_type_id == "L1" & meas_year == 1997),

!(magp_site_id == "30006405" & plot_type_id == "L1" & meas_year == 1976),

!(magp_site_id == "30006405" & plot_type_id == "L1" & meas_year == 1987),

!(magp_site_id == "30006405" & plot_type_id == "L1" & meas_year == 2019),

!(magp_site_id == "30006413" & plot_type_id == "L1" & meas_year == 1997),

!(magp_site_id == "30006416" & plot_type_id == "L1" & meas_year == 1976),

!(magp_site_id == "30006416" & plot_type_id == "L1" & meas_year == 2011),

!(magp_site_id == "30006472" & plot_type_id == "L1" & meas_year == 2019),

!(magp_site_id == "30006480" & plot_type_id == "L1" & meas_year == 2019),

!(magp_site_id == "30006572" & plot_type_id == "L1" & meas_year == 1986),

!(magp_site_id == "30006572" & plot_type_id == "L1" & meas_year == 2007),

!(magp_site_id == "30006585" & plot_type_id == "L1" & meas_year == 1984),

!(magp_site_id == "30006596" & plot_type_id == "L1" & meas_year == 1984),

!(magp_site_id == "30006596" & plot_type_id == "L1" & meas_year == 1989),

!(magp_site_id == "30006602" & plot_type_id == "L1" & meas_year == 1983),

!(magp_site_id == "30006602" & plot_type_id == "L1" & meas_year == 1989),

!(magp_site_id == "30006603" & plot_type_id == "L1" & meas_year == 1983),

!(magp_site_id == "30006603" & plot_type_id == "L1" & meas_year == 1989),

!(magp_site_id == "30006603" & plot_type_id == "L1" & meas_year == 1995),

!(magp_site_id == "30006603" & plot_type_id == "L1" & meas_year == 2006),

!(magp_site_id == "30006606" & plot_type_id == "L1" & meas_year == 1984),

!(magp_site_id == "30006606" & plot_type_id == "L1" & meas_year == 1997),

!(magp_site_id == "30006611" & plot_type_id == "L1" & meas_year == 1987),

!(magp_site_id == "30006621" & plot_type_id == "L1" & meas_year == 1984),

!(magp_site_id == "30006621" & plot_type_id == "L1" & meas_year == 1989),

!(magp_site_id == "30006621" & plot_type_id == "L1" & meas_year == 2020),

!(magp_site_id == "30006622" & plot_type_id == "L1" & meas_year == 1976),

!(magp_site_id == "30006623" & plot_type_id == "L1" & meas_year == 1976),

!(magp_site_id == "30006623" & plot_type_id == "L1" & meas_year == 1984),

!(magp_site_id == "30006623" & plot_type_id == "L1" & meas_year == 1989),

!(magp_site_id == "30006623" & plot_type_id == "L1" & meas_year == 1997),

!(magp_site_id == "30006642" & plot_type_id == "L1" & meas_year == 1976),

!(magp_site_id == "30006650" & plot_type_id == "L1" & meas_year == 1976),

!(magp_site_id == "30006650" & plot_type_id == "L1" & meas_year == 1984),

!(magp_site_id == "30006668" & plot_type_id == "L1" & meas_year == 2015),

!(magp_site_id == "30006669" & plot_type_id == "L1" & meas_year == 2003),

!(magp_site_id == "30006669" & plot_type_id == "L1" & meas_year == 2015),

!(magp_site_id == "30006670" & plot_type_id == "L1" & meas_year == 1984),

!(magp_site_id == "30006670" & plot_type_id == "L1" & meas_year == 1989),

!(magp_site_id == "30006670" & plot_type_id == "L1" & meas_year == 2015),

!(magp_site_id == "30006673" & plot_type_id == "L1" & meas_year == 1984),

!(magp_site_id == "30006690" & plot_type_id == "L1" & meas_year == 1976),

!(magp_site_id == "30006695" & plot_type_id == "L1" & meas_year == 2019),

!(magp_site_id == "30006697" & plot_type_id == "L1" & meas_year == 1995),

!(magp_site_id == "30006697" & plot_type_id == "L1" & meas_year == 2006),

!(magp_site_id == "30006697" & plot_type_id == "L1" & meas_year == 2019),

!(magp_site_id == "30006700" & plot_type_id == "L1" & meas_year == 1984),

!(magp_site_id == "30006700" & plot_type_id == "L1" & meas_year == 1989),

!(magp_site_id == "30006700" & plot_type_id == "L1" & meas_year == 1995),

!(magp_site_id == "30006700" & plot_type_id == "L1" & meas_year == 2006),

!(magp_site_id == "30006701" & plot_type_id == "L1" & meas_year == 1984),

!(magp_site_id == "30006701" & plot_type_id == "L1" & meas_year == 1989),

!(magp_site_id == "30006719" & plot_type_id == "L1" & meas_year == 1989),

!(magp_site_id == "30006719" & plot_type_id == "L1" & meas_year == 2006),

!(magp_site_id == "30006720" & plot_type_id == "L1" & meas_year == 1983),

!(magp_site_id == "30006722" & plot_type_id == "L1" & meas_year == 1976),

!(magp_site_id == "30006722" & plot_type_id == "L1" & meas_year == 1984),

!(magp_site_id == "30006722" & plot_type_id == "L1" & meas_year == 1989),

!(magp_site_id == "30006736" & plot_type_id == "L1" & meas_year == 1976),

!(magp_site_id == "30006736" & plot_type_id == "L1" & meas_year == 2006),

!(magp_site_id == "30006736" & plot_type_id == "L1" & meas_year == 2019),

!(magp_site_id == "30006737" & plot_type_id == "L1" & meas_year == 1976),

!(magp_site_id == "30006737" & plot_type_id == "L1" & meas_year == 1984),

!(magp_site_id == "30006737" & plot_type_id == "L1" & meas_year == 1989),

!(magp_site_id == "30006737" & plot_type_id == "L1" & meas_year == 1995),

!(magp_site_id == "30006748" & plot_type_id == "L1" & meas_year == 2019),

!(magp_site_id == "30006749" & plot_type_id == "L1" & meas_year == 1976),

!(magp_site_id == "30006755" & plot_type_id == "L1" & meas_year == 1986),

!(magp_site_id == "30006817" & plot_type_id == "L1" & meas_year == 2021),

!(magp_site_id == "30006859" & plot_type_id == "L1" & meas_year == 1976),

!(magp_site_id == "30006859" & plot_type_id == "L1" & meas_year == 1986),

!(magp_site_id == "30007083" & plot_type_id == "L1" & meas_year == 1986),

!(magp_site_id == "30007094" & plot_type_id == "L1" & meas_year == 1976),

!(magp_site_id == "30007094" & plot_type_id == "L1" & meas_year == 1986),

!(magp_site_id == "30006539" & plot_type_id == "L1" & meas_year == 1984),

!(magp_site_id == "30006539" & plot_type_id == "L1" & meas_year == 1989),

!(magp_site_id == "30006704" & plot_type_id == "L1" & meas_year == 1989),

!(magp_site_id == "30002010" & plot_type_id == "S1" & meas_year == 2006),

!(magp_site_id == "30004469" & plot_type_id == "S1" & meas_year == 1992),

!(magp_site_id == "30004492" & plot_type_id == "S1" & meas_year == 1992),

#!(magp_site_id == "30308718" & plot_type_id == "L1" & meas_year == 2016 & total_density_sum == 1175),

!(magp_site_id == "30308723" & plot_type_id == "L1" & meas_year == 2017 & total_density_sum == 50),

!(magp_site_id == "30308723" & plot_type_id == "L1" & meas_year == 2022 & total_density_sum == 650),

!(magp_site_id == "30454625" & plot_type_id == "S1" & meas_num == 2),

!(magp_site_id == "30454625" & plot_type_id == "L1" & meas_num == 2)

)

# Check result: 4421 down to 4086 rows

nrow(ecolevol7)

nrow(ecolevol8)

# Filter out treated sites without harvest intensity

ecolevol9 <- ecolevol8 %>%

filter(treatment_intensity != -1 | is.na(treatment_intensity))

# Check result: 4086 down to 3228 rows

nrow(ecolevol8)

nrow(ecolevol9)

# Save data as .csv

file_path <- "C:/Users/martigau/OneDrive - NRCan RNCan/Documents/Personnel/Travail/EcolEvol/NFI Data/ecolevol9.csv"

write.csv(ecolevol9, file = file_path)

write.csv2(ecolevol9, 'ecolevol9_fr.csv', row.names = FALSE)

# Remove duplicate measurements: same site, plot_id, meas_year

# Load revised dataset

ecolevol9 <- fread("C:/Users/martigau/OneDrive - NRCan RNCan/Documents/Personnel/Travail/EcolEvol/NFI Data/ecolevol9.csv")

colnames(ecolevol9)

# Remove the "V1" column if it exists

# Remove the column "V1" using data.table syntax

ecolevol9[, V1 := NULL]

# Remove all columns named "V1"

ecolevol9[, (colnames(ecolevol9)[grepl("V1", colnames(ecolevol9))]) := NULL]

# Count rows by plot_type_id before pivot

ecolevol9 %>%

group_by(magp_site_id, plot_type_id, meas_num, meas_year) %>%

summarise(count = n()) %>%

print(n = Inf) # This will print all matching rows without limit

print(summarise)

duplicate_rows <- ecolevol9 %>%

group_by(magp_site_id, plot_type_id, meas_num, meas_year) %>%

filter(n() > 1) %>%

ungroup()

# View problematic magp_site_id and meas_year combinations

duplicate_rows %>%

select(magp_site_id, plot_type_id, meas_num, meas_year) %>%

distinct()

print(duplicate_rows, n = 216)

# Save data as .csv

file_path <- "C:/Users/martigau/OneDrive - NRCan RNCan/Documents/Personnel/Travail/EcolEvol/NFI Data/duplicate_rows.csv"

write.csv(duplicate_rows, file = file_path)

write.csv2(duplicate_rows, 'duplicate_rows_fr.csv', row.names = FALSE)

# Remove duplicates

ecolevol10 <- ecolevol9 %>%

arrange(magp_site_id, plot_type_id, meas_num, meas_year) %>% # Optional: to ensure sorting if needed

distinct(magp_site_id, plot_type_id, meas_num, meas_year, .keep_all = TRUE)

# Check: Down from 3228 to 3068 rows

nrow(ecolevol9)

nrow(ecolevol10)

print(ecolevol10)

# Save data as .csv

file_path <- "C:/Users/martigau/OneDrive - NRCan RNCan/Documents/Personnel/Travail/EcolEvol/NFI Data/ecolevol10.csv"

write.csv(ecolevol10, file = file_path)

write.csv2(ecolevol10, 'ecolevol10_fr.csv', row.names = FALSE)

# Validate if harvesting was attributed to all potential sites

treatment_type_y_na <- ecolevol10 %>%

filter(treatment_type.x %in% c("CL", "CLE", "CLR") & is.na(treatment_type.y))

print(treatment_type_y_na)

unique(treatment_type_y_na$magp_site_id)

# Save data as .csv

file_path <- "C:/Users/martigau/OneDrive - NRCan RNCan/Documents/Personnel/Travail/EcolEvol/NFI Data/treatment_type_y_na.csv"

write.csv(treatment_type_y_na, file = file_path)

write.csv2(treatment_type_y_na, 'treatment_type_y_na_fr.csv', row.names = FALSE)

# Exclude some sites because I can't assign an intensity or measure

# a treatment response (no measurement after treatment):

ecolevol11 <- ecolevol10 %>%

filter (

!(magp_site_id %in% c("30001975", "30002001", "30002157",

"30002202", "30002220", "30002260",

"30002261", "30002295", "30006472", "30005127"))

)

# Confirm: Down from 3068 to 2996

nrow(ecolevol10)

nrow(ecolevol11)

unique(ecolevol11$magp_site_id)

# Assign treatment intensity info to additional 21 sites

ecolevol12 <- ecolevol11 %>%

mutate(

#30001644

treatment_type.y = ifelse(

magp_site_id == "30001644" & is.na(treatment_type.y),

"CLR",

treatment_type.y

),

treatment_year.y = ifelse(

magp_site_id == "30001644" & is.na(treatment_year.y),

2006,

treatment_year.y

),

treatment_year2 = ifelse(

magp_site_id == "30001644" & is.na(treatment_year2),

2006,

treatment_year2

),

time_since_trt = ifelse(

magp_site_id == "30001644" & is.na(time_since_trt),

meas_year - treatment_year2,

time_since_trt

),

treatment_intensity = ifelse(

magp_site_id == "30001644" & is.na(treatment_intensity),

0.626,

treatment_intensity

),

treatment_pct.y = ifelse(

magp_site_id == "30001644" & is.na(treatment_pct.y),

0.626,

treatment_pct.y

),

# 30001952

treatment_type.y = ifelse(

magp_site_id == "30001952" & is.na(treatment_type.y),

"CL",

treatment_type.y

),

treatment_year.y = ifelse(

magp_site_id == "30001952" & is.na(treatment_year.y),

1999,

treatment_year.y

),

treatment_year2 = ifelse(

magp_site_id == "30001952" & is.na(treatment_year2),

1999,

treatment_year2

),

time_since_trt = ifelse(

magp_site_id == "30001952" & is.na(time_since_trt),

meas_year - treatment_year2,

time_since_trt

),

treatment_intensity = ifelse(

magp_site_id == "30001952" & is.na(treatment_intensity),

0.848,

treatment_intensity

),

treatment_pct.y = ifelse(

magp_site_id == "30001952" & is.na(treatment_pct.y),

0.848,

treatment_pct.y

),

# 30001998

treatment_type.y = ifelse(

magp_site_id == "30001998" & is.na(treatment_type.y),

"CL",

treatment_type.y

),

treatment_year.y = ifelse(

magp_site_id == "30001998" & is.na(treatment_year.y),

2002,

treatment_year.y

),

treatment_year2 = ifelse(

magp_site_id == "30001998" & is.na(treatment_year2),

2002,

treatment_year2

),

time_since_trt = ifelse(

magp_site_id == "30001998" & is.na(time_since_trt),

meas_year - treatment_year2,

time_since_trt

),

treatment_intensity = ifelse(

magp_site_id == "30001998" & is.na(treatment_intensity),

0.700,

treatment_intensity

),

treatment_pct.y = ifelse(

magp_site_id == "30001998" & is.na(treatment_pct.y),

0.700,

treatment_pct.y

),

# 30002047

treatment_type.y = ifelse(

magp_site_id == "30002047" & is.na(treatment_type.y),

"CL",

treatment_type.y

),

treatment_year.y = ifelse(

magp_site_id == "30002047" & is.na(treatment_year.y),

1991,

treatment_year.y

),

treatment_year2 = ifelse(

magp_site_id == "30002047" & is.na(treatment_year2),

1991,

treatment_year2

),

time_since_trt = ifelse(

magp_site_id == "30002047" & is.na(time_since_trt),

meas_year - treatment_year2,

time_since_trt

),

treatment_intensity = ifelse(

magp_site_id == "30002047" & is.na(treatment_intensity),

0.126,

treatment_intensity

),

treatment_pct.y = ifelse(

magp_site_id == "30002047" & is.na(treatment_pct.y),

0.126,

treatment_pct.y

),

# 30002051

treatment_type.y = ifelse(

magp_site_id == "30002051" & is.na(treatment_type.y),

"CL",

treatment_type.y

),

treatment_year.y = ifelse(

magp_site_id == "30002051" & is.na(treatment_year.y),

2006,

treatment_year.y

),

treatment_year2 = ifelse(

magp_site_id == "30002051" & is.na(treatment_year2),

2006,

treatment_year2

),

time_since_trt = ifelse(

magp_site_id == "30002051" & is.na(time_since_trt),

meas_year - treatment_year2,

time_since_trt

),

treatment_intensity = ifelse(

magp_site_id == "30002051" & is.na(treatment_intensity),

0.672,

treatment_intensity

),

treatment_pct.y = ifelse(

magp_site_id == "30002051" & is.na(treatment_pct.y),

0.627,

treatment_pct.y

),

# 30002160

treatment_type.y = ifelse(

magp_site_id == "30002160" & is.na(treatment_type.y),

"CL",

treatment_type.y

),

treatment_year.y = ifelse(

magp_site_id == "30002160" & is.na(treatment_year.y),

1995,

treatment_year.y

),

treatment_year2 = ifelse(

magp_site_id == "30002160" & is.na(treatment_year2),

1995,

treatment_year2

),

time_since_trt = ifelse(

magp_site_id == "30002160" & is.na(time_since_trt),

meas_year - treatment_year2,

time_since_trt

),

treatment_intensity = ifelse(

magp_site_id == "30002160" & is.na(treatment_intensity),

0.592,

treatment_intensity

),

treatment_pct.y = ifelse(

magp_site_id == "30002160" & is.na(treatment_pct.y),

0.592,

treatment_pct.y

),

# 30002162

treatment_type.y = ifelse(

magp_site_id == "30002162" & is.na(treatment_type.y),

"CLR",

treatment_type.y

),

treatment_year.y = ifelse(

magp_site_id == "30002162" & is.na(treatment_year.y),

2003,

treatment_year.y

),

treatment_year2 = ifelse(

magp_site_id == "30002162" & is.na(treatment_year2),

2003,

treatment_year2

),

time_since_trt = ifelse(

magp_site_id == "30002162" & is.na(time_since_trt),

meas_year - treatment_year2,

time_since_trt

),

treatment_intensity = ifelse(

magp_site_id == "30002162" & is.na(treatment_intensity),

0.492,

treatment_intensity

),

treatment_pct.y = ifelse(

magp_site_id == "30002162" & is.na(treatment_pct.y),

0.492,

treatment_pct.y

),

# 30002178

treatment_type.y = ifelse(

magp_site_id == "30002178" & is.na(treatment_type.y),

"CL",

treatment_type.y

),

treatment_year.y = ifelse(

magp_site_id == "30002178" & is.na(treatment_year.y),

1997,

treatment_year.y

),

treatment_year2 = ifelse(

magp_site_id == "30002178" & is.na(treatment_year2),

1997,

treatment_year2

),

time_since_trt = ifelse(

magp_site_id == "30002178" & is.na(time_since_trt),

meas_year - treatment_year2,

time_since_trt

),

treatment_intensity = ifelse(

magp_site_id == "30002178" & is.na(treatment_intensity),

0.550,

treatment_intensity

),

treatment_pct.y = ifelse(

magp_site_id == "30002178" & is.na(treatment_pct.y),

0.550,

treatment_pct.y

),

# 30002219

treatment_type.y = ifelse(

magp_site_id == "30002219" & is.na(treatment_type.y),

"CLR",

treatment_type.y

),

treatment_year.y = ifelse(

magp_site_id == "30002219" & is.na(treatment_year.y),

1989,

treatment_year.y

),

treatment_year2 = ifelse(

magp_site_id == "30002219" & is.na(treatment_year2),

1989,

treatment_year2

),

time_since_trt = ifelse(

magp_site_id == "30002219" & is.na(time_since_trt),

meas_year - treatment_year2,

time_since_trt

),

treatment_intensity = ifelse(

magp_site_id == "30002219" & is.na(treatment_intensity),

0.646,

treatment_intensity

),

treatment_pct.y = ifelse(

magp_site_id == "30002219" & is.na(treatment_pct.y),

0.646,

treatment_pct.y

),

# 30002219

treatment_type.y = ifelse(

magp_site_id == "30002219" & is.na(treatment_type.y),

"CLR",

treatment_type.y

),

treatment_year.y = ifelse(

magp_site_id == "30002219" & is.na(treatment_year.y),

1989,

treatment_year.y

),

treatment_year2 = ifelse(

magp_site_id == "30002219" & is.na(treatment_year2),

1989,

treatment_year2

),

time_since_trt = ifelse(

magp_site_id == "30002219" & is.na(time_since_trt),

meas_year - treatment_year2,

time_since_trt

),

treatment_intensity = ifelse(

magp_site_id == "30002219" & is.na(treatment_intensity),

0.646,

treatment_intensity

),

treatment_pct.y = ifelse(

magp_site_id == "30002219" & is.na(treatment_pct.y),

0.646,

treatment_pct.y

),

# 30002414

treatment_type.y = ifelse(

magp_site_id == "30002414" & is.na(treatment_type.y),

"CL",

treatment_type.y

),

treatment_year.y = ifelse(

magp_site_id == "30002414" & is.na(treatment_year.y),

2000,

treatment_year.y

),

treatment_year2 = ifelse(

magp_site_id == "30002414" & is.na(treatment_year2),

2000,

treatment_year2

),

time_since_trt = ifelse(

magp_site_id == "30002414" & is.na(time_since_trt),

meas_year - treatment_year2,

time_since_trt

),

treatment_intensity = ifelse(

magp_site_id == "30002414" & is.na(treatment_intensity),

0.697,

treatment_intensity

),

treatment_pct.y = ifelse(

magp_site_id == "30002414" & is.na(treatment_pct.y),

0.697,

treatment_pct.y

),

# 30002416

treatment_type.y = ifelse(

magp_site_id == "30002416" & is.na(treatment_type.y),

"CL",

treatment_type.y

),

treatment_year.y = ifelse(

magp_site_id == "30002416" & is.na(treatment_year.y),

2000,

treatment_year.y

),

treatment_year2 = ifelse(

magp_site_id == "30002416" & is.na(treatment_year2),

2000,

treatment_year2

),

time_since_trt = ifelse(

magp_site_id == "30002416" & is.na(time_since_trt),

meas_year - treatment_year2,

time_since_trt

),

treatment_intensity = ifelse(

magp_site_id == "30002416" & is.na(treatment_intensity),

0.609,

treatment_intensity

),

treatment_pct.y = ifelse(

magp_site_id == "30002416" & is.na(treatment_pct.y),

0.609,

treatment_pct.y

),

# 30002823

treatment_type.y = ifelse(

magp_site_id == "30002823" & is.na(treatment_type.y),

"CL",

treatment_type.y

),

treatment_year.y = ifelse(

magp_site_id == "30002823" & is.na(treatment_year.y),

1998,

treatment_year.y

),

treatment_year2 = ifelse(

magp_site_id == "30002823" & is.na(treatment_year2),

1998,

treatment_year2

),

time_since_trt = ifelse(

magp_site_id == "30002823" & is.na(time_since_trt),

meas_year - treatment_year2,

time_since_trt

),

treatment_intensity = ifelse(

magp_site_id == "30002823" & is.na(treatment_intensity),

0.700,

treatment_intensity

),

treatment_pct.y = ifelse(

magp_site_id == "30002823" & is.na(treatment_pct.y),

0.700,

treatment_pct.y

),

# 30002920

treatment_type.y = ifelse(

magp_site_id == "30002920" & is.na(treatment_type.y),

"CL",

treatment_type.y

),

treatment_year.y = ifelse(

magp_site_id == "30002920" & is.na(treatment_year.y),

1994,

treatment_year.y

),

treatment_year2 = ifelse(

magp_site_id == "30002920" & is.na(treatment_year2),

1994,

treatment_year2

),

time_since_trt = ifelse(

magp_site_id == "30002920" & is.na(time_since_trt),

meas_year - treatment_year2,

time_since_trt

),

treatment_intensity = ifelse(

magp_site_id == "30002920" & is.na(treatment_intensity),

0.404,

treatment_intensity

),

treatment_pct.y = ifelse(

magp_site_id == "30002920" & is.na(treatment_pct.y),

0.404,

treatment_pct.y

),

# 30002933

treatment_type.y = ifelse(

magp_site_id == "30002933" & is.na(treatment_type.y),

"CL",

treatment_type.y

),

treatment_year.y = ifelse(

magp_site_id == "30002933" & is.na(treatment_year.y),

1995,

treatment_year.y

),

treatment_year2 = ifelse(

magp_site_id == "30002933" & is.na(treatment_year2),

1995,

treatment_year2

),

time_since_trt = ifelse(

magp_site_id == "30002933" & is.na(time_since_trt),

meas_year - treatment_year2,

time_since_trt

),

treatment_intensity = ifelse(

magp_site_id == "30002933" & is.na(treatment_intensity),

0.518,

treatment_intensity

),

treatment_pct.y = ifelse(

magp_site_id == "30002933" & is.na(treatment_pct.y),

0.518,

treatment_pct.y

),

# 30002936

treatment_type.y = ifelse(

magp_site_id == "30002936" & is.na(treatment_type.y),

"CL",

treatment_type.y

),

treatment_year.y = ifelse(

magp_site_id == "30002936" & is.na(treatment_year.y),

1997,

treatment_year.y

),

treatment_year2 = ifelse(

magp_site_id == "30002936" & is.na(treatment_year2),

1997,

treatment_year2

),

time_since_trt = ifelse(

magp_site_id == "30002936" & is.na(time_since_trt),

meas_year - treatment_year2,

time_since_trt

),

treatment_intensity = ifelse(

magp_site_id == "30002936" & is.na(treatment_intensity),

0.693,

treatment_intensity

),

treatment_pct.y = ifelse(

magp_site_id == "30002936" & is.na(treatment_pct.y),

0.693,

treatment_pct.y

),

# 30003583

treatment_type.y = ifelse(

magp_site_id == "30003583" & is.na(treatment_type.y),

"CL",

treatment_type.y

),

treatment_year.y = ifelse(

magp_site_id == "30003583" & is.na(treatment_year.y),

2000,

treatment_year.y

),

treatment_year2 = ifelse(

magp_site_id == "30003583" & is.na(treatment_year2),

2000,

treatment_year2

),

time_since_trt = ifelse(

magp_site_id == "30003583" & is.na(time_since_trt),

meas_year - treatment_year2,

time_since_trt

),

treatment_intensity = ifelse(

magp_site_id == "30003583" & is.na(treatment_intensity),

0.483,

treatment_intensity

),

treatment_pct.y = ifelse(

magp_site_id == "30003583" & is.na(treatment_pct.y),

0.483,

treatment_pct.y

),

# 30006032

treatment_type.y = ifelse(

magp_site_id == "30006032" & is.na(treatment_type.y),

"CL",

treatment_type.y

),

treatment_year.y = ifelse(

magp_site_id == "30006032" & is.na(treatment_year.y),

1996,

treatment_year.y

),

treatment_year2 = ifelse(

magp_site_id == "30006032" & is.na(treatment_year2),

1996,

treatment_year2

),

time_since_trt = ifelse(

magp_site_id == "30006032" & is.na(time_since_trt),

meas_year - treatment_year2,

time_since_trt

),

treatment_intensity = ifelse(

magp_site_id == "30006032" & is.na(treatment_intensity),

0.529,

treatment_intensity

),

treatment_pct.y = ifelse(

magp_site_id == "30006032" & is.na(treatment_pct.y),

0.529,

treatment_pct.y

),

# 30006539

treatment_type.y = ifelse(

magp_site_id == "30006539" & is.na(treatment_type.y),

"CL",

treatment_type.y

),

treatment_year.y = ifelse(

magp_site_id == "30006539" & is.na(treatment_year.y),

2004,

treatment_year.y

),

treatment_year2 = ifelse(

magp_site_id == "30006539" & is.na(treatment_year2),

2004,

treatment_year2

),

time_since_trt = ifelse(

magp_site_id == "30006539" & is.na(time_since_trt),

meas_year - treatment_year2,

time_since_trt

),

treatment_intensity = ifelse(

magp_site_id == "30006539" & is.na(treatment_intensity),

0.797,

treatment_intensity

),

treatment_pct.y = ifelse(

magp_site_id == "30006539" & is.na(treatment_pct.y),

0.797,

treatment_pct.y

),

# 30006622

treatment_type.y = ifelse(

magp_site_id == "30006622" & is.na(treatment_type.y),

"CL",

treatment_type.y

),

treatment_year.y = ifelse(

magp_site_id == "30006622" & is.na(treatment_year.y),

2004,

treatment_year.y

),

treatment_year2 = ifelse(

magp_site_id == "30006622" & is.na(treatment_year2),

2004,

treatment_year2

),

time_since_trt = ifelse(

magp_site_id == "30006622" & is.na(time_since_trt),

meas_year - treatment_year2,

time_since_trt

),

treatment_intensity = ifelse(

magp_site_id == "30006622" & is.na(treatment_intensity),

0.732,

treatment_intensity

),

treatment_pct.y = ifelse(

magp_site_id == "30006622" & is.na(treatment_pct.y),

0.732,

treatment_pct.y

),

# 30006724

treatment_type.y = ifelse(

magp_site_id == "30006724" & is.na(treatment_type.y),

"CLR",

treatment_type.y

),

treatment_year.y = ifelse(

magp_site_id == "30006724" & is.na(treatment_year.y),

1988,

treatment_year.y

),

treatment_year2 = ifelse(

magp_site_id == "30006724" & is.na(treatment_year2),

1988,

treatment_year2

),

time_since_trt = ifelse(

magp_site_id == "30006724" & is.na(time_since_trt),

meas_year - treatment_year2,

time_since_trt

),

treatment_intensity = ifelse(

magp_site_id == "30006724" & is.na(treatment_intensity),

0.389,

treatment_intensity

),

treatment_pct.y = ifelse(

magp_site_id == "30006724" & is.na(treatment_pct.y),

0.389,

treatment_pct.y

),

)

# Save data as .csv

file_path <- "C:/Users/martigau/OneDrive - NRCan RNCan/Documents/Personnel/Travail/EcolEvol/NFI Data/ecolevol12.csv"

write.csv(ecolevol12, file = file_path)

write.csv2(ecolevol12, 'ecolevol12_fr.csv', row.names = FALSE)

# Load revised dataset

ecolevol12 <- fread("C:/Users/martigau/OneDrive - NRCan RNCan/Documents/Personnel/Travail/EcolEvol/NFI Data/ecolevol12.csv")

colnames(ecolevol12)

# Add a numerical ecozone variable

ecolevol13 <- ecolevol12 %>%

mutate(

ecozone2 = case_when(

ecozone == 'Atlantic Highlands' ~ 1,

ecozone == 'Mixedwood Plains' ~ 2,

ecozone == 'Boreal Shield' ~ 3,

TRUE ~ NA_real_ # Handle any other cases where ecozone is not recognized

)

)

unique(ecolevol13$ecozone)

# Assign 'CON' to treatment_type.y

ecolevol14 <- ecolevol13 %>%

mutate(

treatment_type.y = case_when(

treatment_type.x == "CON" ~ "CON", # Only change to "CON" if treatment_type.x is "CON"

TRUE ~ treatment_type.y # Keep existing values otherwise

),

treatment_intensity = case_when(

treatment_type.x == "CON" ~ 0, # Set intensity to 0 if treatment_type.x is "CON"

TRUE ~ treatment_intensity # Keep existing values otherwise

),

treatment_pct.y = case_when(

treatment_type.x == "CON" ~ 0, # Set pct to 0 if treatment_type.x is "CON"

TRUE ~ treatment_pct.y # Keep existing values otherwise

)

)

nrow(ecolevol13)

nrow(ecolevol14)

# 396 study sites

unique(ecolevol14$magp_site_id)

# Add categorical harvest intensity

ecolevol15 <- ecolevol14 %>%

mutate(

category = case_when(

treatment_pct.y == 0 ~ "Control" ,

treatment_pct.y >= 0.05 & treatment_pct.y <= 0.4099 ~ "Low",

treatment_pct.y >= 0.410 & treatment_pct.y <= 0.8 ~ "High",

treatment_pct.y > 0.8 ~ "Very high",

TRUE ~ "Other"

)

)

unique(ecolevol15$category)

# Add general cut vs. uncut column

ecolevol16 <- ecolevol15 %>%

mutate(

silv = case_when(

treatment_type.y == "CON" ~ "Uncut",

TRUE ~ "Cut"

)

)

unique(ecolevol16$silv)

# Save data as .csv

file_path <- "C:/Users/martigau/OneDrive - NRCan RNCan/Documents/Personnel/Travail/EcolEvol/NFI Data/ecolevol16.csv"

write.csv(ecolevol16, file = file_path)

write.csv2(ecolevol16, 'ecolevol16_fr.csv', row.names = FALSE)

#########################################################

#########################################################

#########################################################

# Load revised dataset

ecolevol16 <- fread("C:/Users/martigau/OneDrive - NRCan RNCan/Documents/Personnel/Travail/EcolEvol/NFI Data/ecolevol16.csv")

colnames(ecolevol16)

# Remove columns by index that have the name "V1"

v1_columns <- which(colnames(ecolevol16) == "V1")

ecolevol16[, (v1_columns) := NULL]

# Verify the columns are removed

colnames(ecolevol16)

# Select specific columns using data.table syntax

ecolevol17 <- ecolevol16[, .(

magp_site_id,

plot_type_id,

meas_num,

meas_year,

province,

ecozone,

ecozone2,

longitude,

latitude,

elevation,

aspect_class,

slope_position,

slope_class,

sources,

category,

silv,

treatment_type.x,

treatment_type.y,

treatment_year.x,

treatment_year.y,

treatment_year2,

time_since_trt,

treatment_intensity,

treatment_pct.x,

treatment_pct.y,

notes,

bbd_count,

pct_dens_bbd,

total_density_sum,

density_ACER.SAH,

density_FAGU.GRA,

pct_density_ACER.SAH,

pct_density_FAGU.GRA,

total_basal_area_sum,

ba_ACER.SAH,

ba_FAGU.GRA,

pct_basal_area_ACER.SAH,

pct_basal_area_FAGU.GRA

)]

# Verify the column names to ensure it's correct

colnames(ecolevol17)

# If bbd_count is zero, pct_dens_bbd is zero as well

ecolevol18 <- ecolevol17 %>%

mutate(pct_dens_bbd = ifelse(bbd_count == 0, 0, pct_dens_bbd))

unique(ecolevol18$treatment_type.y)

# Save data as .csv

file_path <- "C:/Users/martigau/OneDrive - NRCan RNCan/Documents/Personnel/Travail/EcolEvol/NFI Data/ecolevol18.csv"

write.csv(ecolevol18, file = file_path)

write.csv2(ecolevol18, 'ecolevol18_fr.csv', row.names = FALSE)

ecolevol18 %>%

group_by(magp_site_id, plot_type_id, meas_year) %>%

summarise(count = n(), .groups = "drop") %>%

#print(n = Inf) # Print all rows without truncating

filter(count > 1)

# Delete duplicate rows in site 30454625

ecolevol20 <- ecolevol18 %>%

filter(

!(magp_site_id == "30454625" & meas_num == "2")

)

# Count the number of measurements for each plot_type_id per magp_site_id

site_measurements <- ecolevol20 %>%

group_by(magp_site_id, plot_type_id) %>%

summarise(count = n(), .groups = "drop")

print(site_measurements, n = 850)

# Count rows by plot_type_id before pivot

ecolevol20 %>%

group_by(plot_type_id) %>%

summarise(count = n())

problem_rows2 <- ecolevol20 %>%

filter(plot_type_id == "S1") %>%

group_by(magp_site_id, meas_year) %>%

filter(n() > 1) %>%

ungroup()

# View problematic magp_site_id and meas_year combinations

# Use dplyr functions to get distinct combinations

problem_rows2 %>%

dplyr::select(magp_site_id, meas_year) %>%

distinct()

ecolevol20 %>%

group_by(magp_site_id, plot_type_id, meas_num, meas_year) %>%

summarise(count = n(), .groups = "drop") %>%

filter(count > 1) # Show duplicates before pivoting

ecolevol20 %>%

group_by(magp_site_id, meas_num, meas_year, plot_type_id) %>%

summarise(num_rows = n(), .groups = "drop") %>%

filter(num_rows > 1) # Show where multiple rows exist for the same combination

ecolevol20 %>%

group_by(magp_site_id, meas_num, meas_year, plot_type_id) %>%

summarise(

num_rows = n(),

distinct_columns = n_distinct(

province,

ecozone,

ecozone2,

longitude,

latitude,

elevation,

aspect_class,

slope_position,

slope_class,

sources,

category,

silv), # Add any columns you think might differ

.groups = "drop"

) %>%

filter(num_rows > 1)

sites_with_na_before_pivot <- ecolevol20 %>%

filter(is.na(pct_basal_area_ACER.SAH)) %>% # Filter rows where pct_basal_area_ACER.SAH is NA

dplyr::select(magp_site_id, meas_num, meas_year) # Select the site ID column (or other columns you want)

# To view the list of sites

print(sites_with_na_before_pivot)

# Save data as .csv

file_path <- "C:/Users/martigau/OneDrive - NRCan RNCan/Documents/Personnel/Travail/EcolEvol/NFI Data/ecolevol20.csv"

write.csv(ecolevol20, file = file_path)

write.csv2(ecolevol20, 'ecolevol20_fr.csv', row.names = FALSE)

#Pivot wider time

## Make sure columns are numeric

str(ecolevol20)

ecolevol_wide <- ecolevol20 %>%

pivot_wider(

id_cols = c(

magp_site_id, meas_num, meas_year, province, ecozone, ecozone2, longitude, latitude,

elevation, aspect_class, slope_position, slope_class, sources, category, silv,

treatment_type.x, treatment_type.y, treatment_year.x, treatment_year.y,

treatment_year2, time_since_trt, treatment_intensity, treatment_pct.x,

treatment_pct.y, notes, bbd_count, pct_dens_bbd

), # Group by these columns

names_from = plot_type_id, # Create new columns based on 'plot_type_id' values ('L1', 'S1')

values_from = c(

total_density_sum,

density_ACER.SAH,

density_FAGU.GRA,

pct_density_ACER.SAH,

pct_density_FAGU.GRA,

total_basal_area_sum,

ba_ACER.SAH,

ba_FAGU.GRA,

pct_basal_area_ACER.SAH,

pct_basal_area_FAGU.GRA

),

names_glue = "{plot_type_id}_{.value}" # Create new column names combining plot_type_id and the variable name

)

#1536

nrow(ecolevol_wide)

sites_with_na <- ecolevol_wide %>%

filter(is.na(S1_pct_basal_area_ACER.SAH)) %>% # Filter rows where S1_pct_basal_area_ACER.SAH is NA

dplyr::select(magp_site_id, meas_num, meas_year)

# To view the list of sites

print(sites_with_na, n = 38)

sites_with_na_FAGU.GRA <- ecolevol_wide %>%

filter(is.na(S1_pct_basal_area_FAGU.GRA)) %>%

dplyr::select(magp_site_id, meas_num, meas_year)

# To view the list of sites: same plots and years as ACER.SAH

print(sites_with_na_FAGU.GRA, n = 38)

unique(ecolevol20$magp_site_id)

# Manually reshape the data for all specified variables

ecolevol_wide <- ecolevol20 %>%

group_by(magp_site_id, meas_num, meas_year, province, ecozone, ecozone2, longitude, latitude,

elevation, aspect_class, slope_position, slope_class, sources, category, silv,

treatment_type.x, treatment_type.y, treatment_year.x, treatment_year.y, treatment_year2,

time_since_trt, treatment_intensity, treatment_pct.x, treatment_pct.y, notes, bbd_count, pct_dens_bbd) %>%

summarise(

L1_total_density_sum = sum(total_density_sum[plot_type_id == "L1"], na.rm = TRUE),

S1_total_density_sum = sum(total_density_sum[plot_type_id == "S1"], na.rm = TRUE),

L1_density_ACER.SAH = sum(density_ACER.SAH[plot_type_id == "L1"], na.rm = TRUE),

S1_density_ACER.SAH = sum(density_ACER.SAH[plot_type_id == "S1"], na.rm = TRUE),

L1_density_FAGU.GRA = sum(density_FAGU.GRA[plot_type_id == "L1"], na.rm = TRUE),

S1_density_FAGU.GRA = sum(density_FAGU.GRA[plot_type_id == "S1"], na.rm = TRUE),

L1_pct_density_ACER.SAH = sum(pct_density_ACER.SAH[plot_type_id == "L1"], na.rm = TRUE),

S1_pct_density_ACER.SAH = sum(pct_density_ACER.SAH[plot_type_id == "S1"], na.rm = TRUE),

L1_pct_density_FAGU.GRA = sum(pct_density_FAGU.GRA[plot_type_id == "L1"], na.rm = TRUE),

S1_pct_density_FAGU.GRA = sum(pct_density_FAGU.GRA[plot_type_id == "S1"], na.rm = TRUE),

L1_total_basal_area_sum = sum(total_basal_area_sum[plot_type_id == "L1"], na.rm = TRUE),

S1_total_basal_area_sum = sum(total_basal_area_sum[plot_type_id == "S1"], na.rm = TRUE),

L1_ba_ACER.SAH = sum(ba_ACER.SAH[plot_type_id == "L1"], na.rm = TRUE),

S1_ba_ACER.SAH = sum(ba_ACER.SAH[plot_type_id == "S1"], na.rm = TRUE),

L1_ba_FAGU.GRA = sum(ba_FAGU.GRA[plot_type_id == "L1"], na.rm = TRUE),

S1_ba_FAGU.GRA = sum(ba_FAGU.GRA[plot_type_id == "S1"], na.rm = TRUE),

L1_pct_basal_area_ACER.SAH = sum(pct_basal_area_ACER.SAH[plot_type_id == "L1"], na.rm = TRUE),

S1_pct_basal_area_ACER.SAH = sum(pct_basal_area_ACER.SAH[plot_type_id == "S1"], na.rm = TRUE),

L1_pct_basal_area_FAGU.GRA = sum(pct_basal_area_FAGU.GRA[plot_type_id == "L1"], na.rm = TRUE),

S1_pct_basal_area_FAGU.GRA = sum(pct_basal_area_FAGU.GRA[plot_type_id == "S1"], na.rm = TRUE),

.groups = "drop"

)

# Check the reshaped data

head(ecolevol_wide)

#1536

nrow(ecolevol_wide)

sites_with_na_man <- ecolevol_wide %>%

filter(is.na(S1_pct_basal_area_ACER.SAH)) %>% # Filter rows where S1_pct_basal_area_ACER.SAH is NA

dplyr::select(magp_site_id, meas_num, meas_year)

print(sites_with_na_man)

duplicates_after_pivot <- ecolevol_wide %>%

group_by(magp_site_id, meas_num, meas_year) %>%

summarise(count = n(), .groups = "drop") %>%

filter(count > 1)

print(duplicates_after_pivot, n = 38)

# Save data as .csv

file_path <- "C:/Users/martigau/OneDrive - NRCan RNCan/Documents/Personnel/Travail/EcolEvol/NFI Data/duplicates_after_pivot.csv"

write.csv(duplicates_after_pivot, file = file_path)

write.csv2(duplicates_after_pivot, 'duplicates_after_pivot_fr.csv', row.names = FALSE)

## Save the table to a CSV file

file_path <- "C:/Users/martigau/OneDrive - NRCan RNCan/Documents/Personnel/Travail/EcolEvol/NFI Data/ecolevol_wide.csv"

write.csv(ecolevol_wide, file = file_path)

write.csv2(ecolevol_wide, 'ecolevol_wide_fr.csv', row.names = FALSE)

colnames(ecolevol_wide)

#########################################################

#########################################################

#########################################################

# Load revised dataset

ecolevol_wide_corrected <- fread("C:/Users/martigau/OneDrive - NRCan RNCan/Documents/Personnel/Travail/EcolEvol/NFI Data/ecolevol_wide_corrected.csv")

# Exclude clearcuts

ecolevol_wide2 <- ecolevol_wide_corrected %>%

filter(!magp_site_id %in% c("30000667", "30000673", "30000740",

"30001543", "30001591", "30001630")

)

ecolevol_wide2b <- ecolevol_wide2 %>%

mutate(pct_dens_bbd = coalesce(pct_dens_bbd, 0))

ecolevol_wide2c <- ecolevol_wide2b %>%

mutate(bbd_count = coalesce(bbd_count, 0))

# Total of 390 sites

colnames(ecolevol_wide2c)

nrow(ecolevol_wide2c)

nrow(ecolevol_wide2c)

unique(ecolevol_wide2c$magp_site_id)

## Save the table to a CSV file

file_path <- "C:/Users/martigau/OneDrive - NRCan RNCan/Documents/Personnel/Travail/EcolEvol/NFI Data/ecolevol_wide2c.csv"

write.csv(ecolevol_wide2c, file = file_path)

write.csv2(ecolevol_wide2c, 'ecolevol_wide2c_fr.csv', row.names = FALSE)

colnames(ecolevol_wide2c)

#ecolevol_wide2c <- ecolevol_wide2c %>%

#dplyr::select(-V1)

# Add preharvest variables

ecolevol_wide3 <- ecolevol_wide2c %>%

arrange(magp_site_id, meas_num) %>%

group_by(magp_site_id) %>%

mutate(

initial_L1_pct_ba_ACER.SAH = first(L1_pct_basal_area_ACER.SAH),

initial_L1_pct_ba_FAGU.GRA = first(L1_pct_basal_area_FAGU.GRA),

initial_S1_pct_ba_ACER.SAH = first(S1_pct_basal_area_ACER.SAH),

initial_S1_ba_ACER.SAH = first(S1_ba_ACER.SAH),

initial_S1_pct_ba_FAGU.GRA = first(S1_pct_basal_area_FAGU.GRA)

) %>%

ungroup()

# Applying value change manually - initial not first measurement

ecolevol_wide3b <- ecolevol_wide3 %>%

mutate(initial_L1_pct_ba_ACER.SAH = case_when(

magp_site_id == 30000736 & initial_L1_pct_ba_ACER.SAH ~ 65,

magp_site_id == 30455102 & initial_L1_pct_ba_ACER.SAH ~ 59,

TRUE ~ initial_L1_pct_ba_ACER.SAH # Keep original value if no condition is met

))

# Plot #30000736 in Quebec was a sugar maple stand at meas_num = 0, but

# measurements 0-3 had to be removed to match measurements of saplings (S1)

# and trees (L1).

# Plot #30455102 in ON, value should be 59%

unique(ecolevol_wide6$magp_site_id)

# View the result

print(ecolevol_wide3b)

str(ecolevol_wide3b)

# Define values between 0 and 1

ecolevol_wide4 <- ecolevol_wide3b %>%

mutate(

pct_dens_bbd = pct_dens_bbd / 100,

S1_pct_basal_area_ACER.SAH = S1_pct_basal_area_ACER.SAH / 100,

S1_pct_basal_area_FAGU.GRA = S1_pct_basal_area_FAGU.GRA / 100,

L1_pct_basal_area_ACER.SAH = L1_pct_basal_area_ACER.SAH / 100,

L1_pct_basal_area_FAGU.GRA = L1_pct_basal_area_FAGU.GRA / 100,

initial_L1_pct_ba_ACER.SAH = initial_L1_pct_ba_ACER.SAH / 100,

initial_L1_pct_ba_FAGU.GRA = initial_L1_pct_ba_FAGU.GRA / 100,

initial_S1_pct_ba_ACER.SAH = initial_S1_pct_ba_ACER.SAH / 100,

initial_S1_pct_ba_FAGU.GRA = initial_S1_pct_ba_FAGU.GRA / 100

)

colnames(ecolevol_wide4)

str(ecolevol_wide4)

## Save the table to a CSV file

file_path <- "C:/Users/martigau/OneDrive - NRCan RNCan/Documents/Personnel/Travail/EcolEvol/NFI Data/ecolevol_wide4.csv"

write.csv(ecolevol_wide4, file = file_path)

write.csv2(ecolevol_wide4, 'ecolevol_wide4_fr.csv', row.names = FALSE)

# Get distinct magp_site_id with their associated longitude, latitude, elevation, and treatment

unique_sites <- ecolevol_wide4 %>%

distinct(magp_site_id, longitude, latitude, elevation, silv, .keep_all = TRUE)

## Save the table to a CSV file

file_path <- "C:/Users/martigau/OneDrive - NRCan RNCan/Documents/Personnel/Travail/EcolEvol/Images/Mapping/MAGPlots.csv"

write.csv(unique_sites, file = file_path)

#########################################################

#########################################################

#### STATISTICAL ANALYSES #####

#########################################################

#########################################################

# Load revised dataset

ecolevol_wide4 <- fread("C:/Users/martigau/OneDrive - NRCan RNCan/Documents/Personnel/Travail/EcolEvol/NFI Data/ecolevol_wide4.csv")

install.packages("tidyverse")

install.packages("lme4")

install.packages("lmerTest")

install.packages("MuMIn")

install.packages("glmmTMB")

install.packages("nlme")

install.packages("car")

install.packages("statmod")

library(statmod)

library(tidyverse)

library(lme4)

library(lmerTest)

library(MuMIn)

library(glmmTMB)

library(nlme)

library(car)

#Load climate dataset

climate_data <- fread("C:/Users/martigau/OneDrive - NRCan RNCan/Documents/Personnel/Travail/EcolEvol/NFI Data/climate_data.csv")

stats_spei <- fread("C:/Users/martigau/OneDrive - NRCan RNCan/Documents/Personnel/Travail/EcolEvol/NFI Data/stats_spei.csv")

colnames(ecolevol_wide4)

ecolevol_wide5 <- ecolevol_wide4 %>%

dplyr::select(-V1)

colnames(ecolevol_wide5)

# Perform the merge using a left join

ecolevol_wide6 <- stats_spei %>%

left_join(ecolevol_wide4, by = "magp_site_id")

colnames(ecolevol_wide6)

ecolevol_wide7 <- climate_data %>%

left_join(ecolevol_wide6, by = "magp_site_id")

colnames(ecolevol_wide7)

# Divide precipitation by 1000 and snow by 100 to get m

ecolevol_wide7b <- ecolevol_wide7 %>%

mutate(

precip_m = total_precip_40_years / 1000,

summer_precip_m = summer_precip_40_years / 1000,

snow_m = total_snow_40_years / 100)

colnames(ecolevol_wide7b)

str(ecolevol_wide6)

ecolevol_wide7c <- ecolevol_wide7b %>%

dplyr::select(-V1.x)

ecolevol_wide7d <- ecolevol_wide7c %>%

dplyr::select(-V1.y)

ecolevol_wide6 <- ecolevol_wide7d %>%

dplyr::select(-V1)

colnames(ecolevol_wide6)

## Save the table to a CSV file

file_path <- "C:/Users/martigau/OneDrive - NRCan RNCan/Documents/Personnel/Travail/EcolEvol/NFI Data/ecolevol_wide6.csv"

write.csv(ecolevol_wide6, file = file_path)

write.csv2(ecolevol_wide6, 'ecolevol_wide6_fr.csv', row.names = FALSE)

##############################################################

### STATISTICAL ANALYSES OF SUGAR MAPLE SAPLING ABUNDANCE ####

##############################################################

# Load dataset

ecolevol_wide6 <- fread("C:/Users/martigau/OneDrive - NRCan RNCan/Documents/Personnel/Travail/EcolEvol/NFI Data/ecolevol_wide6.csv")

colnames(ecolevol_wide6)

ecolevol_wide6 <- ecolevol_wide6 %>%

dplyr::select(-V1)

colnames(ecolevol_wide6)

unique(ecolevol_wide6$pct_dens_bbd)

# Flag the first observation for each magp_site_id

# Sort the data by magp_site_id and meas_year to ensure temporal order

ecolevol_wide6_filtered <- ecolevol_wide6 %>%

arrange(magp_site_id, meas_year) %>% # Sort by magp_site_id and meas_year

group_by(magp_site_id) %>%

mutate(is_first = row_number() == 1) %>% # Flag the first row for each magp_site_id

filter(!is_first) %>% # Remove the first observation

ungroup() # Ungroup after filtering

str(ecolevol_wide6_filtered)

## Save the table to a CSV file

file_path <- "C:/Users/martigau/OneDrive - NRCan RNCan/Documents/Personnel/Travail/EcolEvol/NFI Data/ecolevol_wide6_filtered.csv"

write.csv(ecolevol_wide6_filtered, file = file_path)

write.csv2(ecolevol_wide6_filtered, 'ecolevol_wide6_filtered_fr.csv', row.names = FALSE)

########################################################

#### RELATIVE ABUNDANCE (% BASAL AREA) ##########

########################################################

# Load dataset

ecolevol_wide6_filtered <- fread("C:/Users/martigau/OneDrive - NRCan RNCan/Documents/Personnel/Travail/EcolEvol/NFI Data/ecolevol_wide6_filtered.csv")

colnames(ecolevol_wide6_filtered)

# Compute the correlation matrix for continuous variables

cor_matrix_full <- cor(ecolevol_wide6_filtered[, c("meas_year",

"initial_S1_pct_ba_ACER.SAH",

"L1_pct_basal_area_ACER.SAH",

"S1_pct_basal_area_FAGU.GRA",

"treatment_pct.y",

"mean_temp_40_years",

"mean_temp_40_years_min",

"mean_temp_40_years_max",

"summer_temp_40_years",

"summer_precip_m",

"precip_m",

"snow_m",

"pct_dens_bbd"

)], use = "complete.obs")

print(cor_matrix_full)

## Save the table to a CSV file

file_path <- "C:/Users/martigau/OneDrive - NRCan RNCan/Documents/Personnel/Travail/EcolEvol/NFI Data/cor_matrix_full.csv"

write.csv(cor_matrix_full, file = file_path)

write.csv2(cor_matrix_full, 'cor_matrix_full_fr.csv', row.names = FALSE)

# Compute the correlation matrix for continuous variables

cor_matrix <- cor(ecolevol_wide6_filtered[, c("meas_year",

"initial_S1_pct_ba_ACER.SAH",

"L1_pct_basal_area_ACER.SAH",

"S1_pct_basal_area_FAGU.GRA",

"treatment_pct.y",

"summer_temp_40_years",

"summer_precip_m",

"pct_dens_bbd"

)], use = "complete.obs")

print(cor_matrix)

## Save the table to a CSV file

file_path <- "C:/Users/martigau/OneDrive - NRCan RNCan/Documents/Personnel/Travail/EcolEvol/NFI Data/cor_matrix.csv"

write.csv(cor_matrix, file = file_path)

write.csv2(cor_matrix, 'cor_matrix_fr.csv', row.names = FALSE)

#No issues with collinearity, not one above 0.3 or below -0.3

#Make sure ecozone is used as factor

unique(ecolevol_wide6_filtered$ecozone)

unique(ecolevol_wide6_filtered$category)

levels(ecolevol_wide6_filtered$ecozone)

levels(ecolevol_wide6_filtered$ecozone2)

ecolevol_wide6_filtered$ecozone3 <- factor(ecolevol_wide6_filtered$ecozone2, labels = c("Atlantic Highlands", "Mixedwood Plains", "Boreal Shield"))

levels(ecolevol_wide6_filtered$ecozone3)

# List of candidate models

# 1) FULL MODEL

# 2) STAND CONDITIONS MODEL

# 3) HARVEST INTENSITY MODEL

# 4) REGIONAL CONDITIONS MODEL

# 5) CLIMATE MODEL

# 6) STAND AND HARVEST MODEL

# 7) STAND AND REGION MODEL

# 8) STAND AND CLIMATE MODEL

# 9) HARVEST AND REGION MODEL

# 10) HARVEST AND CLIMATE MODEL

# 11) REGION AND CLIMATE MODEL

# AIC approach

# Fit model1, the global model / full model

model1_full <- lmer(S1_pct_basal_area_ACER.SAH ~

meas_year + L1_pct_basal_area_ACER.SAH +

S1_pct_basal_area_FAGU.GRA +

treatment_pct.y + category +

ecozone3 + pct_dens_bbd +

summer_precip_m + summer_temp_40_years +

initial_S1_pct_ba_ACER.SAH +

(1 | magp_site_id), data = ecolevol_wide6_filtered)

# Fit model2, stand conditions

model2_stand <- lmer(S1_pct_basal_area_ACER.SAH ~

meas_year + L1_pct_basal_area_ACER.SAH +

S1_pct_basal_area_FAGU.GRA +

pct_dens_bbd +

initial_S1_pct_ba_ACER.SAH +

(1 | magp_site_id), data = ecolevol_wide6_filtered)

# Fit model3, the harvesting intensity model

model3_harvest <- lmer(S1_pct_basal_area_ACER.SAH ~

meas_year + treatment_pct.y + category +

initial_S1_pct_ba_ACER.SAH +

(1 | magp_site_id), data = ecolevol_wide6_filtered)

# Fit model4, regional conditions

model4_region <- lmer(S1_pct_basal_area_ACER.SAH ~

meas_year +

ecozone3 +

initial_S1_pct_ba_ACER.SAH +

(1 | magp_site_id), data = ecolevol_wide6_filtered)

# Fit model5, the climate model

model5_climate <- lmer(S1_pct_basal_area_ACER.SAH ~

meas_year +

summer_precip_m + summer_temp_40_years +

initial_S1_pct_ba_ACER.SAH +

(1 | magp_site_id), data = ecolevol_wide6_filtered)

# Fit model6, stand conditions and harvest model

model6_stand_harvest <- lmer(S1_pct_basal_area_ACER.SAH ~

meas_year + L1_pct_basal_area_ACER.SAH +

S1_pct_basal_area_FAGU.GRA + treatment_pct.y +

category + pct_dens_bbd +

initial_S1_pct_ba_ACER.SAH +

(1 | magp_site_id), data = ecolevol_wide6_filtered)

# Fit model7, stand conditions and regions model

model7_stand_region <- lmer(S1_pct_basal_area_ACER.SAH ~

meas_year + L1_pct_basal_area_ACER.SAH +

S1_pct_basal_area_FAGU.GRA + ecozone3 +

pct_dens_bbd +

initial_S1_pct_ba_ACER.SAH +

(1 | magp_site_id), data = ecolevol_wide6_filtered)

# Fit model8, stand conditions and climate model

model8_stand_climate <- lmer(S1_pct_basal_area_ACER.SAH ~

meas_year + L1_pct_basal_area_ACER.SAH +

S1_pct_basal_area_FAGU.GRA +

pct_dens_bbd +

summer_precip_m + summer_temp_40_years +

initial_S1_pct_ba_ACER.SAH +

(1 | magp_site_id), data = ecolevol_wide6_filtered)

# Fit model9, the harvesting intensity and region model

model9_harvest_region <- lmer(S1_pct_basal_area_ACER.SAH ~

meas_year + treatment_pct.y + category +

ecozone3 +

initial_S1_pct_ba_ACER.SAH +

(1 | magp_site_id), data = ecolevol_wide6_filtered)

# Fit model10, the harvesting intensity and climate model

model10_harvest_climate <- lmer(S1_pct_basal_area_ACER.SAH ~

meas_year + treatment_pct.y + category +

summer_precip_m + summer_temp_40_years +

initial_S1_pct_ba_ACER.SAH +

(1 | magp_site_id), data = ecolevol_wide6_filtered)

# Fit model11, regional conditions and climate

model11_region_climate <- lmer(S1_pct_basal_area_ACER.SAH ~

meas_year +

ecozone3 +

summer_precip_m + summer_temp_40_years +

initial_S1_pct_ba_ACER.SAH +

(1 | magp_site_id), data = ecolevol_wide6_filtered)

# Calculate AIC for each model

aic_model1_full <- AIC(model1_full)

aic_model2_stand <- AIC(model2_stand)

aic_model3_harvest <- AIC(model3_harvest)

aic_model4_region <- AIC(model4_region)

aic_model5_climate <- AIC(model5_climate)

aic_model6_stand_harvest <- AIC(model6_stand_harvest)

aic_model7_stand_region <- AIC(model7_stand_region)

aic_model8_stand_climate <- AIC(model8_stand_climate)

aic_model9_harvest_region <- AIC(model9_harvest_region)

aic_model10_harvest_climate <- AIC(model10_harvest_climate)

aic_model11_region_climate <- AIC(model11_region_climate)

# Create a data frame with model names and AIC values

aic_values <- data.frame(

Model = c("Full", "Stand conditions", "Harvest", "Region", "Climate",

"Stand and Harvest", "Stand and Region",

"Stand and Climate", "Harvest and Region",

"Harvest and Climate", "Region and Climate"),

AIC = c(aic_model1_full, aic_model2_stand,

aic_model3_harvest, aic_model4_region, aic_model5_climate,

aic_model6_stand_harvest, aic_model7_stand_region,

aic_model8_stand_climate, aic_model9_harvest_region,

aic_model10_harvest_climate, aic_model11_region_climate)

)

# Print AIC values

print(aic_values)

# Calculate Akaike weights

aic_min <- min(aic_values$AIC) # Find the minimum AIC

# Compute the Akaike weights

aic_values$Delta_AIC <- aic_values$AIC - aic_min # AIC difference from the best model

aic_values$Akaike_Weight <- exp(-0.5 * aic_values$Delta_AIC) / sum(exp(-0.5 * aic_values$Delta_AIC))

# Print results

print(aic_values)

## Save the table to a CSV file

file_path <- "C:/Users/martigau/OneDrive - NRCan RNCan/Documents/Personnel/Travail/EcolEvol/NFI Data/aic_values.csv"

write.csv(aic_values, file = file_path)

write.csv2(aic_values, 'aic_values_fr.csv', row.names = FALSE)

# Print summaries of each model

summary(model1_full)

summary(model2_stand)

summary(model3_harvest)

summary(model4_region)

summary(model5_climate)

summary(model6_stand_harvest)

summary(model7_stand_region)

summary(model8_stand_climate)

summary(model9_harvest_region)

summary(model10_harvest_climate)

summary(model11_region_climate)

# Plot the residuals of models

plot(resid(model1_full))

plot(resid(model2_stand))

plot(resid(model8_stand_climate))

qqnorm(resid(model1_full))

qqline(resid(model1_full), col = "red")

qqnorm(resid(model2_stand))

qqline(resid(model2_stand), col = "red")

qqnorm(resid(model3_harvest))

qqline(resid(model3_harvest), col = "red")

qqnorm(resid(model4_region))

qqline(resid(model4_region), col = "red")

qqnorm(resid(model5_climate))

qqline(resid(model5_climate), col = "red")

qqnorm(resid(model6_stand_harvest))

qqline(resid(model6_stand_harvest), col = "red")

qqnorm(resid(model7_stand_region))

qqline(resid(model7_stand_region), col = "red")

qqnorm(resid(model8_stand_climate))

qqline(resid(model8_stand_climate), col = "red")

qqnorm(resid(model9_harvest_region))

qqline(resid(model9_harvest_region), col = "red")

qqnorm(resid(model10_harvest_climate))

qqline(resid(model10_harvest_climate), col = "red")

qqnorm(resid(model11_region_climate))

qqline(resid(model11_region_climate), col = "red")

# Histogram of residuals to check for normality

hist(resid(model1_full))

hist(resid(model2_stand))

hist(resid(model3_harvest))

hist(resid(model4_region))

hist(resid(model5_climate))

hist(resid(model6_stand_harvest))

hist(resid(model7_stand_region))

hist(resid(model8_stand_climate))

hist(resid(model9_harvest_region))

hist(resid(model10_harvest_climate))

hist(resid(model11_region_climate))

# Multimodel inference

# List of fitted models, e.g., full_model, stand_conditions, etc.

model_list <- list(model1_full, model2_stand, model3_harvest,

model4_region, model5_climate, model6_stand_harvest,

model7_stand_region, model8_stand_climate,

model9_harvest_region, model10_harvest_climate,

model11_region_climate)

# Select the best models based on AIC and delta_AIC

model_selection <- model.sel(model_list)

# Multimodel inference, model-averaged parameter estimates

model_averaged <- model.avg(model_selection)

summary(model_averaged)

confint(model_averaged)

# Calculate marginal and conditional R² for models

r2_values1 <- r.squaredGLMM(model1_full)

r2_values2 <- r.squaredGLMM(model2_stand)

r2_values3 <- r.squaredGLMM(model3_harvest)

r2_values4 <- r.squaredGLMM(model4_region)

r2_values5 <- r.squaredGLMM(model5_climate)

r2_values6 <- r.squaredGLMM(model6_stand_harvest)

r2_values7 <- r.squaredGLMM(model7_stand_region)

r2_values8 <- r.squaredGLMM(model8_stand_climate)

r2_values9 <- r.squaredGLMM(model9_harvest_region)

r2_values10 <- r.squaredGLMM(model10_harvest_climate)

r2_values11 <- r.squaredGLMM(model11_region_climate)

# Print the results

print(r2_values1)

print(r2_values2)

print(r2_values3)

print(r2_values4)

print(r2_values5)

print(r2_values6)

print(r2_values7)

print(r2_values8)

print(r2_values9)

print(r2_values10)

print(r2_values11)

colnames(ecolevol_wide6_filtered)

#####################################################

### CORRELATION MATRIX BETWEEN DEPENDENT VARIABLE ###

### AND ABSOLUTE VALUES OF SAPLING ABUNDANCE ###

#####################################################

print(ecolevol_wide6_filtered)

# Compute the correlation matrix between the dependent variable and

# absolute values of sapling abundance (stems/ha and m2/ha)

cor_matrix_dep <- cor(ecolevol_wide6_filtered[, c(

"S1_pct_basal_area_ACER.SAH",

"S1_pct_basal_area_FAGU.GRA",

"S1_ba_ACER.SAH",

"S1_ba_FAGU.GRA",

"S1_density_ACER.SAH",

"S1_density_FAGU.GRA",

"S1_total_density_sum",

"S1_total_basal_area_sum"

)], use = "complete.obs")

print(cor_matrix_dep)

# Define the columns to compute correlations for

cols <- c(

"S1_pct_basal_area_ACER.SAH",

"S1_pct_basal_area_FAGU.GRA",

"S1_ba_ACER.SAH",

"S1_ba_FAGU.GRA",

"S1_density_ACER.SAH",

"S1_density_FAGU.GRA",

"S1_total_density_sum",

"S1_total_basal_area_sum"

)

# Initialize an empty matrix to store p-values

p_value_matrix <- matrix(NA, nrow = length(cols), ncol = length(cols))

# Compute the correlation matrix and p-value matrix

for(i in 1:length(cols)) {

for(j in 1:length(cols)) {

# Use cor.test to compute the correlation and p-value

test <- cor.test(ecolevol_wide6_filtered[[cols[i]]], ecolevol_wide6_filtered[[cols[j]]], use = "complete.obs")

# Store the p-value in the p_value_matrix

p_value_matrix[i, j] <- test$p.value

}

}

# Convert the p_value_matrix to a more readable form, applying the <.001 condition

p_value_matrix <- apply(p_value_matrix, c(1, 2), function(x) {

if (!is.na(x)) {

if (x < 0.001) {

return("<.001")

} else {

return(format(round(x, 3), nsmall = 3))

}

} else {

return(NA)

}

})

# Compute the correlation matrix

cor_matrix_dep <- cor(ecolevol_wide6_filtered[, cols], use = "complete.obs")

# Print the correlation matrix

print("Correlation Matrix:")

print(cor_matrix_dep)

# Print the p-value matrix

print("P-value Matrix:")

print(p_value_matrix)

## Save the table to a CSV file

file_path <- "C:/Users/martigau/OneDrive - NRCan RNCan/Documents/Personnel/Travail/EcolEvol/NFI Data/cor_matrix_dep.csv"

write.csv(cor_matrix_dep, file = file_path)

write.csv2(cor_matrix_dep, 'cor_matrix_dep_fr.csv', row.names = FALSE)

## Save the table to a CSV file

file_path <- "C:/Users/martigau/OneDrive - NRCan RNCan/Documents/Personnel/Travail/EcolEvol/NFI Data/p_value_matrix.csv"

write.csv(p_value_matrix, file = file_path)

write.csv2(p_value_matrix, 'p_value_matrix_fr.csv', row.names = FALSE)

#Sample size is 1077 observations

# Load the ggplot2 library

library(ggplot2)

######################################################

#### PLOT COUNT OF INITIAL AND FINAL MEASUREMENTS ####

#### SUGAR MAPLE BY ABUNDANCE CATEGORIES ####

#### FIGURE 3 IN MANUSCRIPT ####

######################################################

# Load necessary libraries

library(dplyr)

library(ggplot2)

# Load dataset

ecolevol_wide6_filtered <- fread("C:/Users/martigau/OneDrive - NRCan RNCan/Documents/Personnel/Travail/EcolEvol/NFI Data/ecolevol_wide6_filtered.csv")

ecolevol_wide6 <- fread("C:/Users/martigau/OneDrive - NRCan RNCan/Documents/Personnel/Travail/EcolEvol/NFI Data/ecolevol_wide6.csv")

# Step 1: Categorize 'S1_pct_basal_area_ACER.SAH' values into the defined intervals

ecolevol_wide6_tm <- ecolevol_wide6 %>%

mutate(

initial_value_group = case_when(

initial_S1_pct_ba_ACER.SAH >= 0 & initial_S1_pct_ba_ACER.SAH < 0.25 ~ "0-0.249",

initial_S1_pct_ba_ACER.SAH >= 0.25 & initial_S1_pct_ba_ACER.SAH < 0.5 ~ "0.25-0.499",

initial_S1_pct_ba_ACER.SAH >= 0.5 & initial_S1_pct_ba_ACER.SAH < 0.75 ~ "0.50-0.749",

initial_S1_pct_ba_ACER.SAH >= 0.75 & initial_S1_pct_ba_ACER.SAH <= 1.0 ~ "0.75-1.0",

TRUE ~ "Other" # Categorize everything else as 'Other'

)

)

# Step 2: Create a new column 'final_value_group' based on the last meas_num for each plot

ecolevol_wide6_tma <- ecolevol_wide6_tm %>%

group_by(magp_site_id) %>%

filter(meas_num == max(meas_num)) %>% # Keep the last measurement for each plot

mutate(

final_value_group = case_when(

S1_pct_basal_area_ACER.SAH >= 0 & S1_pct_basal_area_ACER.SAH < 0.25 ~ "0-0.249",

S1_pct_basal_area_ACER.SAH >= 0.25 & S1_pct_basal_area_ACER.SAH < 0.5 ~ "0.25-0.499",

S1_pct_basal_area_ACER.SAH >= 0.5 & S1_pct_basal_area_ACER.SAH < 0.75 ~ "0.50-0.749",

S1_pct_basal_area_ACER.SAH >= 0.75 & S1_pct_basal_area_ACER.SAH <= 1.0 ~ "0.75-1.0",

TRUE ~ "Other" # Categorize everything else as 'Other'

)

) %>%

ungroup() %>%

dplyr::select(magp_site_id, initial_value_group, final_value_group) %>%

distinct(magp_site_id, .keep_all = TRUE) # Keep one row per plot (magp_site_id)

# Step 3: Combine initial and final value groups into a single dataset

plot_data <- ecolevol_wide6_tma %>%

gather(key = "measurement", value = "value_group",

initial_value_group, final_value_group) %>%

filter(value_group != "Other") # Remove "Other" category

# Step 4: Count the number of plots in each value group

plot_counts <- plot_data %>%

group_by(measurement, value_group) %>%

summarise(plot_count = n_distinct(magp_site_id), .groups = "drop")

# Step 5: Reverse the order of the 'measurement' factor levels

plot_counts$measurement <- factor(plot_counts$measurement, levels = c("initial_value_group", "final_value_group"))

# Step 6: Plot the bar graph showing initial and final value groups for each category, side by side

ggplot(plot_counts, aes(x = value_group, y = plot_count, fill = measurement)) +

geom_bar(stat = "identity", position = "dodge2", color = "black") + # Changed "dodge" to "dodge2"

labs(

x = "Relative abundance category (% BA)",

y = "Number of plots"

) +

scale_fill_manual(

values = c("initial_value_group" = "#F5F5F5", "final_value_group" = "#DC143C"),

labels = c("Initial", "Final")

) +

guides(

fill = guide_legend(title = "Sugar maple") # Remove the legend title but keep the labels

) +

scale_x_discrete(

labels = c(

"0-0.249" = "<25",

"0.25-0.499" = "25-49.9",

"0.50-0.749" = "50-74.9",

"0.75-1.0" = ">=75"

)

) +

theme_minimal() +

theme(

panel.border = element_rect(color = "black", fill = NA, linewidth = 0.5), # Black border around the plot panel

panel.grid = element_blank(), # Remove the grid lines

axis.ticks.y = element_line(color = "black", linewidth = 0.5), # Thicker y-axis ticks

axis.ticks.x = element_line(color = "black", linewidth = 0.5) # Thicker x-axis ticks

#axis.text.x = element_text(angle = 45, hjust = 1) # Rotate x-axis labels for better readability

) +

theme(

axis.ticks.length = unit(0.2, "cm") # Adjust length of the tick marks.

)

################################

#### FIGURE 5 ##################

################################

# Final - initial for sugar maple and beech in the same plot

# Load necessary libraries

library(dplyr)

# Load dataset

ecolevol_wide6_filtered <- fread("C:/Users/martigau/OneDrive - NRCan RNCan/Documents/Personnel/Travail/EcolEvol/NFI Data/ecolevol_wide6_filtered.csv")

ecolevol_wide6 <- fread("C:/Users/martigau/OneDrive - NRCan RNCan/Documents/Personnel/Travail/EcolEvol/NFI Data/ecolevol_wide6.csv")

# Step: Calculate the differences for maple and beech

last_measurement <- ecolevol_wide6 %>%

group_by(magp_site_id) %>%

filter(meas_num == max(meas_num)) %>% # Keep the last measurement for each site

ungroup() %>%

mutate(

# Calculate delta_maple: difference between final and initial values for maple

delta_maple = S1_pct_basal_area_ACER.SAH - initial_S1_pct_ba_ACER.SAH,

# Calculate delta_beech: difference between final and initial values for beech

delta_beech = S1_pct_basal_area_FAGU.GRA - initial_S1_pct_ba_FAGU.GRA

)

# Print the resulting dataset to see the output

print(last_measurement)

str(last_measurement)

# Add a new column 'quadrant' to classify each plot into one of the quadrants or other cases

last_measurement2 <- last_measurement %>%

mutate(

quadrant = case_when(

delta_beech > 0 & delta_maple > 0 ~ "B) Maple increase, beech increase", # Top right

delta_beech < 0 & delta_maple > 0 ~ "A) Maple increase, beech decrease", # Top left

delta_beech < 0 & delta_maple < 0 ~ "C) Maple decrease, beech decrease", # Bottom left

delta_beech > 0 & delta_maple < 0 ~ "D) Maple decrease, beech increase", # Bottom right

delta_maple < 0 & delta_beech == 0 ~ "E) Maple decrease, beech no change", # Special case (delta_maple < 0 and delta_beech == 0)

delta_maple > 0 & delta_beech == 0 ~ "F) Maple increase, beech no change", # Special case (delta_maple < 0 and delta_beech == 0)

delta_beech == 0 & delta_maple == 0 ~ "G) No change both species", # Special case (0, 0)

TRUE ~ "H) Other" # In case there are any other data points

)

)

# Count the number of plots in each renamed quadrant or other categories

quadrant_counts <- last_measurement2 %>%

count(quadrant)

# Print the quadrant counts

print(quadrant_counts)

# Count the number of plots in each quadrant or other categories

quadrant_counts <- last_measurement2 %>%

count(quadrant)

# Print the quadrant counts

print(quadrant_counts)

## Save the table to a CSV file

file_path <- "C:/Users/martigau/OneDrive - NRCan RNCan/Documents/Personnel/Travail/EcolEvol/NFI Data/last_measurement2.csv"

write.csv(last_measurement2, file = file_path)

write.csv2(last_measurement2, 'last_measurement2_fr.csv', row.names = FALSE)

# Create the plot with quadrant lines behind the points, size 2, and a panel border

ggplot(last_measurement, aes(x = delta_beech, y = delta_maple)) +

# Add the quadrant lines first so they are at the back

geom_vline(xintercept = 0, color = "black", linewidth = 0.5) + # Vertical line at x = 0

geom_hline(yintercept = 0, color = "black", linewidth = 0.5) + # Horizontal line at y = 0

# Add data points after the quadrant lines so that they are on top, size 2

geom_point(color = "black", fill = "green", size = 2, shape = 21) + # Black outline, gold fill, size 2 points

labs(

x = "Delta American beech (%)",

y = "Delta sugar maple (%)"

) +

theme_minimal() + # Minimal theme

theme(

panel.border = element_rect(color = "black", fill = NA, linewidth = 0.5), # Add a black border around the panel

#panel.grid = element_blank(), # Remove the grid lines

axis.ticks.y = element_line(color = "black", linewidth = 0.5), # Thicker y-axis ticks

axis.ticks.x = element_line(color = "black", linewidth = 0.5) # Thicker x-axis ticks

) +

# Add simplified quadrant labels (A, B, C, D) to each quadrant using annotate (without bold font)

annotate("text", x = -0.8, y = 0.8, label = "A)", size = 6) + # Top left

annotate("text", x = 0.8, y = 0.8, label = "B)", size = 6) + # Top right

annotate("text", x = -0.8, y = -0.2, label = "C)", size = 6) + # Bottom left (adjusted y = -0.25)

annotate("text", x = 0.8, y = -0.2, label = "D)", size = 6) # Bottom right (adjusted y = -0.25)

###########################################################

### FIGURE 2 - REVISED TO INCLUDE BEECH IN SECOND PANEL ###

###########################################################

library(ggplot2)

library(dplyr)

# Reshape the data to long format to make plotting easier

ecolevol_wide6_filtered_long <- ecolevol_wide6_filtered %>%

dplyr::select(meas_year, S1_pct_basal_area_ACER.SAH, S1_pct_basal_area_FAGU.GRA) %>%

pivot_longer(cols = starts_with("S1_pct_basal_area"),

names_to = "species",

values_to = "abundance")

# Plotting the mean linear regression line and 95% confidence interval for each species

ggplot(ecolevol_wide6_filtered_long, aes(x = meas_year, y = abundance)) +

geom_smooth(aes(color = species), method = "lm", se = TRUE, size = 1) + # Linear regression with CI

labs(x = "Measurement Year", y = "Abundance (%)") +

facet_wrap(~species, scales = "free_y", ncol = 2) + # Place plots side by side (2 columns)

theme_minimal() +

theme(

panel.border = element_rect(color = "black", fill = NA, linewidth = 0.5),

panel.grid = element_blank(),

axis.ticks.y = element_line(color = "black", linewidth = 0.5),

axis.ticks.x = element_line(color = "black", linewidth = 0.5),

axis.ticks.length = unit(0.2, "cm")

) +

scale_x_continuous(breaks = c(1970, 1980, 1990, 2000, 2010, 2020)) + # Specific x-axis labels

scale_y_continuous(limits = c(0, 1), breaks = seq(0, 1, by = 0.2)) # Y-axis limits and breaks

####################################################

### FIGURE 4 - EFFECT OF BEECH SAPLING ABUNDANCE ###

####################################################

# Effect of beech saplings

library(ggplot2)

ggplot(ecolevol_wide6_filtered, aes(x = S1_pct_basal_area_FAGU.GRA, y = S1_pct_basal_area_ACER.SAH)) +

geom_smooth(

method = "lm",

color = "forestgreen",

fill = "forestgreen",

se = TRUE,

fullrange = TRUE,

formula = y ~ pmax(0, x),

aes(ymin = pmax(0, after_stat(ymin)), ymax = after_stat(ymax))

) +

labs(x = "American beech abundance (%)", y = "Sugar maple abundance (%)") +

theme_minimal() +

theme(

panel.border = element_rect(color = "black", fill = NA, linewidth = 0.5),

panel.grid = element_blank(),

axis.ticks.y = element_line(color = "black", linewidth = 0.5),

axis.ticks.x = element_line(color = "black", linewidth = 0.5),

axis.ticks.length = unit(0.2, "cm")

) +

scale_y_continuous(limits = c(0, 1), breaks = seq(0, 1, by = 0.2)) +

scale_x_continuous(breaks = seq(0, 1, by = 0.2))

###############################################################

### FIGURE 2 - BOTH SUGAR MAPLE AND BEECH ON THE SAME PANEL ###

###############################################################

# Reshape the data to long format to make plotting easier

ecolevol_wide6_filtered_long <- ecolevol_wide6_filtered %>%

dplyr::select(meas_year, S1_pct_basal_area_ACER.SAH, S1_pct_basal_area_FAGU.GRA) %>%

pivot_longer(cols = starts_with("S1_pct_basal_area"),

names_to = "Species",

values_to = "Abundance")

# Plotting both species in the same panel with regression lines and 95% confidence intervals

ggplot(ecolevol_wide6_filtered_long, aes(x = meas_year, y = Abundance, color = Species, fill = Species)) +

geom_smooth(method = "lm", se = TRUE, linewidth = 1, alpha = 0.2) + # Linear regression with CI, alpha for transparency

labs(x = "Measurement year", y = "Relative abundance (%)") +

scale_color_manual(values = c("S1_pct_basal_area_ACER.SAH" = "#DC143C",

"S1_pct_basal_area_FAGU.GRA" = "#4169E1"),

labels = c("Sugar maple", "American beech")) + # Custom colors and labels

scale_fill_manual(values = c("S1_pct_basal_area_ACER.SAH" = "#DC143C",

"S1_pct_basal_area_FAGU.GRA" = "#4169E1")) + # Match fill color with line color

theme_minimal() +

theme(

panel.border = element_rect(color = "black", fill = NA, linewidth = 0.5),

panel.grid = element_blank(),

axis.ticks.y = element_line(color = "black", linewidth = 0.5),

axis.ticks.x = element_line(color = "black", linewidth = 0.5),

axis.ticks.length = unit(0.2, "cm"),

legend.position = c(0.5, 0.8), # Position the legend inside the plot at the top-right corner

legend.background = element_rect(fill = alpha("white", 0.5)), # Semi-transparent background for the legend

legend.title = element_blank() # Optional: Remove legend title if desired

) +

scale_x_continuous(breaks = c(1970, 1980, 1990, 2000, 2010, 2020)) + # Specific x-axis labels

scale_y_continuous(limits = c(0, 1), breaks = seq(0, 1, by = 0.2)) + # Y-axis limits and breaks

guides(fill = "none") # Hide the duplicate fill legend

###########################################################

### DESCRIPTIVE STATISTICS OF SITE AND STAND CONDITIONS ###

###########################################################

# Load dataset

ecolevol_wide6 <- fread("C:/Users/martigau/OneDrive - NRCan RNCan/Documents/Personnel/Travail/EcolEvol/NFI Data/ecolevol_wide6.csv")

colnames(ecolevol_wide6)

str(ecolevol_wide6)

print(ecolevol_wide6)

# Sort out data to see range of harvest intensity

ecolevol_wide4_sorted <- ecolevol_wide4[order(ecolevol_wide4$treatment_pct.y), ]

unique(ecolevol_wide4_sorted$treatment_pct.y)

# Total 134 harvested plots, ranges from 5% to 92% harvesting

# Keep selected variables

harvest3 <- ecolevol_wide4 %>%

dplyr :: select ("magp_site_id", "meas_num", "meas_year",

"silv", "category", "treatment_type.x", "treatment_type.y",

"treatment_year.x", "treatment_year.y",

"treatment_year2", "time_since_trt",

"treatment_intensity", "treatment_pct.y", "notes", "sources")

colnames(harvest3)

# Create frequency table to know when treatments occurred

# Process the data

frequency_table_all <- harvest3 %>%

# Select relevant columns and remove duplicates

distinct(magp_site_id, silv, treatment_year.x, .keep_all = TRUE) %>%

# Group by treatment_type and treatment_year

group_by(silv, treatment_year.x) %>%

# Count unique sites

summarise(site_count = n_distinct(magp_site_id), .groups = 'drop')

# Print the frequency table

print(n = 100, frequency_table_all)

# Save the table to a CSV file

file_path <- "C:/Users/martigau/OneDrive - NRCan RNCan/Documents/Personnel/Travail/EcolEvol/NFI Data/frequency_table_all.csv"

write.csv(frequency_table_all, file = file_path)

write.csv2(frequency_table_all, 'frequency_table_all_fr.csv', row.names = FALSE)

# Count the number of treatments per site

treatment_counts <- harvest3 %>%

group_by(magp_site_id) %>%

summarise(

treatment_count = n_distinct(treatment_type.y), # Count distinct treatment types

.groups = 'drop'

)

# Identify sites with more than one treatment

sites_with_multiple_treatments <- treatment_counts %>%

filter(treatment_count > 1)

# Extract details for sites with multiple treatments

treatments_details <- ecolevol_wide4%>%

filter(magp_site_id %in% sites_with_multiple_treatments$magp_site_id) %>%

arrange(magp_site_id, treatment_year.y)

# Print the details of treatments for sites with two treatments

print(treatments_details, n = 27)

unique(treatments_details$magp_site_id)

# Save the table to a CSV file

file_path <- "C:/Users/martigau/OneDrive - NRCan RNCan/Documents/Personnel/Travail/EcolEvol/NFI Data/treatments_details.csv"

write.csv(treatments_details, file = file_path)

write.csv2(treatments_details, 'treatments_details_fr.csv', row.names = FALSE)

# Load necessary libraries

library(dplyr)

library(tidyr)

# Create a summary table with treatment combinations and associated site IDs

treatment_combinations <- ecolevol_wide4%>%

# Group by site ID and gather all treatments for each site

group_by(magp_site_id) %>%

summarise(treatments = paste(treatment_type.y, collapse = ", "), .groups = 'drop') %>%

# Group by treatment combinations to find unique combinations and count occurrences

group_by(treatments) %>%

summarise(

site_ids = paste(magp_site_id, collapse = ", "), # Combine site IDs for each treatment combination

site_count = n(), # Count the number of sites for each combination

.groups = 'drop'

) %>%

# Arrange the table for better readability

arrange(desc(site_count))

# Print the summary table

print(treatment_combinations)

# Save the table to a CSV file

file_path <- "C:/Users/martigau/OneDrive - NRCan RNCan/Documents/Personnel/Travail/EcolEvol/NFI Data/treatment_combinations.csv"

write.csv(treatment_combinations, file = file_path)

write.csv2(treatment_combinations, 'treatment_combinations_fr.csv', row.names = FALSE)

# Descriptive statistics of harvesting intensity

# 1) Filter for rows with treatment_intensity values

harvest_intensity <- harvest3 %>%

filter(treatment_pct.y > 0)

# Compute min, mean, max, and sample size (0,05%, 0,53%, 0,92%, n = 134)

harvest_intensity_stats <- harvest_intensity %>%

distinct(magp_site_id, .keep_all = TRUE) %>%

summarize(

min_value = min(treatment_pct.y, na.rm = TRUE),

max_value = max(treatment_pct.y, na.rm = TRUE),

mean_value = mean(treatment_pct.y, na.rm = TRUE),

sd_value = sd(treatment_pct.y, na.rm = TRUE),

sample_size = n(),

se_value = sd_value / sqrt(sample_size)

)

print(harvest_intensity_stats)

file_path <- "C:/Users/martigau/OneDrive - NRCan RNCan/Documents/Personnel/Travail/EcolEvol/NFI Data/harvest_intensity_stats.csv"

write.csv(harvest_intensity_stats, file = file_path)

write.csv2(harvest_intensity_stats, 'harvest_intensity_stats_fr.csv', row.names = FALSE)

# How many between 5-40%, 41-80%, and greater than 80%

harvest_intensity_freq <- harvest_intensity %>%

distinct(magp_site_id, .keep_all = TRUE) %>%

mutate(

category = case_when(

treatment_pct.y >= 0.05 & treatment_pct.y <= 0.4099 ~ "Low",

treatment_pct.y >= 0.410 & treatment_pct.y <= 0.8 ~ "High",

treatment_pct.y > 0.8 ~ "Very high",

TRUE ~ "Other"

)

) %>%

count(category)

# View the result

print(harvest_intensity_freq)

# Calculate treatment year information

harvest_year <- ecolevol_wide4 %>%

select(-V1) %>%

filter(!treatment_year.y == "NA")

nrow(ecolevol_wide4)

nrow(harvest_year)

colnames(harvest_year)

harvest_year2 <- harvest_year %>%

distinct(magp_site_id, .keep_all = TRUE) %>%

summarize(

min_value = min(treatment_year.y, na.rm = TRUE),

max_value = max(treatment_year.y, na.rm = TRUE),

mean_value = mean(treatment_year.y, na.rm = TRUE),

sd_value = sd(treatment_year.y, na.rm = TRUE),

sample_size = n(),

se_value = sd_value / sqrt(sample_size)

)

print(harvest_year2)

file_path <- "C:/Users/martigau/OneDrive - NRCan RNCan/Documents/Personnel/Travail/EcolEvol/NFI Data/harvest_year2.csv"

write.csv(harvest_year2, file = file_path)

write.csv2(harvest_year2, 'harvest_year2_fr.csv', row.names = FALSE)

# Time since treatment information

harvest_resp <- harvest_year %>%

distinct(magp_site_id, .keep_all = TRUE) %>%

summarize(

min_value = min(time_since_trt, na.rm = TRUE),

max_value = max(time_since_trt, na.rm = TRUE),

mean_value = mean(time_since_trt, na.rm = TRUE),

sd_value = sd(time_since_trt, na.rm = TRUE),

sample_size = n(),

se_value = sd_value / sqrt(sample_size)

)

print(harvest_resp)

file_path <- "C:/Users/martigau/OneDrive - NRCan RNCan/Documents/Personnel/Travail/EcolEvol/NFI Data/harvest_resp.csv"

write.csv(harvest_resp, file = file_path)

write.csv2(harvest_resp, 'harvest_resp_fr.csv', row.names = FALSE)

# Compute min, max, mean, and median of 'time_since_trt' for each 'magp_site_id'

time_since_trt_stats <- harvest_intensity %>%

filter(time_since_trt > 0) %>% # Only include rows where 'time_since_trt' is greater than 0

group_by(magp_site_id) %>% # Group by 'magp_site_id'

summarize(

min_time_since_trt = min(time_since_trt, na.rm = TRUE),

max_time_since_trt = max(time_since_trt, na.rm = TRUE),

mean_time_since_trt = mean(time_since_trt, na.rm = TRUE),

median_time_since_trt = median(time_since_trt, na.rm = TRUE) # Adding median calculation

)

print(time_since_trt_stats)

file_path <- "C:/Users/martigau/OneDrive - NRCan RNCan/Documents/Personnel/Travail/EcolEvol/NFI Data/time_since_trt_stats.csv"

write.csv(time_since_trt_stats, file = file_path)

write.csv2(time_since_trt_stats, 'time_since_trt_stats_fr.csv', row.names = FALSE)

# Compute min, max, mean, and median of 'time_since_trt' for each 'magp_site_id'

time_since_trt_stats_cat <- harvest_intensity %>%

filter(time_since_trt > 0) %>% # Only include rows where 'time_since_trt' is greater than 0

group_by(category) %>% # Group by 'magp_site_id'

summarize(

min_time_since_trt = min(time_since_trt, na.rm = TRUE),

max_time_since_trt = max(time_since_trt, na.rm = TRUE),

mean_time_since_trt = mean(time_since_trt, na.rm = TRUE),

median_time_since_trt = median(time_since_trt, na.rm = TRUE) # Adding median calculation

)

print(time_since_trt_stats_cat)

file_path <- "C:/Users/martigau/OneDrive - NRCan RNCan/Documents/Personnel/Travail/EcolEvol/NFI Data/time_since_trt_stats.csv"

write.csv(time_since_trt_stats, file = file_path)

write.csv2(time_since_trt_stats, 'time_since_trt_stats_fr.csv', row.names = FALSE)

# Compute the overall mean and median of min, max, and mean across all magp_site_id

overall_stats <- time_since_trt_stats %>%

summarize(

mean_min_time_since_trt = mean(min_time_since_trt, na.rm = TRUE),

mean_max_time_since_trt = mean(max_time_since_trt, na.rm = TRUE),

mean_mean_time_since_trt = mean(mean_time_since_trt, na.rm = TRUE),

mean_median_time_since_trt = mean(median_time_since_trt, na.rm = TRUE), # Mean of medians

median_min_time_since_trt = median(min_time_since_trt, na.rm = TRUE),

median_max_time_since_trt = median(max_time_since_trt, na.rm = TRUE),

median_mean_time_since_trt = median(mean_time_since_trt, na.rm = TRUE),

median_median_time_since_trt = median(median_time_since_trt, na.rm = TRUE) # Median of medians

)

print(overall_stats)

file_path <- "C:/Users/martigau/OneDrive - NRCan RNCan/Documents/Personnel/Travail/EcolEvol/NFI Data/overall_stats.csv"

write.csv(overall_stats, file = file_path)

write.csv2(overall_stats, 'overall_stats_stats_fr.csv', row.names = FALSE)

###########################################################

#### DESCRIPTIVE STATISTICS PRIOR TO HARVEST ##############

###########################################################

# Load revised dataset

ecolevol_wide6 <- fread("C:/Users/martigau/OneDrive - NRCan RNCan/Documents/Personnel/Travail/EcolEvol/NFI Data/ecolevol_wide6.csv")

colnames(ecolevol_wide6)

ecolevol_wide7 <- ecolevol_wide6 %>%

dplyr::select(-V1)

#ecolevol_wide8 <- ecolevol_wide7 %>%

#dplyr::select(-V1.y)

colnames(ecolevol_wide7)

###### Use initial_L1_pct_ba_ACER.SAH instead of L1_initial

# Add first measurement variable since not all sites were measured at meas_num = 0

descript_stats <- ecolevol_wide6 %>%

arrange(magp_site_id, meas_num) %>%

group_by(magp_site_id) %>%

mutate(

L1_initial_density = first(L1_total_density_sum),

L1_initial_density_ACER.SAH = first(L1_density_ACER.SAH),

L1_initial_density_FAGU.GRA = first(L1_density_FAGU.GRA),

L1_initial_pct_density_ACER.SAH = first(L1_pct_density_ACER.SAH),

L1_initial_pct_density_FAGU.GRA = first(L1_pct_density_FAGU.GRA),

S1_initial_density = first(S1_total_density_sum),

S1_initial_density_ACER.SAH = first(S1_density_ACER.SAH),

S1_initial_density_FAGU.GRA = first(S1_density_FAGU.GRA),

S1_initial_pct_density_ACER.SAH = first(S1_pct_density_ACER.SAH),

S1_initial_pct_density_FAGU.GRA = first(S1_pct_density_FAGU.GRA),

L1_initial_ba = first(L1_total_basal_area_sum),

L1_initial_ba_ACER.SAH = first(L1_ba_ACER.SAH),

L1_initial_ba_FAGU.GRA = first(L1_ba_FAGU.GRA),

L1_initial_pct_ba_ACER.SAH = first(L1_pct_basal_area_ACER.SAH),

L1_initial_pct_ba_FAGU.GRA = first(L1_pct_basal_area_FAGU.GRA),

S1_initial_ba = first(S1_total_basal_area_sum),

S1_initial_ba_ACER.SAH = first(S1_ba_ACER.SAH),

S1_initial_ba_FAGU.GRA = first(S1_ba_FAGU.GRA),

S1_initial_pct_ba_ACER.SAH = first(S1_pct_basal_area_ACER.SAH),

S1_initial_pct_ba_FAGU.GRA = first(S1_pct_basal_area_FAGU.GRA)

) %>%

ungroup()

nrow(descript_stats)

print(descript_stats)

str(descript_stats)

unique(descript_stats$L1_initial_pct_ba_ACER.SAH)

colnames(descript_stats)

## Save the table to a CSV file

file_path <- "C:/Users/martigau/OneDrive - NRCan RNCan/Documents/Personnel/Travail/EcolEvol/NFI Data/descript_stats.csv"

write.csv(descript_stats, file = file_path)

write.csv2(descript_stats, 'descript_stats_fr.csv', row.names = FALSE)

# STEP 1) L1 & S1 INITIAL STAND DENSITY

# Compute stand density stats, standard error, and sample size

initial_density <- descript_stats %>%

dplyr::distinct(magp_site_id, .keep_all = TRUE) %>%

dplyr::summarize(

mean_L1 = mean(L1_initial_density, na.rm = TRUE),

sd_L1 = sd(L1_initial_density, na.rm = TRUE),

se_L1 = sd_L1 / sqrt(n()),

mean_S1 = mean(S1_initial_density, na.rm = TRUE),

sd_S1 = sd(S1_initial_density, na.rm = TRUE),

se_S1 = sd_S1 / sqrt(n()),

sample_size = n(),

.groups = 'drop'

)

print(initial_density)

## Save the table to a CSV file

file_path <- "C:/Users/martigau/OneDrive - NRCan RNCan/Documents/Personnel/Travail/EcolEvol/NFI Data/initial_density.csv"

write.csv(initial_density, file = file_path)

write.csv2(initial_density, 'initial_density_fr.csv', row.names = FALSE)

# STEP 2) L1 & S1 INITIAL STAND DENSITY BY TREATMENT

# Compute stand density stats, standard error, and sample size

initial_density_trt <- descript_stats %>%

distinct(magp_site_id, .keep_all = TRUE) %>%

group_by(silv) %>%

summarize(

mean_L1 = mean(L1_initial_density, na.rm = TRUE),

sd_L1 = sd(L1_initial_density, na.rm = TRUE),

se_L1 = sd_L1 / sqrt(n()),

mean_S1 = mean(S1_initial_density, na.rm = TRUE),

sd_S1 = sd(S1_initial_density, na.rm = TRUE),

se_S1 = sd_S1 / sqrt(n()),

sample_size = n(),

.groups = 'drop'

)

print(initial_density_trt)

## Save the table to a CSV file

file_path <- "C:/Users/martigau/OneDrive - NRCan RNCan/Documents/Personnel/Travail/EcolEvol/NFI Data/initial_density_trt.csv"

write.csv(initial_density_trt, file = file_path)

write.csv2(initial_density_trt, 'initial_density_trt_fr.csv', row.names = FALSE)

# STEP 2B) L1 & S1 INITIAL STAND DENSITY BY ECOZONE

# Compute stand density stats, standard error, and sample size

initial_density_eco <- descript_stats %>%

distinct(magp_site_id, .keep_all = TRUE) %>%

group_by(ecozone) %>%

summarize(

mean_L1 = mean(L1_initial_density, na.rm = TRUE),

sd_L1 = sd(L1_initial_density, na.rm = TRUE),

se_L1 = sd_L1 / sqrt(n()),

mean_S1 = mean(S1_initial_density, na.rm = TRUE),

sd_S1 = sd(S1_initial_density, na.rm = TRUE),

se_S1 = sd_S1 / sqrt(n()),

sample_size = n(),

.groups = 'drop'

)

print(initial_density_eco)

## Save the table to a CSV file

file_path <- "C:/Users/martigau/OneDrive - NRCan RNCan/Documents/Personnel/Travail/EcolEvol/NFI Data/initial_density_eco.csv"

write.csv(initial_density_eco, file = file_path)

write.csv2(initial_density_eco, 'initial_density_eco_fr.csv', row.names = FALSE)

# STEP 3) L1 & S1 INITIAL STAND DENSITY BY SPECIES AND TREATMENT

# Compute stand density stats, standard error, and sample size

initial_density_sp <- descript_stats %>%

distinct(magp_site_id, .keep_all = TRUE) %>%

group_by(silv) %>%

summarize(

mean_L1_AS = mean(L1_initial_density_ACER.SAH, na.rm = TRUE),

sd_L1_AS = sd(L1_initial_density_ACER.SAH, na.rm = TRUE),

se_L1_AS = sd_L1_AS / sqrt(n()),

mean_S1_AS = mean(S1_initial_density_ACER.SAH, na.rm = TRUE),

sd_S1_AS = sd(S1_initial_density_ACER.SAH, na.rm = TRUE),

se_S1_AS = sd_S1_AS / sqrt(n()),

mean_L1_FG = mean(L1_initial_density_FAGU.GRA, na.rm = TRUE),

sd_L1_FG = sd(L1_initial_density_FAGU.GRA, na.rm = TRUE),

se_L1_FG = sd_L1_FG / sqrt(n()),

mean_S1_FG = mean(S1_initial_density_FAGU.GRA, na.rm = TRUE),

sd_S1_FG = sd(S1_initial_density_FAGU.GRA, na.rm = TRUE),

se_S1_FG = sd_S1_FG / sqrt(n()),

sample_size = n(),

.groups = 'drop'

)

print(initial_density_sp)

## Save the table to a CSV file

file_path <- "C:/Users/martigau/OneDrive - NRCan RNCan/Documents/Personnel/Travail/EcolEvol/NFI Data/initial_density_sp.csv"

write.csv(initial_density_sp, file = file_path)

write.csv2(initial_density_sp, 'initial_density_sp_fr.csv', row.names = FALSE)

# STEP 3B) L1 & S1 INITIAL STAND DENSITY BY SPECIES AND ECOZONE

# Compute stand density stats, standard error, and sample size

initial_density_sp_eco <- descript_stats %>%

distinct(magp_site_id, .keep_all = TRUE) %>%

group_by(ecozone) %>%

summarize(

mean_L1_AS = mean(L1_initial_density_ACER.SAH, na.rm = TRUE),

sd_L1_AS = sd(L1_initial_density_ACER.SAH, na.rm = TRUE),

se_L1_AS = sd_L1_AS / sqrt(n()),

mean_S1_AS = mean(S1_initial_density_ACER.SAH, na.rm = TRUE),

sd_S1_AS = sd(S1_initial_density_ACER.SAH, na.rm = TRUE),

se_S1_AS = sd_S1_AS / sqrt(n()),

mean_L1_FG = mean(L1_initial_density_FAGU.GRA, na.rm = TRUE),

sd_L1_FG = sd(L1_initial_density_FAGU.GRA, na.rm = TRUE),

se_L1_FG = sd_L1_FG / sqrt(n()),

mean_S1_FG = mean(S1_initial_density_FAGU.GRA, na.rm = TRUE),

sd_S1_FG = sd(S1_initial_density_FAGU.GRA, na.rm = TRUE),

se_S1_FG = sd_S1_FG / sqrt(n()),

sample_size = n(),

.groups = 'drop'

)

print(initial_density_sp_eco)

## Save the table to a CSV file

file_path <- "C:/Users/martigau/OneDrive - NRCan RNCan/Documents/Personnel/Travail/EcolEvol/NFI Data/initial_density_sp_eco.csv"

write.csv(initial_density_sp_eco, file = file_path)

write.csv2(initial_density_sp_eco, 'initial_density_sp_eco_fr.csv', row.names = FALSE)

# STEP 4) L1 & S1 INITIAL BASAL AREA

# Compute stand BA stats, standard error, and sample size with range (min, max)

initial_ba <- descript_stats %>%

distinct(magp_site_id, .keep_all = TRUE) %>%

summarize(

mean_L1 = mean(L1_initial_ba, na.rm = TRUE),

sd_L1 = sd(L1_initial_ba, na.rm = TRUE),

se_L1 = sd_L1 / sqrt(n()),

min_L1 = min(L1_initial_ba, na.rm = TRUE), # Minimum value for L1

max_L1 = max(L1_initial_ba, na.rm = TRUE), # Maximum value for L1

mean_S1 = mean(S1_initial_ba, na.rm = TRUE),

sd_S1 = sd(S1_initial_ba, na.rm = TRUE),

se_S1 = sd_S1 / sqrt(n()),

min_S1 = min(S1_initial_ba, na.rm = TRUE), # Minimum value for S1

max_S1 = max(S1_initial_ba, na.rm = TRUE), # Maximum value for S1

sample_size = n(),

.groups = 'drop'

)

print(initial_ba)

## Save the table to a CSV file

file_path <- "C:/Users/martigau/OneDrive - NRCan RNCan/Documents/Personnel/Travail/EcolEvol/NFI Data/initial_ba.csv"

write.csv(initial_ba, file = file_path)

write.csv2(initial_ba, 'initial_ba_fr.csv', row.names = FALSE)

# STEP 5) L1 & S1 INITIAL BASAL AREA BY TREATMENT

# Compute stand BA stats, standard error, and sample size

initial_ba_trt <- descript_stats %>%

distinct(magp_site_id, .keep_all = TRUE) %>%

group_by(silv) %>%

summarize(

mean_L1 = mean(L1_initial_ba, na.rm = TRUE),

sd_L1 = sd(L1_initial_ba, na.rm = TRUE),

se_L1 = sd_L1 / sqrt(n()),

mean_S1 = mean(S1_initial_ba, na.rm = TRUE),

sd_S1 = sd(S1_initial_ba, na.rm = TRUE),

se_S1 = sd_S1 / sqrt(n()),

sample_size = n(),

.groups = 'drop'

)

print(initial_ba_trt)

## Save the table to a CSV file

file_path <- "C:/Users/martigau/OneDrive - NRCan RNCan/Documents/Personnel/Travail/EcolEvol/NFI Data/initial_ba_trt.csv"

write.csv(initial_ba_trt, file = file_path)

write.csv2(initial_ba_trt, 'initial_ba_trt_fr.csv', row.names = FALSE)

# STEP 5B) L1 & S1 INITIAL BASAL AREA BY ECOZONE

# Compute stand BA stats, standard error, and sample size

initial_ba_eco <- descript_stats %>%

distinct(magp_site_id, .keep_all = TRUE) %>%

group_by(ecozone) %>%

summarize(

mean_L1 = mean(L1_initial_ba, na.rm = TRUE),

sd_L1 = sd(L1_initial_ba, na.rm = TRUE),

se_L1 = sd_L1 / sqrt(n()),

mean_S1 = mean(S1_initial_ba, na.rm = TRUE),

sd_S1 = sd(S1_initial_ba, na.rm = TRUE),

se_S1 = sd_S1 / sqrt(n()),

sample_size = n(),

.groups = 'drop'

)

print(initial_ba_eco)

## Save the table to a CSV file

file_path <- "C:/Users/martigau/OneDrive - NRCan RNCan/Documents/Personnel/Travail/EcolEvol/NFI Data/initial_ba_eco.csv"

write.csv(initial_ba_eco, file = file_path)

write.csv2(initial_ba_eco, 'initial_ba_eco_fr.csv', row.names = FALSE)

# STEP 6) L1 & S1 INITIAL STAND BASAL AREA BY SPECIES AND TREATMENT

# Compute stand density stats, standard error, and sample size

initial_ba_sp <- descript_stats %>%

distinct(magp_site_id, .keep_all = TRUE) %>%

group_by(silv) %>%

summarize(

mean_L1_AS = mean(L1_initial_ba_ACER.SAH, na.rm = TRUE),

sd_L1_AS = sd(L1_initial_ba_ACER.SAH, na.rm = TRUE),

se_L1_AS = sd_L1_AS / sqrt(n()),

mean_S1_AS = mean(S1_initial_ba_ACER.SAH, na.rm = TRUE),

sd_S1_AS = sd(S1_initial_ba_ACER.SAH, na.rm = TRUE),

se_S1_AS = sd_S1_AS / sqrt(n()),

mean_L1_FG = mean(L1_initial_ba_FAGU.GRA, na.rm = TRUE),

sd_L1_FG = sd(L1_initial_ba_FAGU.GRA, na.rm = TRUE),

se_L1_FG = sd_L1_FG / sqrt(n()),

mean_S1_FG = mean(S1_initial_ba_FAGU.GRA, na.rm = TRUE),

sd_S1_FG = sd(S1_initial_ba_FAGU.GRA, na.rm = TRUE),

se_S1_FG = sd_S1_FG / sqrt(n()),

sample_size = n(),

.groups = 'drop'

)

print(initial_ba_sp)

## Save the table to a CSV file

file_path <- "C:/Users/martigau/OneDrive - NRCan RNCan/Documents/Personnel/Travail/EcolEvol/NFI Data/initial_ba_sp.csv"

write.csv(initial_ba_sp, file = file_path)

write.csv2(initial_ba_sp, 'initial_ba_sp_fr.csv', row.names = FALSE)

# STEP 6B) L1 & S1 INITIAL STAND BASAL AREA BY SPECIES AND ECOZONE

# Compute stand density stats, standard error, and sample size

initial_ba_sp_eco <- descript_stats %>%

distinct(magp_site_id, .keep_all = TRUE) %>%

group_by(ecozone) %>%

summarize(

mean_L1_AS = mean(L1_initial_ba_ACER.SAH, na.rm = TRUE),

sd_L1_AS = sd(L1_initial_ba_ACER.SAH, na.rm = TRUE),

se_L1_AS = sd_L1_AS / sqrt(n()),

mean_S1_AS = mean(S1_initial_ba_ACER.SAH, na.rm = TRUE),

sd_S1_AS = sd(S1_initial_ba_ACER.SAH, na.rm = TRUE),

se_S1_AS = sd_S1_AS / sqrt(n()),

mean_L1_FG = mean(L1_initial_ba_FAGU.GRA, na.rm = TRUE),

sd_L1_FG = sd(L1_initial_ba_FAGU.GRA, na.rm = TRUE),

se_L1_FG = sd_L1_FG / sqrt(n()),

mean_S1_FG = mean(S1_initial_ba_FAGU.GRA, na.rm = TRUE),

sd_S1_FG = sd(S1_initial_ba_FAGU.GRA, na.rm = TRUE),

se_S1_FG = sd_S1_FG / sqrt(n()),

sample_size = n(),

.groups = 'drop'

)

print(initial_ba_sp_eco)

## Save the table to a CSV file

file_path <- "C:/Users/martigau/OneDrive - NRCan RNCan/Documents/Personnel/Travail/EcolEvol/NFI Data/initial_ba_sp_eco.csv"

write.csv(initial_ba_sp_eco, file = file_path)

write.csv2(initial_ba_sp_eco, 'initial_ba_sp_eco_fr.csv', row.names = FALSE)

# STEP 7) L1 & S1 INITIAL PERCENT BASAL AREA BY SPECIES

str(descript_stats)

# Compute stats, standard error, and sample size

initial_dep_var <- descript_stats %>%

distinct(magp_site_id, .keep_all = TRUE) %>%

summarize(

mean_L1 = mean(initial_L1_pct_ba_ACER.SAH, na.rm = TRUE),

sd_L1 = sd(initial_L1_pct_ba_ACER.SAH, na.rm = TRUE),

se_L1 = sd_L1 / sqrt(n()),

min_pctL1 = min(initial_L1_pct_ba_ACER.SAH, na.rm = TRUE),

max_pctL1 = max(initial_L1_pct_ba_ACER.SAH, na.rm = TRUE),

mean_S1 = mean(initial_S1_pct_ba_ACER.SAH, na.rm = TRUE),

sd_S1 = sd(initial_S1_pct_ba_ACER.SAH, na.rm = TRUE),

se_S1 = sd_S1 / sqrt(n()),

min_pctS1 = min(initial_S1_pct_ba_ACER.SAH, na.rm = TRUE),

max_pctS1 = max(initial_S1_pct_ba_ACER.SAH, na.rm = TRUE),

mean_L1_FG = mean(initial_L1_pct_ba_FAGU.GRA, na.rm = TRUE),

sd_L1_FG = sd(initial_L1_pct_ba_FAGU.GRA, na.rm = TRUE),

se_L1_FG = sd_L1_FG / sqrt(n()),

min_pctL1_FG = min(initial_L1_pct_ba_FAGU.GRA, na.rm = TRUE),

max_pctL1_FG = max(initial_L1_pct_ba_FAGU.GRA, na.rm = TRUE),

mean_S1_FG = mean(initial_S1_pct_ba_FAGU.GRA, na.rm = TRUE),

sd_S1_FG = sd(initial_S1_pct_ba_FAGU.GRA, na.rm = TRUE),

se_S1_FG = sd_S1_FG / sqrt(n()),

min_pctS1_FG = min(initial_S1_pct_ba_FAGU.GRA, na.rm = TRUE),

max_pctS1_FG = max(initial_S1_pct_ba_FAGU.GRA, na.rm = TRUE),

sample_size = n(),

.groups = 'drop'

)

print(initial_dep_var)

## Save the table to a CSV file

file_path <- "C:/Users/martigau/OneDrive - NRCan RNCan/Documents/Personnel/Travail/EcolEvol/NFI Data/initial_dep_var.csv"

write.csv(initial_dep_var, file = file_path)

write.csv2(initial_dep_var, 'initial_dep_var_fr.csv', row.names = FALSE)

# STEP 8) INITIAL L1 & S1 PERCENT BASAL AREA BY SPECIES AND TREATMENT

str(descript_stats)

# Compute stand density stats, standard error, and sample size

initial_dep_var_trt <- descript_stats %>%

distinct(magp_site_id, .keep_all = TRUE) %>%

group_by(silv) %>%

summarize(

mean_L1 = mean(initial_L1_pct_ba_ACER.SAH, na.rm = TRUE),

sd_L1 = sd(initial_L1_pct_ba_ACER.SAH, na.rm = TRUE),

se_L1 = sd_L1 / sqrt(n()),

min_pctL1 = min(initial_L1_pct_ba_ACER.SAH, na.rm = TRUE),

max_pctL1 = max(initial_L1_pct_ba_ACER.SAH, na.rm = TRUE),

mean_S1 = mean(initial_S1_pct_ba_ACER.SAH, na.rm = TRUE),

sd_S1 = sd(initial_S1_pct_ba_ACER.SAH, na.rm = TRUE),

se_S1 = sd_S1 / sqrt(n()),

min_pctS1 = min(initial_S1_pct_ba_ACER.SAH, na.rm = TRUE),

max_pctS1 = max(initial_S1_pct_ba_ACER.SAH, na.rm = TRUE),

mean_L1_FG = mean(initial_L1_pct_ba_FAGU.GRA, na.rm = TRUE),

sd_L1_FG = sd(initial_L1_pct_ba_FAGU.GRA, na.rm = TRUE),

se_L1_FG = sd_L1_FG / sqrt(n()),

min_pctL1_FG = min(initial_L1_pct_ba_FAGU.GRA, na.rm = TRUE),

max_pctL1_FG = max(initial_L1_pct_ba_FAGU.GRA, na.rm = TRUE),

mean_S1_FG = mean(initial_S1_pct_ba_FAGU.GRA, na.rm = TRUE),

sd_S1_FG = sd(initial_S1_pct_ba_FAGU.GRA, na.rm = TRUE),

se_S1_FG = sd_S1_FG / sqrt(n()),

min_pctS1_FG = min(initial_S1_pct_ba_FAGU.GRA, na.rm = TRUE),

max_pctS1_FG = max(initial_S1_pct_ba_FAGU.GRA, na.rm = TRUE),

sample_size = n(),

.groups = 'drop'

)

print(initial_dep_var_trt)

## Save the table to a CSV file

file_path <- "C:/Users/martigau/OneDrive - NRCan RNCan/Documents/Personnel/Travail/EcolEvol/NFI Data/initial_dep_var_trt.csv"

write.csv(initial_dep_var_trt, file = file_path)

write.csv2(initial_dep_var_trt, 'initial_dep_var_trt_fr.csv', row.names = FALSE)

# STEP 8B) INITIAL L1 & S1 PERCENT BASAL AREA BY SPECIES AND ECOZONE

str(descript_stats)

# Compute stand density stats, standard error, and sample size

initial_dep_var_eco <- descript_stats %>%

distinct(magp_site_id, .keep_all = TRUE) %>%

group_by(ecozone) %>%

summarize(

mean_L1 = mean(initial_L1_pct_ba_ACER.SAH, na.rm = TRUE),

sd_L1 = sd(initial_L1_pct_ba_ACER.SAH, na.rm = TRUE),

se_L1 = sd_L1 / sqrt(n()),

min_pctL1 = min(initial_L1_pct_ba_ACER.SAH, na.rm = TRUE),

max_pctL1 = max(initial_L1_pct_ba_ACER.SAH, na.rm = TRUE),

mean_S1 = mean(initial_S1_pct_ba_ACER.SAH, na.rm = TRUE),

sd_S1 = sd(initial_S1_pct_ba_ACER.SAH, na.rm = TRUE),

se_S1 = sd_S1 / sqrt(n()),

min_pctS1 = min(initial_S1_pct_ba_ACER.SAH, na.rm = TRUE),

max_pctS1 = max(initial_S1_pct_ba_ACER.SAH, na.rm = TRUE),

mean_L1_FG = mean(initial_L1_pct_ba_FAGU.GRA, na.rm = TRUE),

sd_L1_FG = sd(initial_L1_pct_ba_FAGU.GRA, na.rm = TRUE),

se_L1_FG = sd_L1_FG / sqrt(n()),

min_pctL1_FG = min(initial_L1_pct_ba_FAGU.GRA, na.rm = TRUE),

max_pctL1_FG = max(initial_L1_pct_ba_FAGU.GRA, na.rm = TRUE),

mean_S1_FG = mean(initial_S1_pct_ba_FAGU.GRA, na.rm = TRUE),

sd_S1_FG = sd(initial_S1_pct_ba_FAGU.GRA, na.rm = TRUE),

se_S1_FG = sd_S1_FG / sqrt(n()),

min_pctS1_FG = min(initial_S1_pct_ba_FAGU.GRA, na.rm = TRUE),

max_pctS1_FG = max(initial_S1_pct_ba_FAGU.GRA, na.rm = TRUE),

sample_size = n(),

.groups = 'drop'

)

print(initial_dep_var_eco)

## Save the table to a CSV file

file_path <- "C:/Users/martigau/OneDrive - NRCan RNCan/Documents/Personnel/Travail/EcolEvol/NFI Data/initial_dep_var_eco.csv"

write.csv(initial_dep_var_eco, file = file_path)

write.csv2(initial_dep_var_eco, 'initial_dep_var_eco_fr.csv', row.names = FALSE)

################################################

### DESCRIPTION OF MEASUREMENTS BY ECOZONE #####

################################################

# How many plots per ecozone and data source?

plots_by_ecozone <- ecolevol_wide4 %>%

select(-V1) %>%

group_by(ecozone, source) %>%

summarize(count_magp_site_id = n_distinct(magp_site_id), .groups = 'drop')

print(plots_by_ecozone)

# Plot measurement number and year by ecozone

num_by_ecozone <- ecolevol_wide4 %>%

group_by(ecozone, sources) %>%

summarize(count_meas_num = n_distinct(magp_site_id), .groups = 'drop')

print(num_by_ecozone)

# Step 1: Count how many times each plot has been measured for each ecozone and source

count_measures <- ecolevol_wide4 %>%

group_by(ecozone, sources, magp_site_id) %>%

summarise(measurement_count = n())

print(count_measures)

# Step 2: Calculate the interval between measurements for each plot, ecozone, and source

intervals <- ecolevol_wide4 %>%

arrange(ecozone, sources, magp_site_id, meas_year) %>%

group_by(ecozone, sources, magp_site_id) %>%

mutate(year_diff = c(NA, diff(meas_year))) %>%

ungroup() %>%

select(ecozone, sources, magp_site_id, meas_year, year_diff)

# Combining the count of measurements with the intervals

ecozone_freq <- count_measures %>%

left_join(intervals, by = c("ecozone", "sources", "magp_site_id"))

## Save the table to a CSV file

file_path <- "C:/Users/martigau/OneDrive - NRCan RNCan/Documents/Personnel/Travail/EcolEvol/NFI Data/ecozone_freq.csv"

write.csv(ecozone_freq, file = file_path)

write.csv2(ecozone_freq, 'ecozone_freq_fr.csv', row.names = FALSE)

# Step 3: Exclude NAs from year_diff and calculate summary statistics by ecozone and source

summary_ecozone <- ecozone_freq %>%

filter(!is.na(year_diff)) %>%

group_by(ecozone, sources) %>%

summarise(

min_measurement_count = min(measurement_count),

max_measurement_count = max(measurement_count),

median_measurement_count = median(measurement_count),

mean_measurement_count = mean(measurement_count),

min_year_diff = min(year_diff),

max_year_diff = max(year_diff),

median_year_diff = median(year_diff),

mean_year_diff = mean(year_diff)

)

# View the result

print(summary_ecozone)

## Save the table to a CSV file

file_path <- "C:/Users/martigau/OneDrive - NRCan RNCan/Documents/Personnel/Travail/EcolEvol/NFI Data/summary_ecozone.csv"

write.csv(summary_ecozone, file = file_path)

write.csv2(summary_ecozone, 'summary_ecozone_fr.csv', row.names = FALSE)

# Step 1: Count how many times each plot has been measured for each ecozone and source

count_measures <- data %>%

group_by(ecozone, sources, magp_site_id) %>%

summarise(measurement_count = n())

# Step 2: Calculate the interval between measurements for each plot, ecozone, and source

intervals <- data %>%

arrange(ecozone, sources, magp_site_id, meas_year) %>%

group_by(ecozone, sources, magp_site_id) %>%

mutate(year_diff = c(NA, diff(meas_year))) %>%

ungroup() %>%

select(ecozone, sources, magp_site_id, meas_year, year_diff)

# Combine the count of measurements with the intervals

combined_data <- count_measures %>%

left_join(intervals, by = c("ecozone", "sources", "magp_site_id"))

# Step 3: Exclude NAs from year_diff and calculate summary statistics by ecozone and source

summary_stats <- combined_data %>%

filter(!is.na(year_diff)) %>%

group_by(ecozone, Sources) %>%

summarise(

min_measurement_count = min(measurement_count),

max_measurement_count = max(measurement_count),

median_measurement_count = median(measurement_count),

mean_measurement_count = mean(measurement_count),

min_year_diff = min(year_diff),

max_year_diff = max(year_diff),

median_year_diff = median(year_diff),

mean_year_diff = mean(year_diff)

)

# View the summary statistics

print(summary_stats)

#######################################################

# HARVESTING INTENSITY BY ECOZONE AND TREATMENT CLASS #

#######################################################

# Load dataset

ecolevol_wide6 <- fread("C:/Users/martigau/OneDrive - NRCan RNCan/Documents/Personnel/Travail/EcolEvol/NFI Data/ecolevol_wide6.csv")

colnames(ecolevol_wide6)

str(ecolevol_wide6)

# How many plots per ecozone and treatment class?

plots_by_ecozone_cat <- ecolevol_wide6 %>%

dplyr::select(-V1) %>%

group_by(ecozone, category) %>%

summarize(count_magp_site_id = n_distinct(magp_site_id), .groups = 'drop')

print(plots_by_ecozone_cat)

#Harvesting intensity and time since trt by ecozone and category

intensity_by_ecozone_cat <- ecolevol_wide6 %>%

dplyr::select(-V1) %>%

filter(time_since_trt > 0) %>%

group_by(ecozone, category) %>%

summarize(

count_magp_site_id = n_distinct(magp_site_id),

min_treatment_year2 = min(treatment_year2, na.rm = TRUE),

max_treatment_year2 = max(treatment_year2, na.rm = TRUE),

mean_treatment_year2 = mean(treatment_year2, na.rm = TRUE),

min_treatment_pct_y = min(treatment_pct.y, na.rm = TRUE),

max_treatment_pct_y = max(treatment_pct.y, na.rm = TRUE),

mean_treatment_pct_y = mean(treatment_pct.y, na.rm = TRUE),

min_time_since_trt = min(time_since_trt, na.rm = TRUE),

max_time_since_trt = max(time_since_trt, na.rm = TRUE),

mean_time_since_trt = mean(time_since_trt, na.rm = TRUE),

.groups = 'drop'

)

print(intensity_by_ecozone_cat)

## Save the table to a CSV file

file_path <- "C:/Users/martigau/OneDrive - NRCan RNCan/Documents/Personnel/Travail/EcolEvol/NFI Data/intensity_by_ecozone_cat.csv"

write.csv(intensity_by_ecozone_cat, file = file_path)

write.csv2(intensity_by_ecozone_cat, 'intensity_by_ecozone_cat_fr.csv', row.names = FALSE)
